# Structure-Based Design of Transport-Specific Multitargeted One-Carbon Metabolism Inhibitors in Cytosol and Mitochondria

**DOI:** 10.1021/acs.jmedchem.3c00763

**Published:** 2023-08-15

**Authors:** Md. Junayed Nayeen, Jade M. Katinas, Tejashree Magdum, Khushbu Shah, Jennifer E. Wong, Carrie E. O’Connor, Alexandra N. Fifer, Adrianne Wallace-Povirk, Zhanjun Hou, Larry H. Matherly, Charles E. Dann, Aleem Gangjee

**Affiliations:** † Division of Medicinal Chemistry, Graduate School of Pharmaceutical Sciences, Duquesne University, Pittsburgh, Pennsylvania 15282, United States; ‡ Department of Oncology, Wayne State University School of Medicine, Detroit, Michigan 48201, United States; § Molecular Therapeutics Program, Barbara Ann Karmanos Cancer Institute, 4100 John R, Detroit, Michigan 48201, United States; ∥ Department of Chemistry, Indiana University, Bloomington, Indiana 47408, United States

## Abstract

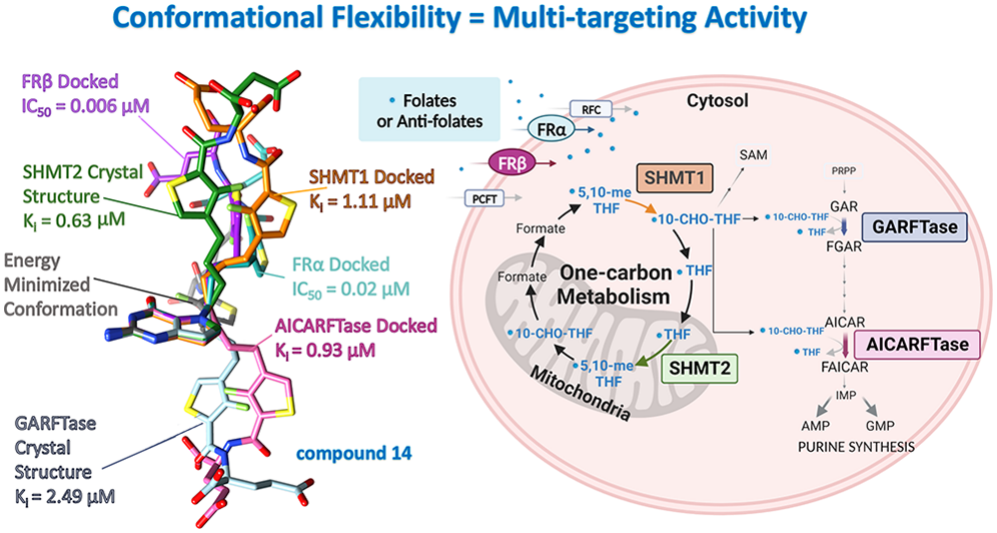

Multitargeted agents provide tumor selectivity with reduced drug resistance and dose-limiting toxicities. We previously described the multitargeted 6-substituted pyrrolo[3,2-*d*]pyrimidine antifolate **1** with activity against early- and late-stage pancreatic tumors with limited tumor selectivity. Structure-based design with our human serine hydroxymethyl transferase (SHMT) 2 and glycinamide ribonucleotide formyltransferase (GARFTase) structures, and published X-ray crystal structures of 5-aminoimidazole-4-carboxamide ribonucleotide formyltransferase/inosine monophosphate cyclohydrolase (ATIC), SHMT1, and folate receptor (FR) α and β afforded 11 analogues. Multitargeted inhibition and selective tumor transport were designed by providing promiscuous conformational flexibility in the molecules. Metabolite rescue identified mitochondrial C1 metabolism along with *de novo* purine biosynthesis as the targeted pathways. We identified analogues with tumor-selective transport via FRs and increased SHMT2, SHMT1, and GARFTase inhibition (28-, 21-, and 11-fold, respectively) compared to **1**. These multitargeted agents represent an exciting new structural motif for targeted cancer therapy with substantial advantages of selectivity and potency over clinically used antifolates.

## Introduction

Single-target inhibition in cancer is all too often insufficient for tumor eradication. The heterogeneous nature of most cancers enables development of resistance to individual therapeutic agents. Further, currently used anticancer agents are generally not tumor-specific. Rather, they attack normal cells as well as tumors and, as a result, dose-limiting toxicities often limit their clinical effectiveness.^1^ Indeed, a general lack of tumor selectivity and consequent toxicities combined with chemotherapy resistance significantly contribute to the failure of most antitumor therapies.

The combination of cancer chemotherapy with multiple drugs and divergent tumor targets has resulted in significant progress in overcoming classical resistance to therapy.^2^ Further, judicious combinations of multiple agents that do not possess overlapping toxicities provide relief from the toxic burden of therapy. However, difficulties persist with this approach, reflecting an inability to precisely time the impact of individual drugs on the tumor to maximize response, reflecting differing pharmacokinetics and ADMET properties.^2^ Many of these challenges can be circumvented with a tumor-selective, multitargeted agent that simultaneously inhibits multiple tumor targets with predictable ADMET and a single toxicity profile.^3,4^

**Figure 1:**
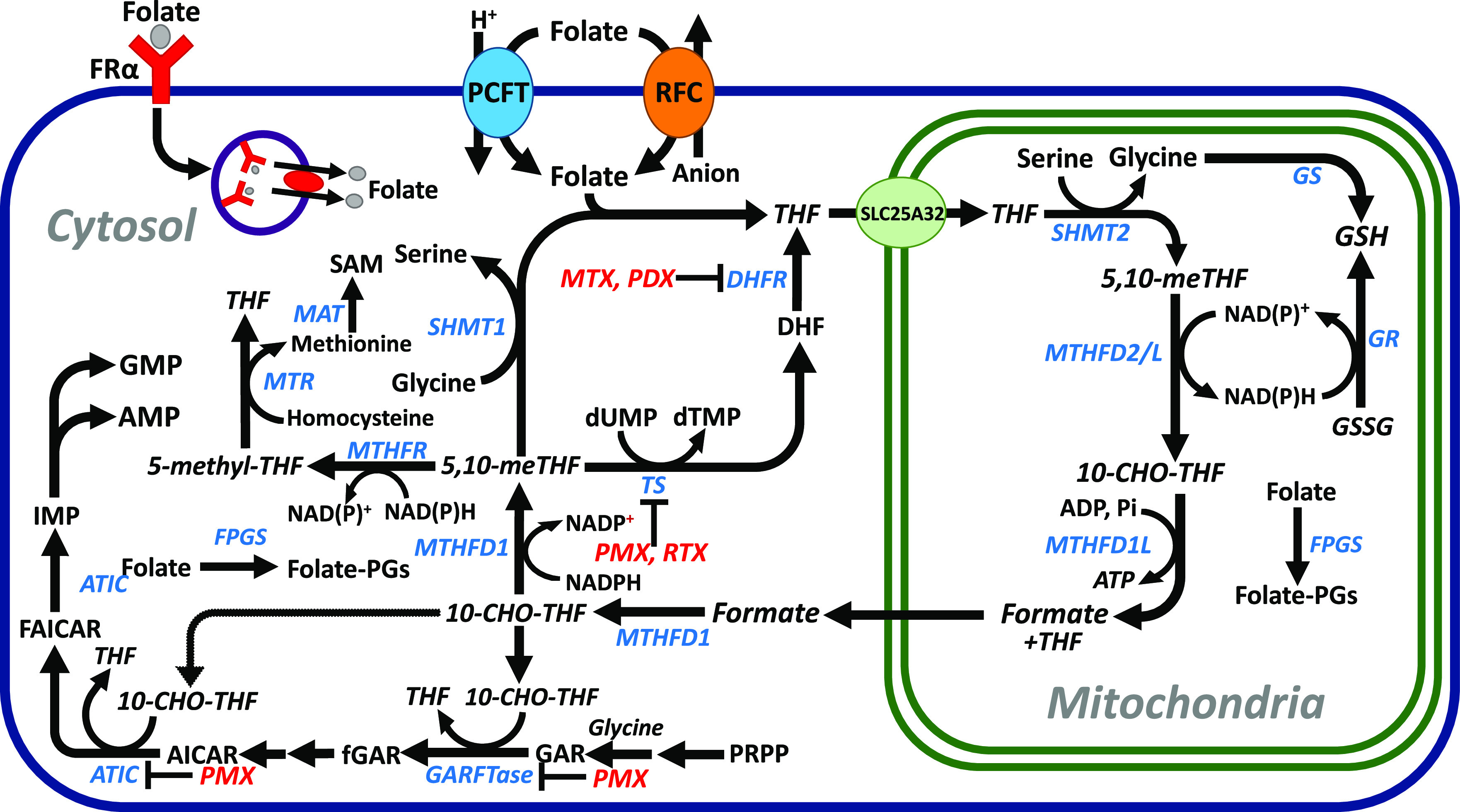
C1 metabolism. The schematic depicts facilitated transport via RFC or PCFT, or by endocytosis via FRα. Following internalization, folates are metabolized to folate polyglutamates. Cytosolic folates are transported into the mitochondria by SLC25A32. In the mitochondria, serine is catabolized by SHMT2, MTHFD2/L, and MTHFD1L to generate glycine, nicotinamide adenine dinucleotide (NADH), adenosine triphosphate (ATP), and formate. Formate passes to the cytosol and is converted to 10-formyl tetrahydrofolate (THF), which is further metabolized to 5,10-methylene THF and 5-methyl THF. Cytosolic targets for clinically approved antifolates (MTX, PDX, PMX, and RTX) are indicated. Abbreviations are as follows: 10-CHO-THF, 10-formyl THF; 5,10-me-THF, 5,10-methylene THF; AICAR, 5-aminoimidazole-4-carboxamide ribonucleotide; ATIC, AICAR formyltransferase/inosine monophosphate cyclohydrolase; DHF, dihydrofolate; DHFR, dihydrofolate reductase; FAICAR, formyl 5-aminoimidazole-4-carboxamide ribonucleotide; fGAR, formyl glycinamide ribonucleotide; FPGS, folylpolyglutamate synthetase; GAR, glycinamide ribonucleotide; GARFTase, glycinamide ribonucleotide formyltransferase; GR, glutathione reductase; GS, glutathione synthetase; GSH, glutathione; MTHFD1, 5,10-methylene THF dehydrogenase 1; MTHFD1L, 5,10-methylene THF dehydrogenase 1-like; MTHFD2, 5,10-methylene THF dehydrogenase 2; MTHFD2L, 5,10-methylene THF dehydrogenase 2-like; MTHFR, 5,10-methylene THF reductase; MTR, methionine synthase; PCFT, proton-coupled folate transporter; PGs, polyglutamates; PRPP, phosphoribosyl pyrophosphate; RFC, reduced folate carrier; SAM, *S*-adenosylmethionine; SHMT1/2, serine hydroxymethyl transferase 1/2; TS, thymidylate synthase. Adapted from Wallace-Povirk et al.^13^

Folate-dependent one-carbon (C1) metabolism is essential for the biosynthesis of nucleotides and key amino acids (e.g., serine, methionine) required for cell homeostasis (Figure 1). In mammalian cells, C1 metabolism is compartmentalized between the cytosol and mitochondria.^5,6^ Folates are transported into cells by facilitative transporters, the reduced folate carrier (RFC; SLC19A1)^7^ and the proton-coupled folate transporter (PCFT; SLC46A1),^8−10^ and by folate receptors (FRs) α and β, which function by endocytosis.^9,11^

RFC is ubiquitously expressed in normal and malignant cells in humans and is the major tissue folate transporter.^7^ PCFT is the principal transporter of folates in the upper gastrointestinal tract but shows a more limited tissue distribution than RFC.^8−10^ PCFT is widely expressed in human tumors and is active under acidic conditions characterizing the tumor microenvironment.^8,12^ FRα is expressed in normal epithelial tissues (e.g., mammary ducts, lungs, kidneys, choroid plexus) and a wide range of tumors (e.g., breast, cervical, renal, ovarian, endometrial cancers); FRβ is expressed in hematopoietic tissues, activated myeloid cells, and tumor-associated macrophages (TAMs).^11,13−15^

Antifolates have been used for decades for cancer. Clinically used antifolates include methotrexate (MTX), pemetrexed (PMX), pralatrexate (PDX), and raltitrexed (RTX).^12,16^ MTX and PDX are both inhibitors of dihydrofolate reductase (Figure 1); MTX is primarily used to treat acute lymphoblastic leukemia and osteosarcoma, whereas PDX is FDA-approved for treating peripheral T-cell lymphoma. PMX and RTX are thymidylate synthase inhibitors; PMX also inhibits dihydrofolate reductase and *de novo* purine biosynthesis at glycinamide ribonucleotide (GAR) formyltransferase (GARFTase) and 5-aminoimidazole-4-carboxamide ribonucleotide (AICAR) formyltransferase (AICARFTase) (in the bifunctional enzyme AICARFTase/inosine monophosphate cyclohydrolase (ATIC)) (Figure 1).^16,17^

**Figure 2:**
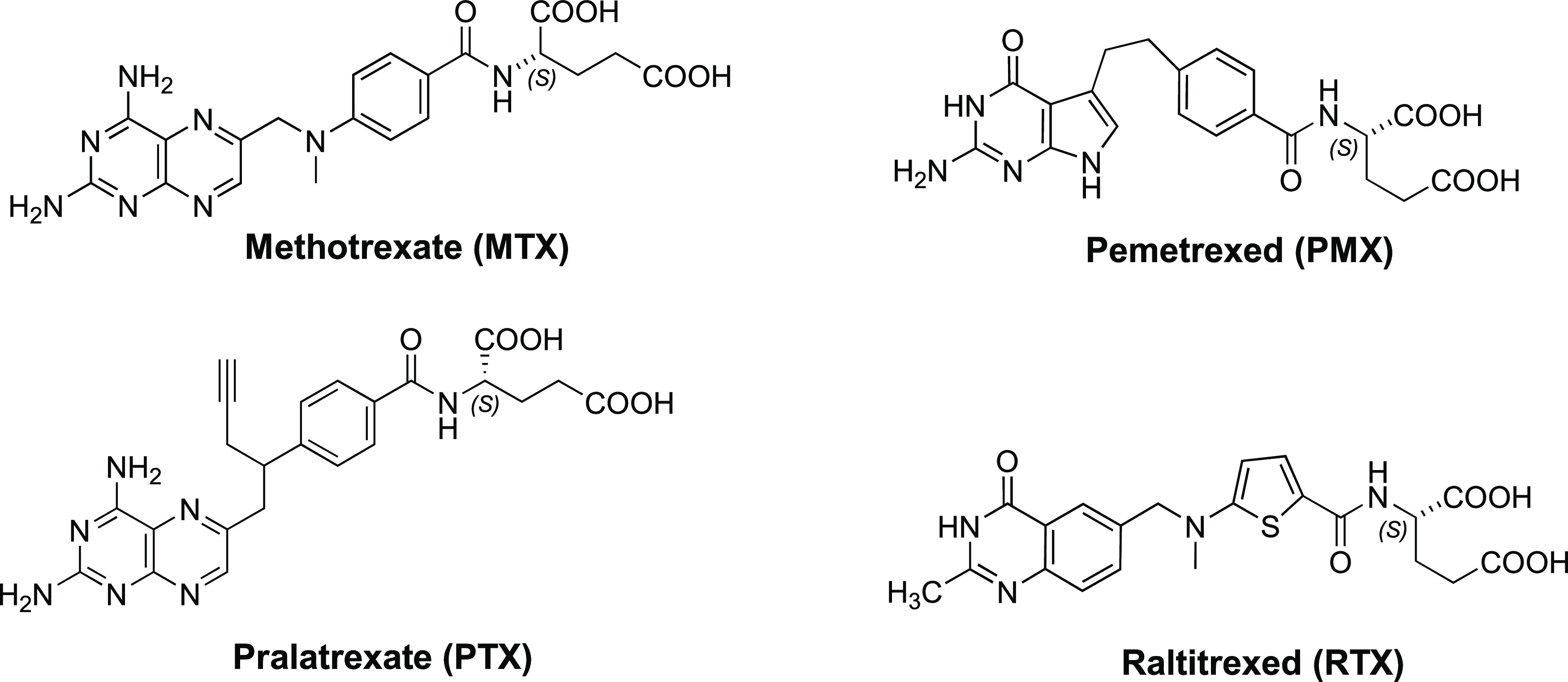
Structures of the clinically used classical antifolates.

All of the clinically used antifolates (Figure 2) are transported by RFC.^12,16^ Indeed, drug transport by RFC by normal tissues is a major cause of dose-limiting toxicities for all of the clinically used antifolates. Further loss of RFC transport is a common mechanism of antifolate resistance.^7,16^ This realization has prompted extensive studies on the design of a new generation of tumor-targeted agents (e.g., targeted antifolates, cytotoxic folate conjugates, and antibody–drug conjugates) with selective uptake by FRα and FRβ, as well as PCFT.^8,11−13,18,19^ Ideally, these agents would show limited uptake by RFC.

Following internalization, cytosolic folates are transported into mitochondria by SLC25A32.^20^ Although serine, glycine, and formate exchange between mitochondria and cytosol,^6^ mitochondrial folates do not exchange with folates in the cytosol.^20^ In the mitochondria, serine hydroxymethyl transferase (SHMT) 2 catalyzes the conversion of serine and tetrahydrofolate (THF) to glycine and 5,10-methylene THF (Figure 1).^6,21^ 5,10-Methylene THF is subsequently metabolized by MTHFD2 (5,10-methylene THF dehydrogenase 2) to 10-formyl THF, which is converted to formate by MTHFD1L (5,10-methylene THF dehydrogenase 1-like).^6,21^ In the cytosol, MTHFD1 (5,10-methylene THF dehydrogenase 1) catalyzes the synthesis of 10-formyl THF from formate and THF. 10-Formyl THF is the C1 donor for *de novo* purine biosynthesis in reactions catalyzed by the GARFTase and AICARFTase activities of the bifunctional enzyme ATIC (Figure 1). 10-Formyl THF is also converted to 5,10-methylene THF by MTHFD1 for thymidylate synthase and other reactions including SHMT1 and 5,10-methylene THF reductase. In cancer cells, serine is the primary source of C1 units for the biosynthesis of purines and thymidylate in the cytosol and accounts for >85% of glycine for the synthesis of proteins, purines, heme, and glutathione.^6,21^ In addition, mitochondrial C1 metabolism from serine is also an important source of NAD(P)H and ATP.^6,21^

Importantly, serine catabolism is frequently upregulated in cancer, with SHMT2 and MTHFD2 among the most overexpressed metabolic genes in human cancers compared to normal tissues.^22^ Indeed, studies in several cancers suggest that SHMT2 is a bona fide “onco-driver”.^23−26^ Further, overexpression of SHMT2 is associated with poor prognosis in breast cancer,^27−29^ lung adenocarcinoma,^30^ pancreatic cancer,^31^ gliomas,^32^ and gastrointestinal cancers.^33^ In invasive breast cancer, adrenal carcinoma, and renal cell carcinomas, SHMT2 is likewise overexpressed.^27^ Collectively, these studies argue that SHMT2 is a promising therapeutic target for a broad range of cancers including late-stage tumors.^21,34^

However, the challenges in targeting C1 metabolism can be formidable. These range from limited tumor selectivity (reflecting the importance of particular C1 pathways to both tumors and normal tissues) to the lack of effective tumor targeting via tumor-selective uptake by FRs^18^ or PCFT^8^ over the ubiquitously expressed RFC.^7^ Further, for SHMT2, loss of activity results in a compensatory reversal of SHMT1 catalysis (serine → glycine),^35^ thus limiting the metabolic impact. Indeed, an ideal C1 inhibitor can be envisaged to inhibit multiple cellular targets in both the mitochondria and cytosol, while preserving tumor selectivity via uptake by FRs and/or PCFT over RFC.

**Figure 3:**
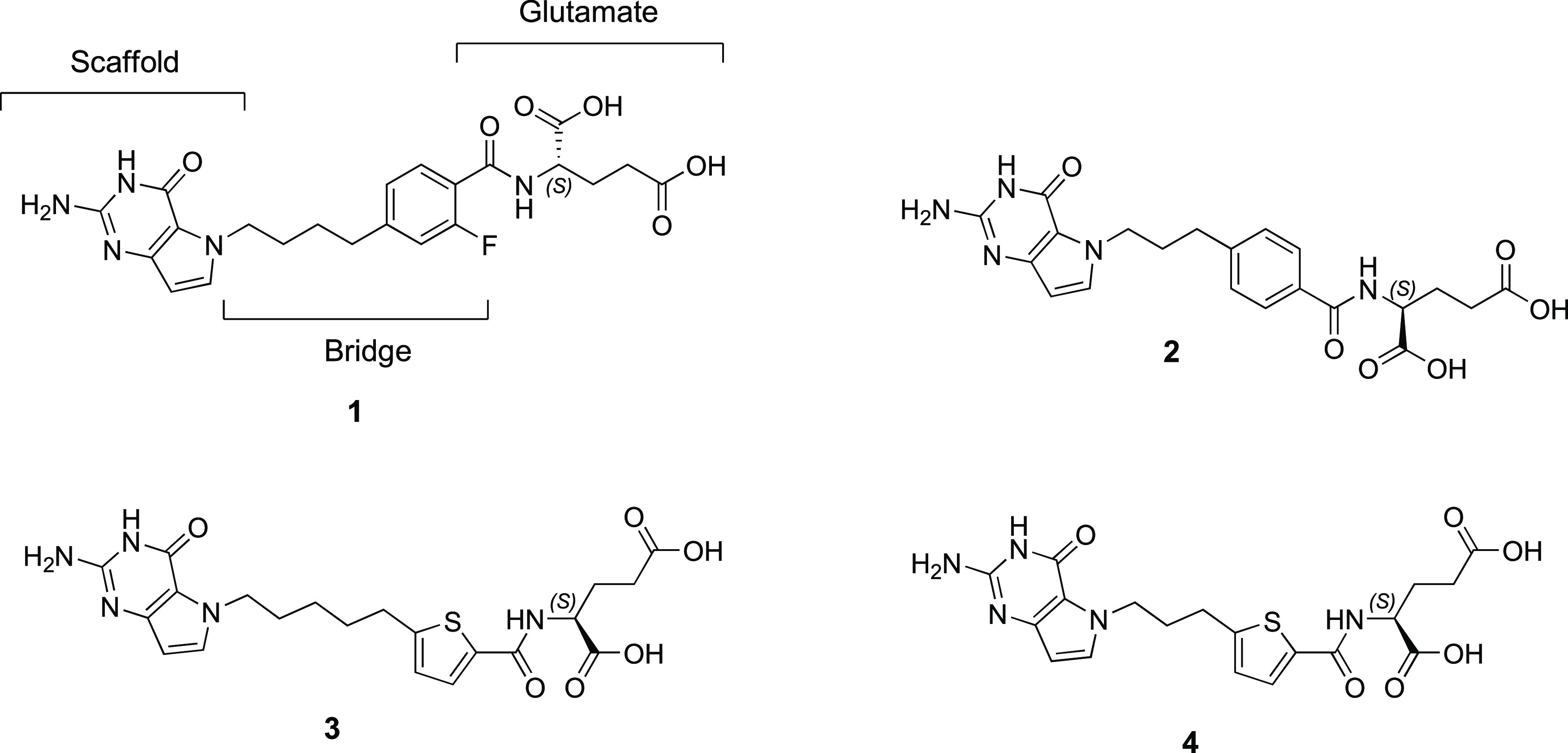
Structures of the 5-substituted pyrrolo[3,2-*d*]pyrimidine antifolates with phenyl (**1** and **2**) and thienyl (**3** and **4**) l-glutamates and 3–5 bridge carbons.^36^

We recently discovered potent, multitargeted antifolates **1**–**4**, all 5-substituted pyrrolo[3,2-*d*]pyrimidine compounds (Figure 3).^36^ The most active compound **1** was transported into tumor cells by RFC and PCFT, and accumulated in both mitochondria and cytosol as polyglutamates which act as potent inhibitors of mitochondrial (SHMT2) and cytosolic (SHMT1, GARFTase, and AICARFTase^36^) C1 targets.^5^ Compound **1** showed moderate *in vitro* antitumor efficacy toward multiple tumor types, including pancreatic cancer, lung adenocarcinoma, and colon cancer cell lines,^5,36^ and showed potent *in vivo* efficacy against both early- and late-stage MIA PaCa 2 pancreatic cancer xenografts.^36^ Compound **1** is a first-in-class “classical” antifolate agent that inhibits multiple cellular targets in C1 metabolism in both the mitochondria and cytosol.

In this report, we used compounds **1**–**4** as lead analogues to explore the structure–activity relationships (SAR) for 11 new compounds of this series as a means of optimizing transporter selectivity and increasing multitarget potency and specificity. To optimize and validate target inhibition, we determined the X-ray structures for human SHMT2 in complex with **1** and human GARFTase complexed with **1**, **2**, and **3**. In the design of our multitargeted C1 inhibitors, we used molecular modeling based on these structures for SHMT2 and GARFTase, along with published X-ray crystal structures of human ATIC,^37^ rabbit SHMT1,^19^ human FRα,^38^ and human FRβ.^39^

## Results and Discussion

### Design of Multitargeted Tumor-Selective Antifolates

#### Rationale

Compounds **1** (4-atom bridge), **2** (3-atom bridge), and **3** (5-atom bridge) (Figure 3) were the first reported classical antifolates to be actively transported into cells by RFC and PCFT and to inhibit mitochondrial and cytosolic C1 metabolic targets.^36^ In the design of multitargeted single agents, it is important to provide flexibility since this affords the conformational promiscuity required to bind to multiple cellular targets. On the other hand, rigidity restricts flexibility and precludes multitarget attributes.^40^

**Figure 4:**
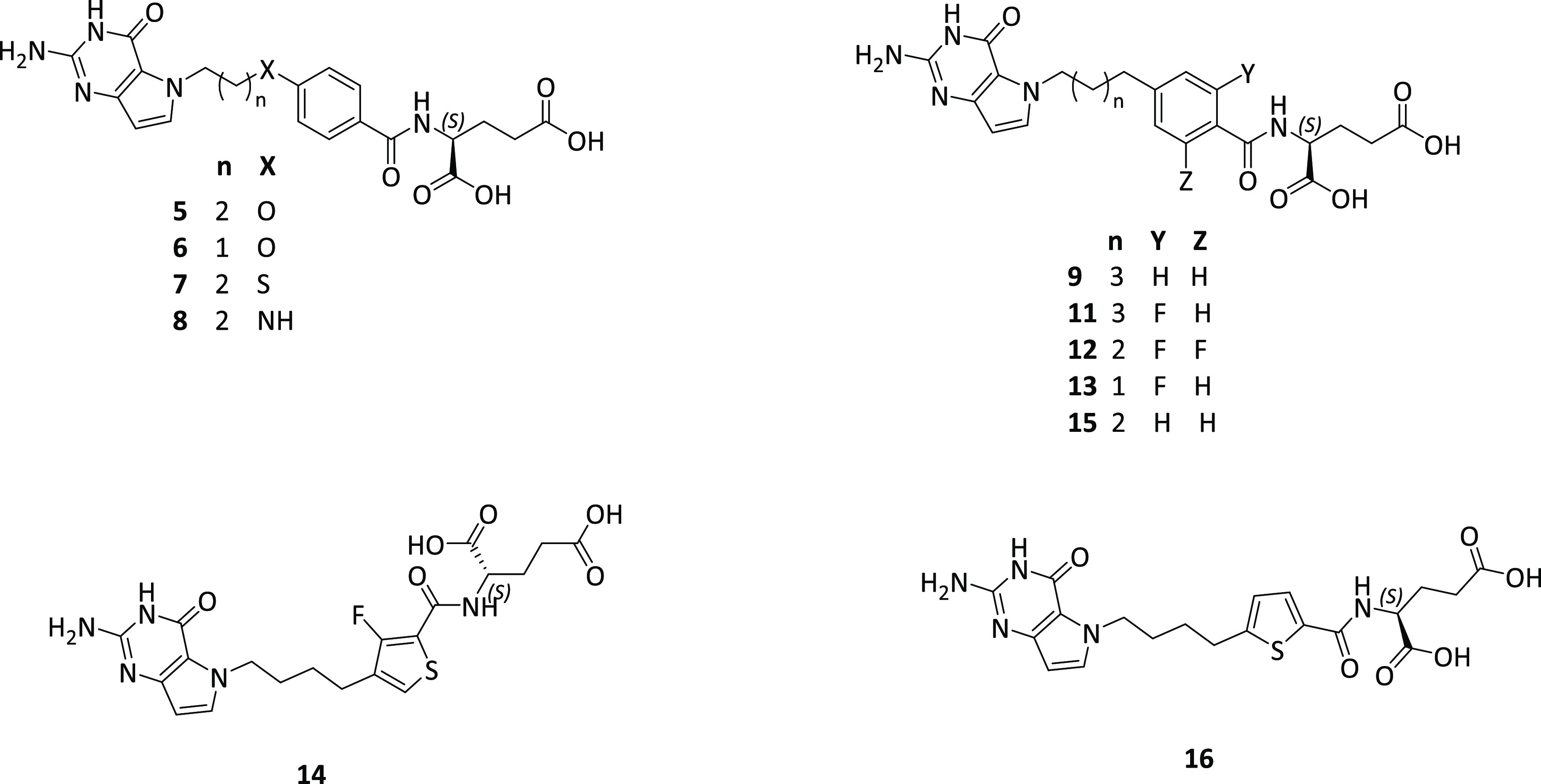
Designed 5-substituted pyrrolo[3,2-*d*]pyrimidine analogues.

For our proposed pyrrolo[3,2-*d*]pyrimidine compounds (**5**–**9**, **11**–**16**) (Figure 4), we used a “scaffold hopping” approach for the bridge phenyl in the systematic design and synthesis of multitargeted analogues with variations involving the bridge ring (phenyl, thiophene, and fluorine substitutions). We introduced ample flexibility by incorporating chain lengths of 3-, 4-, and 5-carbon atoms into the bridge region, which allows the carboxylate and the bicyclic ring scaffold at the two ends of the molecule to adopt appropriate conformations for binding multiple sites on putative protein targets.

Judicious conformational restriction confined to the phenyl ring and the l-glutamate moieties can be used to enhance the potencies of classical antifolates. For instance, Pendergast et al.^41^ described conformational restriction of the l-glutamate via a fluorine–hydrogen bond. We previously adopted this approach to 6-substituted pyrrolo[2,3-*d*]pyrimidine antifolate analogues.^42^ Thus, introducing fluorine into bioactive molecules results in additional hydrophobic interactions with receptors and transporters, as well as enzyme targets, and also confers beneficial changes in drug pharmacodynamics.^43,44^ Based on this, we incorporated fluorine into the bridge aromatic ring of five of the proposed pyrrolo[3,2-*d*]pyrimidine analogues including **1** (Figure 4), to compare effects on biological activities of the fluorine-substituted compounds with the corresponding des-fluoro analogues.

**Figure 5:**
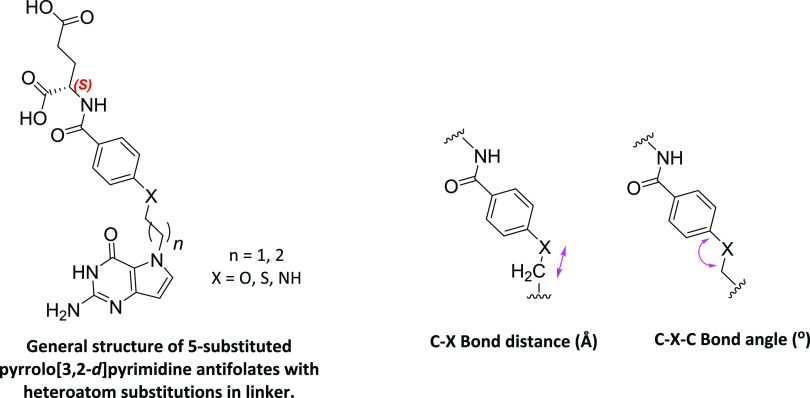
General structures of 5-substituted pyrrolo[3,2-*d*]pyrimidine antifolates including heteroatom bridge substitutions with distance and bond angle variations. (Table 1).

In our previous study of 6-substituted pyrrolo[2,3-*d*]pyrimidine antifolates,^38^ we demonstrated substantial inhibition of KB human tumor cell proliferation associated with cellular uptake by FRα and PCFT, accompanied by a significant reduction in RFC transport. In addition, we showed that differences in biological activity could be attributed to varying the C–X bond distances and C–X–C bond angles (X = heteroatom) in the linker.^38^ The C–X bond lengths of these compounds follow the trend C–O < C–NH < C–CH_2_ < C–S (Figure 5). The C–X–C bond angle follows the trend C–S–C < C–CH_2_–C < C–NH–C < C–O–C. Thus, replacing the benzylic CH_2_ can vary the bond angle and/or bond length (Table 1), structural alterations that are expected to positively impact transport specificity and target enzyme inhibition. Based on this, we designed a series of 5-substituted pyrrolo[3,2-*d*]pyrimidine compounds with 4 bridge atoms to evaluate and compare the effects of replacing the benzylic CH_2_ with O (**5** and **6**), S (**7**), and NH (**8**) (Figure 4).

**Table 1:** Distances and Bond Angle Variations for **4** Atom Bridge Pyrrolo[3,2-*d*]pyrimidine Antifolates Predicted by the Atom of the Bridge at the Benzylic Position (X)^a^

compound	X	C–X bond distance (Å)	C–X–C bond angle (Å)
**9**	CH_2_	2.63	109.5
**5**	O	2.42	126.1
**7**	S	2.88	99
**8**	NH	2.52	111

a Bond angles for X = CH_2_, O, and S obtained from the literature.^45^ Distances and angles for X = NH were measured using energy-minimized conformations of compounds with Maestro12.1. (Maestro, Schrödinger, LLC, New York, NY, 2021).

### Design of Multitargeted Transport-Specific Inhibitors

An important goal of this study was to optimize the binding of novel 5-substituted pyrrolo[3,2-*d*]pyrimidine compounds to both cytosolic SHMT1 and mitochondrial SHMT2, while improving tumor selectivity by circumventing RFC transport and increasing transport by other uptake processes (e.g., FRs). For SHMT2, X-ray crystal structures were previously reported by Scaletti et al.^46^ in complex with lometrexol (6QVG) or PMX (6QVl), both exceedingly poor inhibitors of this enzyme. Although Ducker et al.^47^ published a human SHMT2 crystal structure with 6-amino-4-[3-(hydroxymethyl)-5-phenylphenyl]-3-methyl-4-propan-2-yl-2*H*-pyrano[2,3-*c*]pyrazole-5-carbonitrile (SHIN1) (PDB ID: 5V7I), this nonclassical antifolate lacks an l-glutamate, which is an important binding determinant for classical antifolates for both transport and target inhibition. As these SHMT2 structures were not ideal for molecular modeling and compound design, we determined the X-ray crystal structure of human SHMT2 with **1** (PDB ID: 8FJU) and used this structure to design additional analogues of this series. For molecular modeling of our compounds with SHMT1, we utilized the rabbit (*Oryctolagus cuniculus*) (PDB ID: 1LS3)^19^ SHMT1 X-ray crystal structure.

As our aim was to optimize multitargeted single agents, we also performed molecular modeling with putative cytosolic targets in *de novo* purine biosynthesis using the published crystal structures of human GARFTase (PDB ID: 5J9F)^48^ and human ATIC (PDB ID: 1P4R).^37^ To optimize the transport of our designed analogues, we used the X-ray crystal structures of human FRα in complex with our novel antifolate *N*-(4-{[2-(2-amino-4-oxo-4,7-dihydro-3*H*-pyrrolo[2,3-*d*]pyrimidin-6-yl)ethyl]amino} benzene-1-carbonyl)-l-glutamic acid (**10**) (PDB ID 5IZQ)^38^ and human FRβ in complex with PMX (PDB ID: 4KN2).^39^ The docked scores for the docked compounds with putative enzyme and transport targets are summarized in Table S1 (Supplemental Information).

#### X-ray Crystal Structure of **1** and Molecular Modeling with Human SHMT2

**Figure 6:**
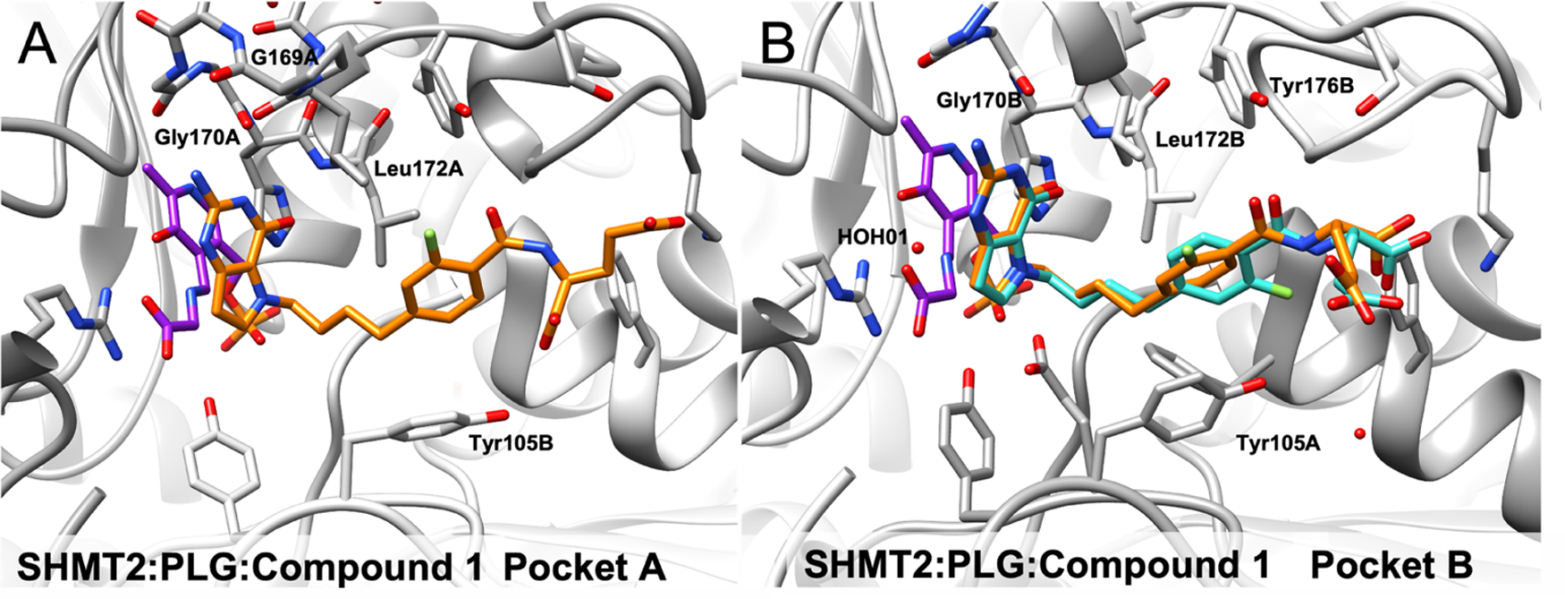
Structural analysis of 5-substituted pyrrolo[3,2-*d*]pyrimidine 1 in SHMT2-PLP-gly/PLG-antifolate ternary complexes. (A) Crystal structures of **1** (orange) bound in the THF binding pocket of both pockets of the asymmetric unit of the SHMT2 dimer with the natural cofactor PLP (purple). **1** occurs in a single conformation (conformation A) in pocket A. (B) Crystal structures of **1** (orange) with the natural cofactor PLP (purple) in two conformations (orange and teal) in pocket B (conformation A had an occupancy of 41% and conformation B had an occupancy of 59%).

Pyridoxal phosphate (PLP)-loaded SHMT2 was co-crystallized with inhibitor **1** and serine. The SHMT2 crystal structure in complex with **1** and PLP was solved to a resolution of 2.51 Å, (Figure 6). Data processing and refinement statistics (Table S2), along with detailed interactions and maps for the complex (Figure S1), are included in the Supplemental Information. In both pockets of the SHMT2-compound **1** structure, there was an adduct of PLP bound to glycine (labeled PLG) (Figure 6A, represented in purple). From the crystal structures, the fluoro-phenyl bridge in **1** adopts an orthogonal conformation to the pyrrolo[3,2-*d*]pyrimidine scaffold. The electron density supported modeling **1** in a single conformation in one pocket (pocket A) but in two distinct conformations in the second pocket (pocket B). One of the conformations in pocket B was similar to the conformation in pocket A, suggesting flexibility in the orientation of **1** when interacting with the enzyme. Specifically, the l-glutamyl tail of **1** in each pocket has distinctly different orientations.

The pyrrolo[3,2-*d*]pyrimidine scaffold of **1** makes conserved polar contacts with the backbone atoms of Gly170 and Leu172 in SHMT2. Compound **1** forms an additional contact between the N11 of the pyrrolo[3,2-*d*]pyrimidine ring system and the carbonyl oxygen of the Gly169 backbone in binding pocket A (Figures 6A and S1, Supplemental Information). The N1 of the pyrrolo[3,2-*d*]pyrimidine scaffold coordinates with a conserved water molecule (Figures 6B and S1, Supplemental Information). The α-COOH of the l-glutamyl tail of **1** makes a polar interaction with Tyr105 in pocket A and forms contact with the fluorine in the additional conformation (pocket B). There is another polar contact with the carbonyl of the l-glutamyl tail of **1** and the Tyr176 hydroxyl.

**Figure 7:**
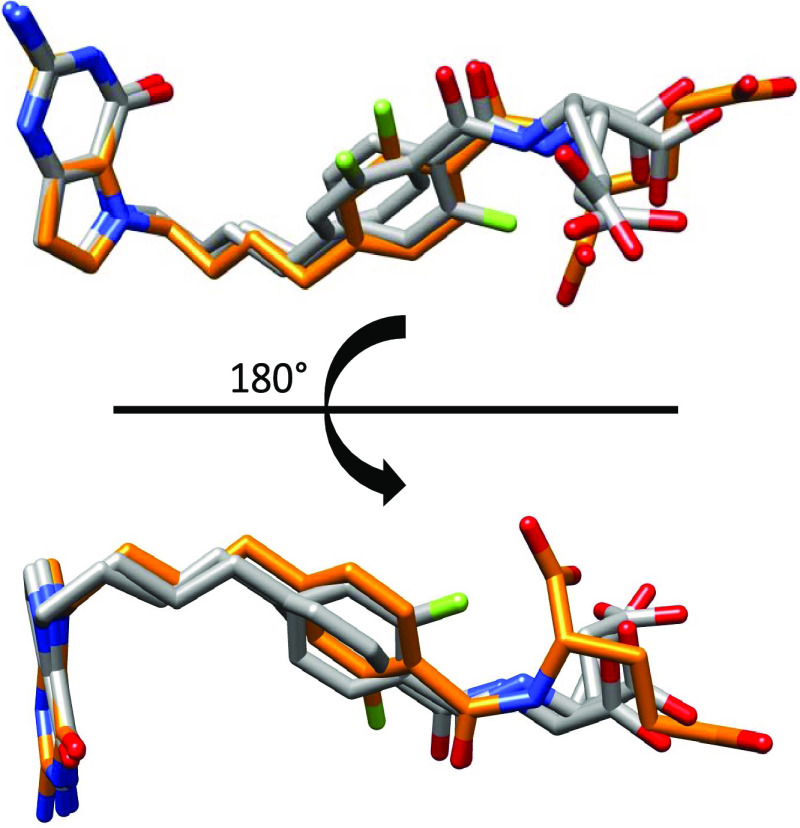
Superposition of **1** in each binding pocket of crystallized SHMT2. Superposition of crystallized **1** in pocket A (orange) and pocket B (dark gray) in SHMT2.

The fluoro-phenyl ring of **1** adopts three different conformations in the two binding pockets of SHMT2. In pocket A, the fluorine on the phenyl ring and O18 of the amide-carbonyl reside on the same side. As noted above, one conformation in pocket B is similar to the conformation in pocket A. For the other conformation in pocket B, the amide NH (N19) resides on the same side as the fluorine (Figures 7 and S1, Supplemental Information). This hydrogen bond interaction between the fluorine and the amide N19-H may lock the orientation of the l-glutamate tail in a specific conformation and is predicted to enhance the binding affinity of **1**.

**Figure 8:**
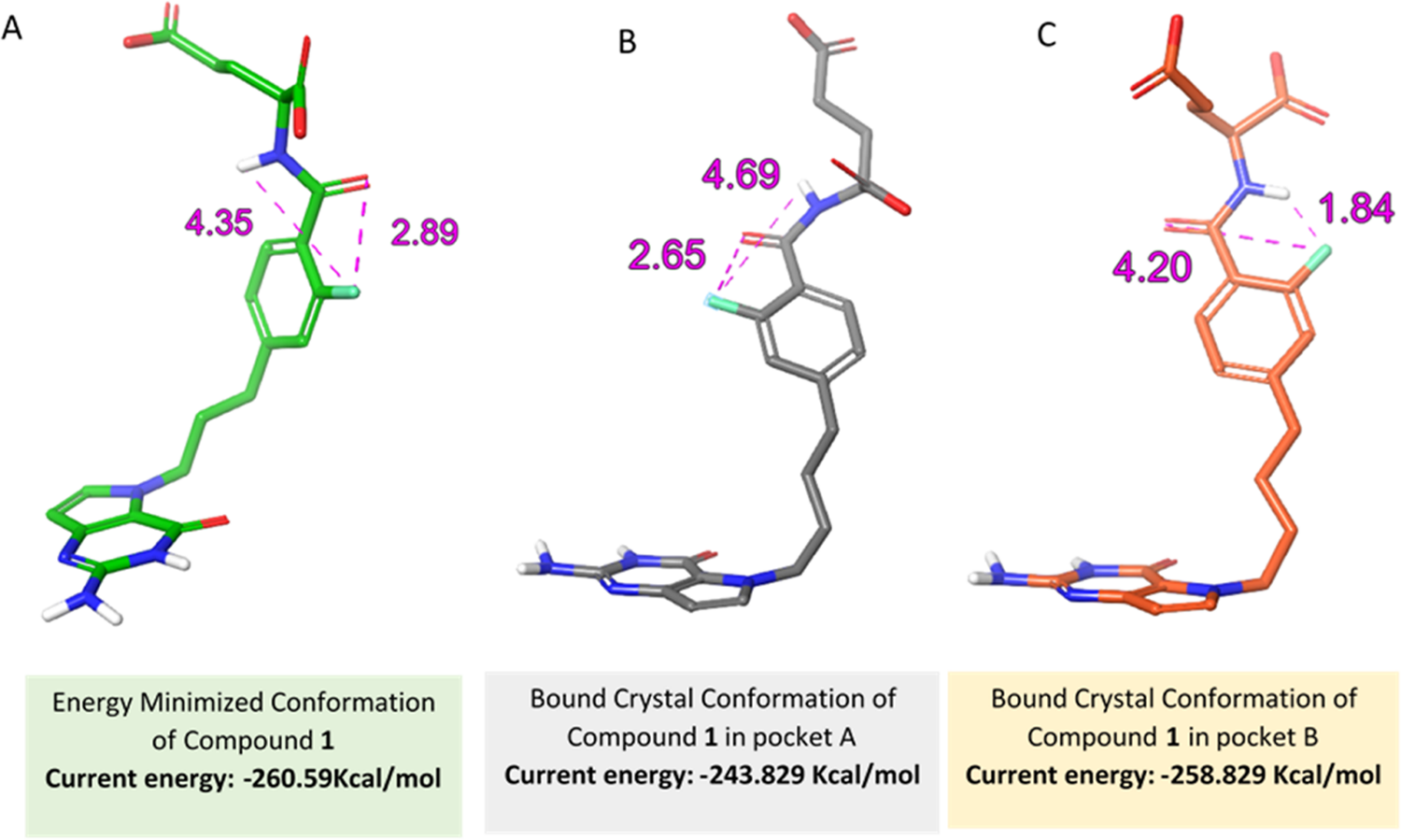
Conformations of **1** in SHMT2 structure. Comparison of conformations of **1** in molecular modeling and X-ray crystal structures. (A) Energy-minimized structure of **1**. The distance between F–H–N– is 4.35 Å. The energy of this conformation is −260.6 kcal/mol. (B) Bound crystal structure of **1** in SHMT2 pocket A. The distance between F–H–N is 4.69 Å. The energy of this conformation is −243.8 kcal/mol. (C) Bound crystal structure of the alternate conformation of **1** in SHMT2 pocket B with the distance between F–H–N at 1.84 Å. The energy of this bound conformation is −258.8 kcal/mol.

We used the conformation in pocket A as the prototype to design additional SHMT2 inhibitors. From molecular modeling, it was determined that the energy-minimized conformation of **1** has the fluorine and O18 of the amide-carbonyl on the same side of the aromatic ring (Figure 8).

Human SHMT2 complexed with **1** (PDB ID: 8FJU) was used for *in situ* modeling of the designed compounds (Figure 4) and docking studies. The docked scores of the proposed compounds are summarized in Table S1 (Supplemental Information). The mitochondrial matrix has an alkaline pH (pH 7.7–7.9)^49,50^ compared to the cytosol (pH 7.0–7.4);^50,51^ for docking studies, the protein and our proposed ligands were prepared accordingly. From the docking studies, the 5-atom-bridged compounds showed preferential binding (−13.3 kcal/mol for **11**, −10.2 kcal/mol for **9**, and −10.0 kcal/mol for **3**) and the 3- to 4-atom-bridged compounds (**1**, **2**, **4**, **12**, **13**, **14**, **15**, **16**) showed slightly decreased docked scores (∼−9 kcal/mol). Analogues with heteroatoms in the bridge (**5**–**8**) were found to have poorer docked scores (−7.5 to −8.6 kcal/mol). We attribute these to the higher energy difference between the energy-minimized conformation and the docked conformation compared to the carbon-atom bridged analogues. The design of **12** with two fluorines at the ortho-positions of the phenyl ring to the carbonyl of the l-glutamate tail was based on the two distinct conformations of **1** bound to SHMT2 (Figure 6). As fluorine was observed to adopt positions with both the O18 and N19 in a syn orientation, we designed **12** with the ability to adopt both interactions (by providing two fluorines in one molecule).

#### Molecular Docking with SHMT1

For docking studies in SHMT1,^19^ the X-ray crystal structure of rabbit (*O. cuniculus*) SHMT1 crystallized with 5-formyl THF tri-glutamate (PDB ID: 1LS3)^19^ was used (Figure S2, Supplemental Information). From the conformation of the bound ligand, the bridge along with the phenyl adopts an orthogonal conformation to the pteridine scaffold. In our molecular docking studies with the pyrrolo[3,2-*d*]pyrimidine antifolates, the 5-atom bridged compounds bound in a similar conformation to 5-formyl THF tri-glutamate. From induced-fit docking, **1** adopts an orthogonal conformation in the binding pocket. Both the 2-NH_2_ and the N3 of the bicyclic ring form hydrogen bonds with the backbone carbonyl of Gly125; the 4-CO of the pyrimidine ring forms a hydrogen bond with the backbone NH of Leu127. The bridge remains within a good-contact proximity (range ≤ 4 Å)^52^ to make hydrophobic interactions with Tyr64, Leu121, Pro258, Phe131, and Leu127. Both the α- and γ-carboxylic acids of the l-glutamate of **1** form salt bridges with Lys134; the γ-carboxyl forms a water-mediated hydrogen bond with Ser254 as well.

For **3** and **9**, as well as related compounds, the interactions observed for the 5-formyl THF tri-glutamate scaffold were retained in the docked models (Figure S2, Supplemental Information). The N1 makes an interaction with the side chain of Asn347; the 2-NH_2_ interacts with the side chain of Asn347 and the backbone of Leu121 and the 3-NH interacts with the backbone of Gly125. Further, similar to 5-formyl THF tri-glutamate, the bridge aromatic ring (phenyl in **11** and thiophene in **3**) makes π–π interactions with Tyr64. The l-glutamates of both **9** and **3** are oriented differently from the corresponding tri-glutamate of the 5-formyl THF ligand, due to solvent exposure of the tri-glutamate linker and the presence of a free α-COOH of the first l-glutamate. For **9** and **3**, the α-COOH makes ionic interactions with Lys134B and the γ-COOH makes ionic interactions with both Lys134A and Lys134B. The 3-atom (**2**, **6**, and **13**) and 4-atom (**1** and **14**) bridged analogues maintained similar interactions with appropriate conformational adjustments due to a change in the bridge aromatic ring and bridge length (Figure S2, Supplemental Information). All of these molecules retained the orthogonal bound conformation observed for 5-formyl THF tri-glutamate and the other 5-atom bridged compounds. Docking studies of the two 5-atom bridged analogues **9** and **3** showed somewhat better docked scores (−12.3 and −11.9 kcal/mol, respectively) than for the 3- and 4-atom bridged compounds (−10.9 to −9.5 kcal/mol) (Table S1, Supplemental Information).

#### X-ray Crystal Structures and Molecular Modeling with GARFTase

We previously reported a GARFTase crystal structure in complex with a 6-substituted pyrrolo[2,3-*d*]pyrimidine **10** [(*S*)–2-({4-[3-(2-amino-4-oxo-4,7-dihydro-3*H*-pyrrolo[2,3-*d*]pyrimidin-6-yl)-propyl]-thiophene-2-carbonyl}-amino)-pentanedioic acid] (PDB ID: 5J9F).

**Figure 9:**
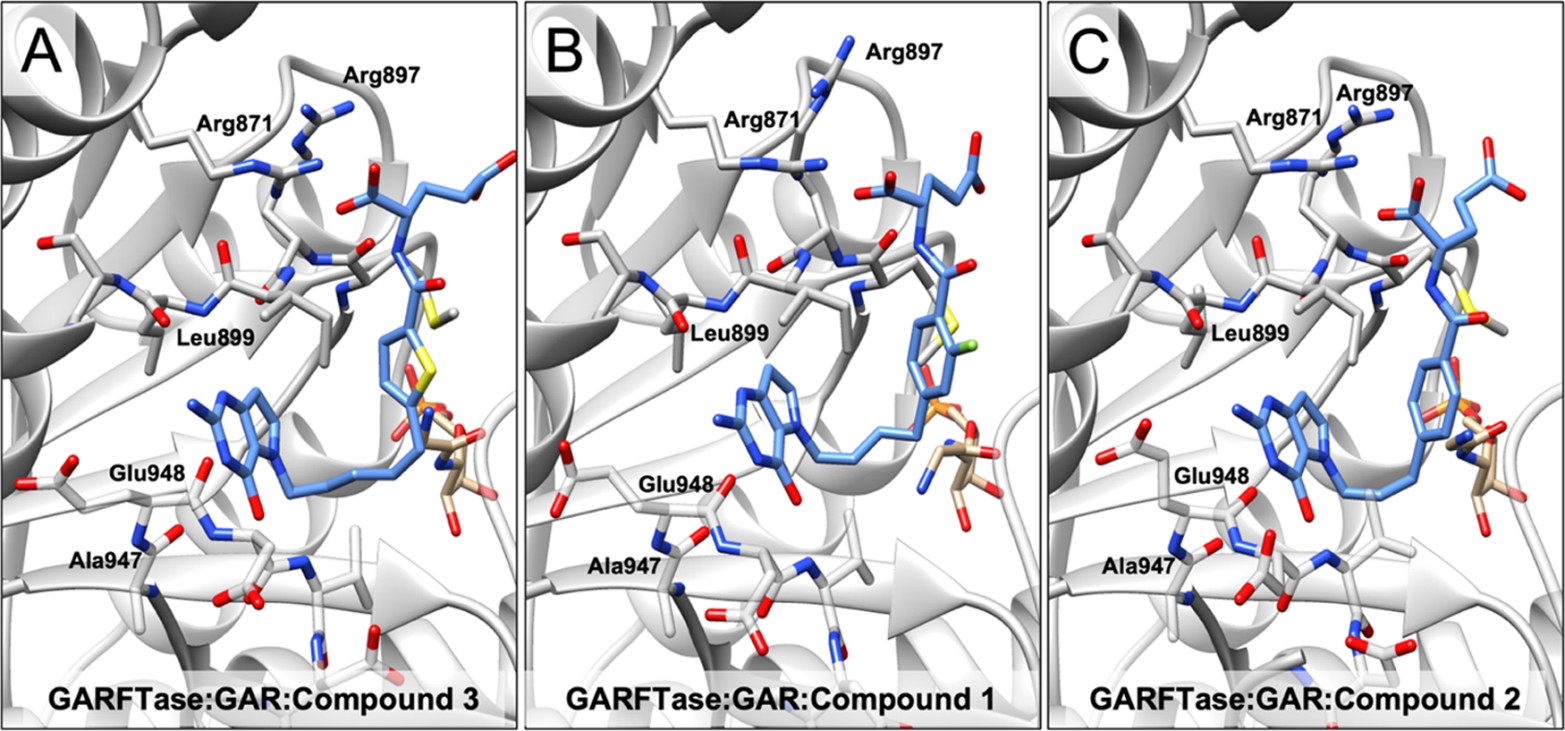
Crystal structures of inhibitors bound in the folate-binding pocket of GARFTase. Crystal structures were determined of GARFTase in complex with **3** (A, PDB ID: 8FJX), **1** (B, PDB ID: 8FJW), or **2** (C, PDB ID: 8FJY) (blue) and substrate GAR (tan). The protein is represented as a gray ribbon with interacting residues shown as sticks.

As our prior study showed that the 5-substituted pyrrolo[3,2-*d*]pyrimidine 5-atom bridge analogue **3** is a potent inhibitor of GARFTase (*K_i_* = 0.33 ± 0.22 μM),^36^ we solved the GARFTase crystal structure in complex with substrate β-GAR and **3**. We also solved the GARFTase crystal structures in complex with the related compounds **1** (4-atom bridge with intermediate inhibition) and **2** (3-atom bridge with no detectable inhibition up to 150 μM) for *in situ* modeling of the designed compounds in this study. GARFTase crystal structures in complex with **3**, **1**, and **2** were solved to resolutions of 2.17, 2.08, and 2.98 Å, respectively (Figure 9, PDB ID 8FJX, 8FJW, 8FJY, respectively). Data processing and refinement statistics (Table S2), as well as detailed interactions and maps for the complexes (Figures S3–S5), are provided in the Supplemental Information.

From the crystal structure of **3** in GARFTase, the fused pyrrolo[3,2-*d*]pyrimidine ring interacts with the backbone of Leu899, Glu948, and Asp949 through H-bonds, and the 7-CH interacts with the backbone carbonyl of Arg897 through an aromatic H-bond. The five-atom bridge resides in proximity to Asn913, His915, Lys923, Gly924, His944, Val946, and Val950 to make hydrophobic interactions. The α-COOH of the l-glutamate side chain of **3** makes two hydrogen-bond interactions with the protonated side chain of Arg871 and the backbone NH of Ile898. The amide-carbonyl oxygen forms a water-mediated hydrogen bond with the side chain and NH backbone of Ser925. Moreover, the α-COOH of **3** forms a salt bridge with the side chains of Arg871 and Arg897, while the γ-COOH remains solvent-exposed.

A comparison of the compound **3** GARFTase crystal structure with the crystal structures for GARFTase with **1** and **2** suggested that analogues with a 4- and 5-atom bridge show a preference in binding to the pocket over analogues with a 3-atom bridge. With **1** (4-atom bridge), the N1 makes a hydrogen bond with the backbone NH of Leu899, the 2-NH_2_ with the backbone NH of Ser900, and the N3 with the backbone carbonyl of Asp949 and Ala947. The 4-CO of **1** makes a hydrogen bond with the backbone NH of Asp951 and a water-mediated hydrogen bond with Asp940. The amide NH of the l-glutamate of **1** makes a hydrogen bond with Met896. The α-COOH of **1** forms a salt bridge with Lys844 and the γ-COOH forms multiple hydrogen bonds with water molecules. Although **1** and **3** inhibit GARFTase *in vitro* (at μM and nM concentrations, respectively), **2** with a 3-atom bridge linker did not inhibit GARFTase up to 150 μM.^36^ Evaluation of **2** with GARFTase showed that many of the polar contacts between the inhibitor and the enzyme binding pocket were substantially longer, to the point that some of these polar contacts do not likely contribute to binding. Specifically, most contacts shared in the **3** and **2** crystal structure complexes showed 0.2–0.8 Å longer contact distances in the **2** complex (e.g., polar contacts with backbone atoms of Met896, Leu899, Ala947, and Glu948, and with side-chain atoms of Arg871 and Arg897; *cf.* contact distances in Figures S4 and S5). Effectively, the 3-atom bridge in **2** is too short to simultaneously allow for optimal polar contacts with GARFTase at both the pyrrolo[3,2-*d*]pyrimidine and l-glutamate moieties.

Based on inhibition profiles and evaluation of our crystal structures, GARFTase complexed with **1** (PDB ID: 8FJW) was used for *in situ* modeling of the designed compounds (Figure 4) and docking studies. Compounds **1** (4-atom bridge), **7** (4-atom bridge with a sulfur heteroatom), **9** (5-atom bridge), **14** (4-atom bridge), and **15** (4-atom bridge) showed docked scores ranging from −16.0 to −14.4 kcal/mol (Table S1, Supplemental Information). In the docked pose of **15**, both the N1 and the 2-NH_2_ interact with Leu899; the 2-NH_2_ makes an additional interaction with Glu948, while the 4-oxo forms a water-mediated H-bond with Glu948. In the l-glutamate moiety, the α-COOH forms a network of hydrogen bonds with the backbone NH of Arg897 and the side chain of Arg871. The γ-COOH interacts with the side-chain NH groups of Arg897 and Lys844. The thiophene ring of **3** forms nonspecific hydrophobic interactions in the pocket. These interactions were unique from those in the solved GARFTase-compound **3** crystal structure. The remaining analogues showed similar docked poses and interactions to **3** in the GARFTase crystal structure.

#### Molecular Modeling with ATIC

ATIC is a bifunctional enzyme (AICARFTase/inosine monophosphate cyclohydrolase) in *de novo* purine biosynthesis, wherein the AICARFTase domain utilizes 10-formyl THF to convert AICAR to formyl-AICAR (Figure 1).^53^ The crystal structure of human ATIC in complex with the antifolate-based inhibitor *N*-[(*S*)-(4-{[(2-amino-4-hydroxyquinazolin-6-yl)dihydroxy-λ-4-sulfanyl]amino}phenyl) hydroxymethyl]-l-glutamic acid (BW1540U88UD) (PDB ID: 1P4R)^37^ was used for molecular docking studies. This structure was preferred over the crystal structure of ATIC bound to a nonclassical antifolate *N*-(6-fluoro-1-oxo-1,2-dihydroisoquinolin-7-yl)-5-[(3*R*)-3-hydroxypyrrolidin-1-yl]thiophene-2-sulfonamide (LSN 3213128) (PDB ID: 5UZ0)^54^ because of the ligand-structure similarity of the classical antifolate to our designed analogues.

The docked scores for the 4-atom bridge pyrrolo[3,2-*d*]pyrimidine compounds **14** (−14.4 kcal/mol), **16** (−13.9 kcal/mol), **1** (−12.9 kcal/mol), and **5** (−12.8 kcal/mol) suggested a preference for binding to ATIC over the 5-atom bridge compounds **11** (−11.0 kcal/mol), **9** (−11.1 kcal/mol), and **3** (−11.7 kcal/mol) (Table S1, Supplemental Information). This difference reflects the loss of interaction between the amino acids in the binding pocket and the l-glutamic acid moiety of the 5-atom bridge compounds (Figure S6, Supplemental Information). The thiophene ring in the 4-atom bridge in **14** and **16**, and the phenyl ring in **1** make π–π interactions with the side chain of Phe315 in ATIC. These π–π interactions with Phe315 were absent in the 5-atom bridge compounds as the bridge aromatic (phenyl/thiophene) ring is shifted away (closer to the solvent) in the bound conformation due to the increased bridge length. In the 4-atom bridge compound **15**, a π-cation interaction with the side chain of Lys358 is observed. A difference in the orientation of the l-glutamate between the 4- and 5-atom bridge compounds also impacts the interaction with the ATIC protein. The 3-atom bridge analogues (**2**, **4**, and **13**) showed differences in molecular docking including a lack of the π–π interaction with Phe315. In addition, due to a shorter bridge length, the l-glutamic acid does not actively interact with the amino acid side chains in the binding pocket. All of the additional analogues (with minor positional adjustments) maintained similar interactions of the pyrrolo[3,2-*d*]pyrimidine scaffold with the enzyme (Figure S6, Supplemental Information).

#### Molecular Modeling with Human Folate Receptors

To optimize the recognition and cellular uptake of our analogues by FRs, we used the X-ray crystal structure of human FRα in complex with our antifolate **10** [*N*-(4-{[2-(2-amino-4-oxo-4,7-dihydro-3*H*-pyrrolo[2,3-*d*]pyrimidin-6-yl)ethyl]amino} benzene-1-carbonyl)-l-glutamic acid] (PDB ID: 5IZQ).^38^ For human FRβ, we used the structure complexed with PMX (PDB ID: 4KN2).^39^ The docked scores of the proposed compounds in complex with FR α and β are summarized in Table S1 (Supplemental Information).

Compound **1** in the docked pose of human FRα binds in the folate-binding cleft (Figure S7, Supplemental Information). The 2-NH_2_ and 4-oxo moieties of **1** interact with amino acids in the binding pocket similar to the crystallized ligand **10**. The 2-NH_2_ and 3-NH groups interact with Ser147, and the 4-oxo forms a hydrogen bond with the side-chain NH of Arg103. The pyrrolo[3,2-*d*]pyrimidine scaffold is sandwiched between the side chains of Tyr85 and Trp171, similar to that seen with the pyrrolo[2,3-*d*]pyrimidine ring of **10** in its bound conformation. The l-glutamate moiety of **1** is oriented similar to the corresponding l-glutamate in **10**.^55^ The α-COOH of **1** forms a network of hydrogen bonds that involves the backbone NH of Gly137 and Trp138, and the side-chain NH of Trp140. The γ-COOH of **1** interacts with the side-chain NH moieties of Lys136 and Trp102 (not labeled), similar to that observed in the corresponding l-glutamate portion of **10** in the crystal structure with FRα.^55^ The ortho fluorine-substituted phenyl ring of **1** forms hydrophobic interactions with Trp102 and a π-cation interaction with protonated Arg103. The other 4-carbon-atom bridge analogues (**12**, **14**, **15**, and **16**) (Figure S7, Supplemental Information) showed similar interactions in the binding pocket and the best docked scores (−18.2 to −20.5 kcal/mol) (Table S1, Supplemental Information). The compounds with 4 bridge atoms including heteroatoms (**7**, **8**, and **5**) showed decreased binding, with docked scores ranging from −14.5 to −19.1 kcal/mol. Apart from **13** (−19.2 kcal/mol), the other 3-atom bridge analogues (**2** and **4**) showed few interactions in the binding pocket (docked scores of −18.8 and −17.4 kcal/mol, respectively).

To analyze binding to FRβ, the proposed analogues were docked in the X-ray crystal structure of FRβ bound to PMX (PDB 4KN2; Figure S8, Supplemental Information).^39^ The results predict that compounds with 3- to 4-carbon bridge compounds are preferred for FRβ uptake. Based on their docked scores (Table S1, Supplemental Information), **5**, **13**, and **15** are predicted to be among the top three compounds for FRβ transport. Compound **5** contains an oxygen heteroatom in the bridge adjacent to the phenyl ring that did not make any contacts in the binding pocket, nor did it interfere with the ability of the ligand to mimic the PMX-bound conformation.^39^ The 2-NH_2_ and 4-oxo moieties of **5** interact in a similar manner to the corresponding groups of the PMX ligand, with the 2-NH_2_ and 3-NH interacting with Ser147, and the 4-oxo forming hydrogen bonds with the side-chain NH of Arg119 and His151. The pyrrolo[3,2-*d*]pyrimidine scaffold is sandwiched between the side chains of Tyr101 and Trp187, similar to that seen with the pyrrolo[2,3-*d*]pyrimidine scaffold of PMX in its bound conformation. The l-glutamate moiety of **5** is oriented similar to that of PMX with FRβ.^39^ The α-COOH of **5** forms a network of hydrogen bonds that involve the backbone NH of Gly153 and a water-mediated H-bond with Asp155. The γ-COOH interacts with the side-chain NH groups of Arg152 and Gln116. The phenyl ring of **5** forms hydrophobic interactions with Trp150 and π–π interactions with Trp118 and Trp156. The hydrophobic 3-C part of the bridge of **5** forms nonspecific hydrophobic interactions in the binding pocket. The other analogues (**1**, **3**, **12**, and **14**) show similar interactions to **5**. Compound **3** with a 5-atom bridge makes appropriate adjustments in the linker conformation so as to retain the pyrrolo[3,2-*d*]pyrimidine and l-glutamic acid moieties in the same positions as in **5** and to preserve all of the molecular interactions with FRβ. The docked scores and figures of the docked poses of the proposed compounds are included in Table S1 and Figure S8 (Supplemental Information).

#### Summary of Drug Design

**Figure 10:**
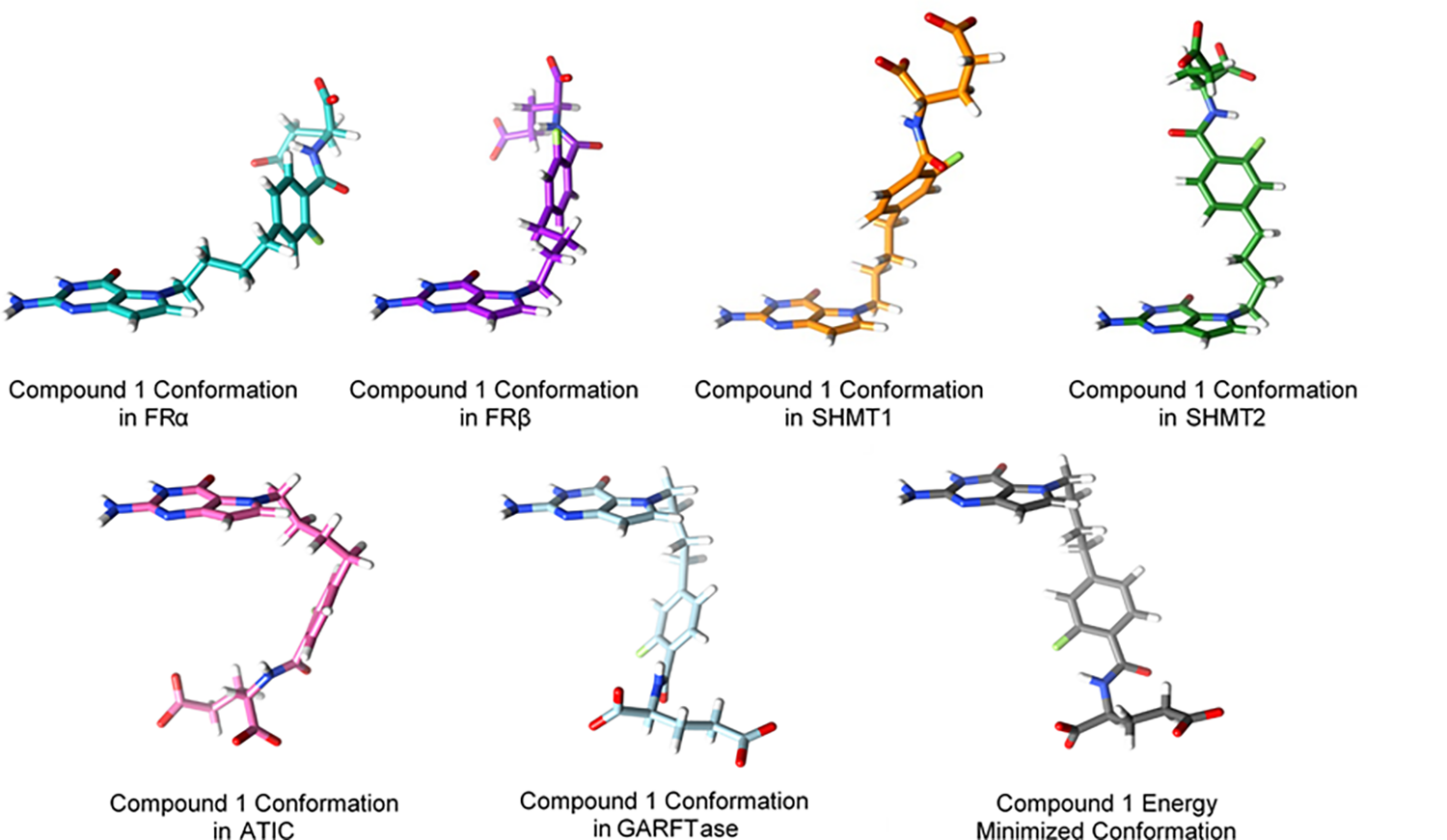
Docked conformations of **1** in FRα, FRβ, SHMT1, SHMT2, ATIC, and GARFTase compared to the energy-minimized (unbound) conformation of **1**.

Based on our determinations of the crystal structures and extensive molecular docking of our proposed compounds, we identified eleven 5-substituted pyrrolo[3,2-*d*]pyrimidine analogues with the potential to bind to FRs and critical enzyme targets (SHMT2, SHMT1, GARFTase, AICARFTase) in mitochondria and cytosol, resulting in potent and selective antitumor efficacies. These provide a compelling rationale for their synthesis and biological characterization. Not surprisingly, comparison of the docked poses of **1** with multiple protein targets showed that the inherent flexibility of our compounds is an essential design element required for the desired tumor-specific transport and multienzyme targeting (Figure 10).

### Chemistry

**Scheme 1:**
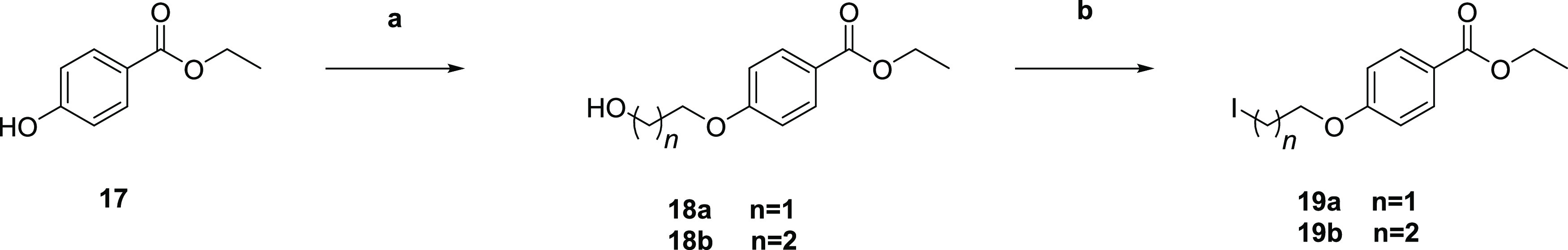


The intermediates (**18a** and **b**, Scheme 1) for compounds **5** and **6** were synthesized via an S_N_2-like attack on ethyl 4-hydroxybenzoate (**17**) following a modified version of the reported method.^56^ Acetonitrile was used instead of *N*,*N*-dimethylformamide (DMF) as a solvent to avoid evaporation at higher temp or the extensive workup procedure.^57^ Compounds **18a** and **18b** were converted to the mesylate derivatives and then to the respective iodides **19a** and **19b** using the Finkelstein reaction.

**Scheme 2:**
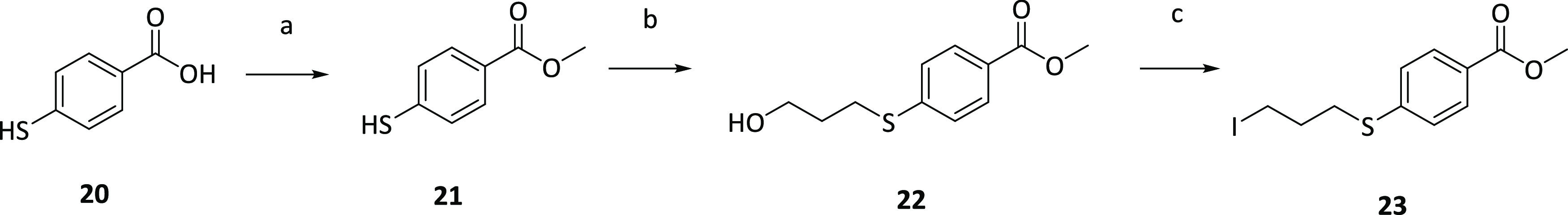


The synthesis of **7** commenced with the Fisher esterification of 4-mercaptobenzoic acid (**20**) to afford **21** (Scheme 2).^58^ The substituted thiophenol **22** was obtained via an S_N_2 attack of 4-mercaptobenzoate (**21**) and Cs_2_CO_3_ was used instead of K_2_CO_3_ as it is a better counterion for sulfur.^59^ The alcohols were converted into iodides, following a similar method to that described for **19a** and **19b** (Scheme 1).

**Scheme 3:**
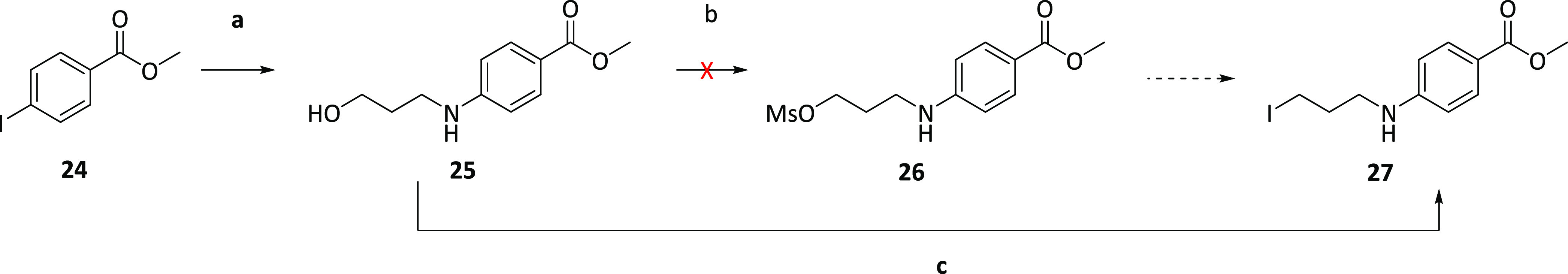


The intermediate for **8**, methyl 4-((3-hydroxypropyl)amino)benzoate (**25**, Scheme 3), was synthesized using methyl 4-iodobenzoate (**24**), CuI (as catalyst), l-proline, and 2-amino ethanol in dimethyl sulfoxide.^59,60^ Five equivalents of 2-amino ethanol were used to function as both base and nucleophile. The usual method to convert alcohols to iodides showed multiple products on thin-layer chromatography (TLC) analysis, and the yields were poor. Hence, a one-pot Appel reaction was used to obtain **27**.^56^ This afforded a much better yield.

**Scheme 4:**
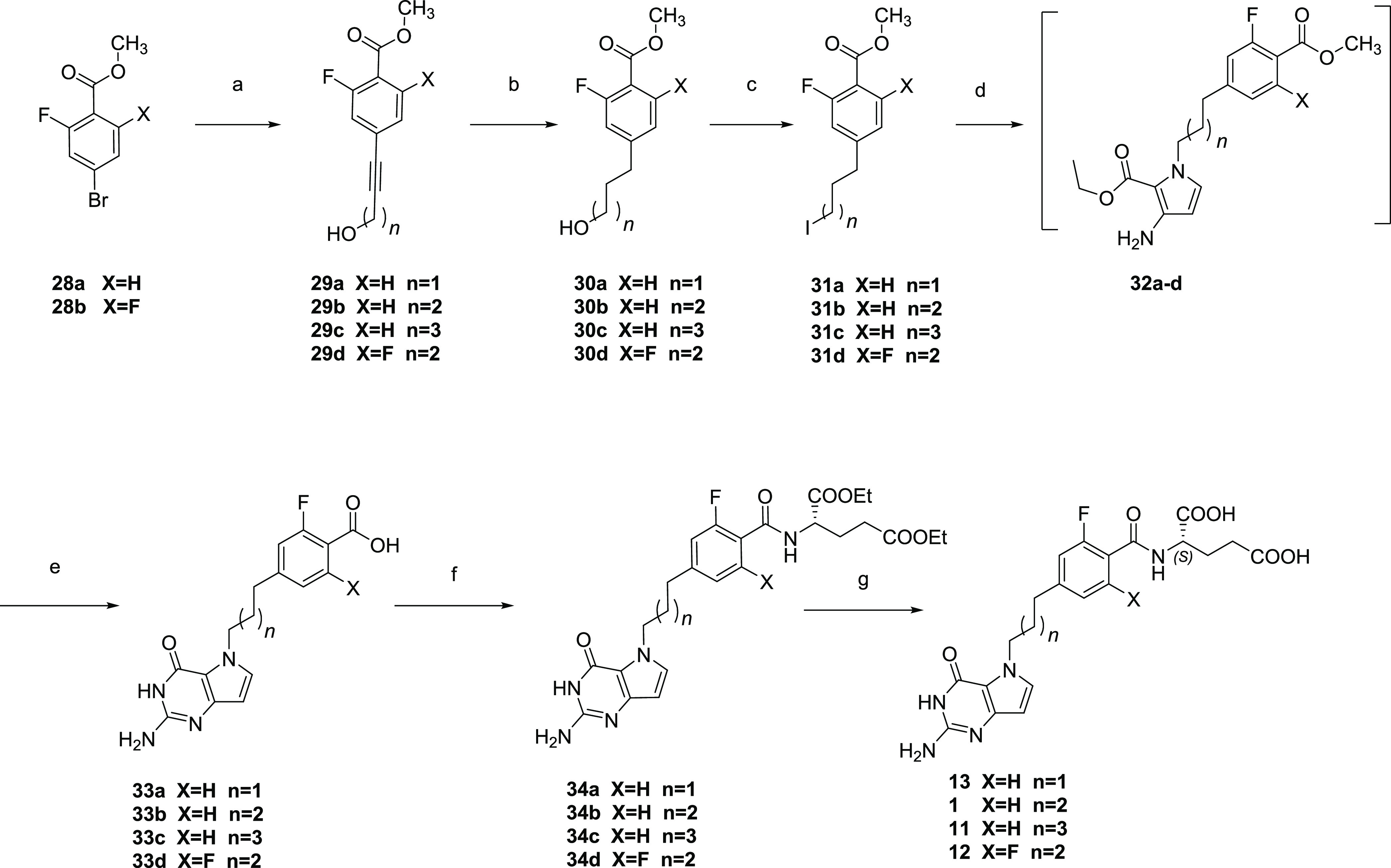


While syntheses of compounds **1** and **11** were published earlier,^36^ improved synthetic approaches are described in Scheme 4. Syntheses of **1**, **11**, **12**, and **13** (Scheme 4) started with a palladium-catalyzed Sonogashira coupling of **28a** and **b** with the appropriate alkyne alcohols to afford **29a**–**d**. Catalytic hydrogenation of **29a**–**d** afforded the saturated alcohols **30a**–**d**.^48^ Compounds **30a**–**d** were converted to their respective iodides **31a**–**d** using the Appel reaction as in Scheme 3. The *N-*alkylation using iodides **31a**–**d** afforded the *N*-5-substituted pyrroles **32a**–**d**. The crude *N*-substituted pyrroles **32a**–**d** were directly subjected to condensation with 1,3-bis(methoxycarbonyl)-2-methylthiopseudourea and subsequent saponification at 55–65 °C to afford the free pteroic acids **33a**–**d**.^61^ Peptide coupling of **33a**–**d** with l-glutamic acid diethyl esters using HATU as the coupling reagent gave **34a**–**d** and subsequent saponification afforded the target compounds **13**, **1**, **11**, and **12**, respectively. Previous coupling of the pteroic acids **33b** and **33c** with the l-glutamate ester for the synthesis of **1** and **11** utilized CDC as a coupling reagent and NMM as a base,^36^ affording **34b** and **34c** in 43 and 32% yields, respectively. With the modified method using HATU coupling and *N*,*N*-diisopropylethylamine (DIPEA) as a base, yields were improved for **34b** and **34c** to 65 and 69%, respectively.

**Scheme 5:**
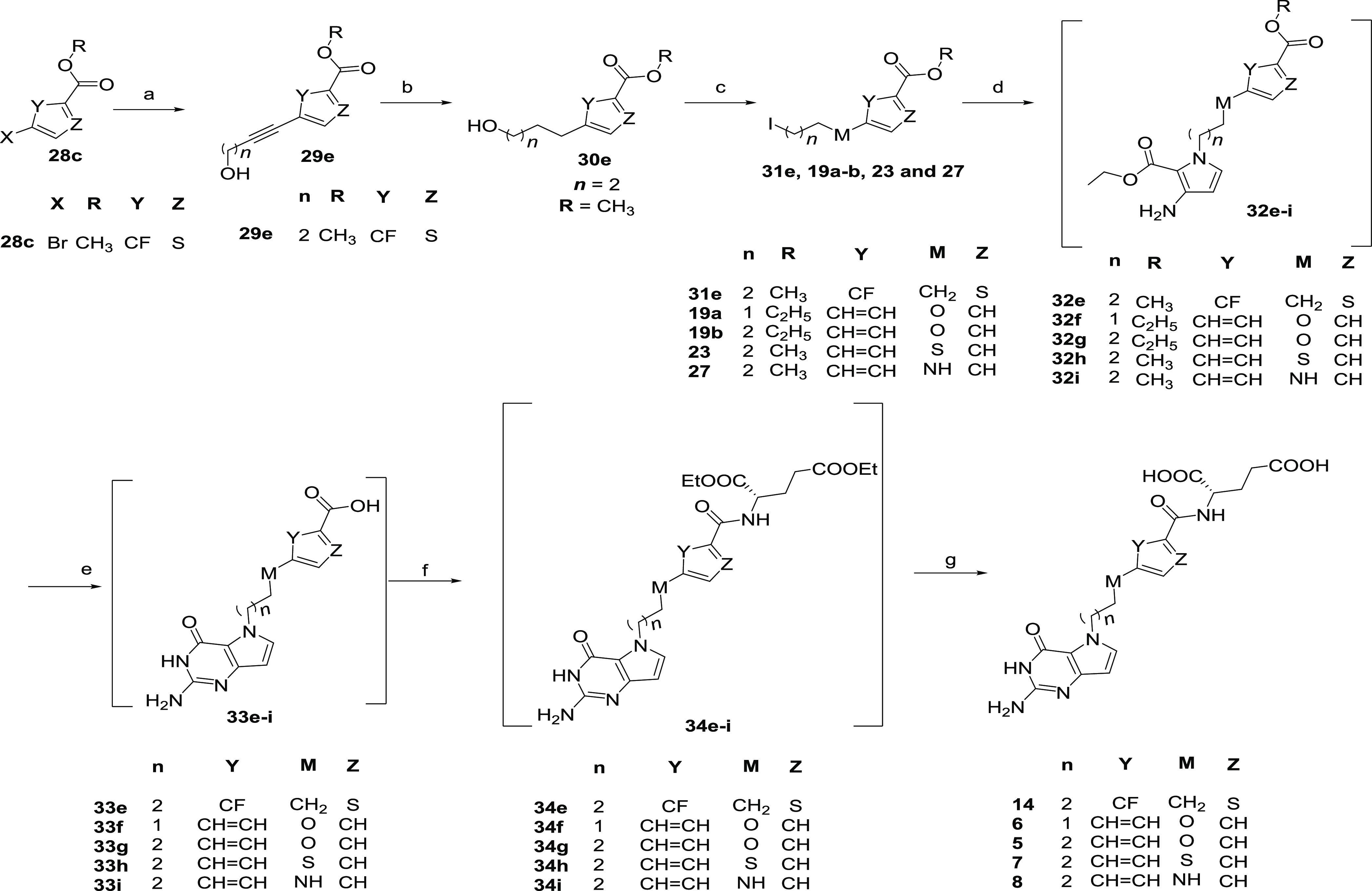


Compounds **14** and **5**–**8** were synthesized using the synthetic procedure described in Scheme 5. Following the palladium-catalyzed Sonogashira coupling of **28c** with 3-butyn-1-ol to **29e** and catalytic hydrogenation of **29e** resulted in the formation of **30e**. Further conversion of the alcohol of **30e** to the iodide (**31e**) used the same two-step method described in Scheme 1. *N-*Alkylation using iodide (**31e**) resulted in the crude *N*-substituted pyrrole (**32e**). **32e** was saponified using sodium hydroxide at 55 °C to afford the free pteroic acid **33e**.^61^ Peptide coupling of **33e** using CDMT as the coupling reagent with l-glutamic acid diethyl ester gave **34e**. Subsequent saponification of **34e** afforded the target compound **14**.

Compounds **6, 5, 7**, and **8** were synthesized using intermediates **19a/19b** (Scheme 1), **23** (Scheme 2), and **27** (Scheme 3), respectively, as in Scheme 5. Compounds **19a**, **19b**, **23**, and **27** were *N*-alkylated to form crude **32f**–**i**, which were cyclized using CDMT and hydrolyzed to form crude **33f**–**i.** These were coupled to l-glutamate to form crude **34f**–**i**. Finally, **34f**–**i** were saponified to form the final compounds (**5**–**8**).

### X-ray Crystal Structures of 5-Substituted Pyrrolo[3,2-*d*]Pyrimidine Antifolates with SHMT2 and GARFTase

**Figure 11:**
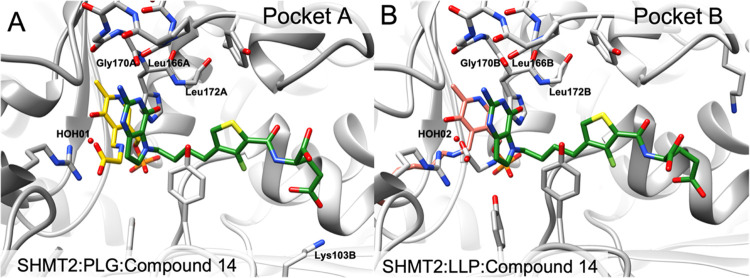
Structural analysis of 5-substituted pyrrolo[3,2-*d*]pyrimidine analogue **14** in complex with SHMT2-PLP-gly-antifolate ternary complexes. (A) Crystal structure of **14** bound in the THF binding pocket A of the asymmetric unit of the SHMT2 dimer including the natural cofactor PLP bound to glycine (PLG) (gold). (B) Crystal structure of **14** bound in the THF binding pocket B of the asymmetric unit of the SHMT2 dimer including the natural cofactor PLP bound to Lys280 (LLP) (salmon).

An SHMT2 crystal structure in complex with **14** was solved to a resolution of 2.47 Å (Figure 11; PDB ID: 8FJT). PLP-loaded SHMT2 was co-crystallized with **14** and serine. In pocket A of the **14** structure, an adduct of PLP bound to glycine (Figure 11A; PLG, shown in yellow) was modeled; in pocket B, PLP bound to Lys280 (Figure 11B; LLP, represented in salmon) was modeled. Detailed SHMT2: inhibitor contacts are provided in Figure S9 (Supplemental Information). Although **1** exhibits one conformation in pocket A and two conformations in pocket B (Figure 6), the electron density for **14** adopts only one conformation in each binding pocket. From the crystal structures, the bridge with the fluorothiophene scaffold in **14** adopts an orthogonal orientation to the pyrrolo[3,2-*d*]pyrimidine scaffold, mimicking that of **1**.

The pyrrolo[3,2-*d*]pyimidine scaffolds of **1** and **14** share conserved polar contacts with the backbone atoms of Gly170 and Leu172 in SHMT2 (*cf.*Figures 6, and 11A; S1 and S9, Supplemental Information). The fluorothiophene ring of **14** forms an additional π–π stacking interaction with the phenyl ring of Tyr105 (Figures 11B and S9, Supplemental Information). Compound **14** also coordinates a conserved water molecule with N1 of the pyrrolo[3,2-*d*]pyrimidine scaffold (Figure S9, Supplemental Information).

**Figure 12:**
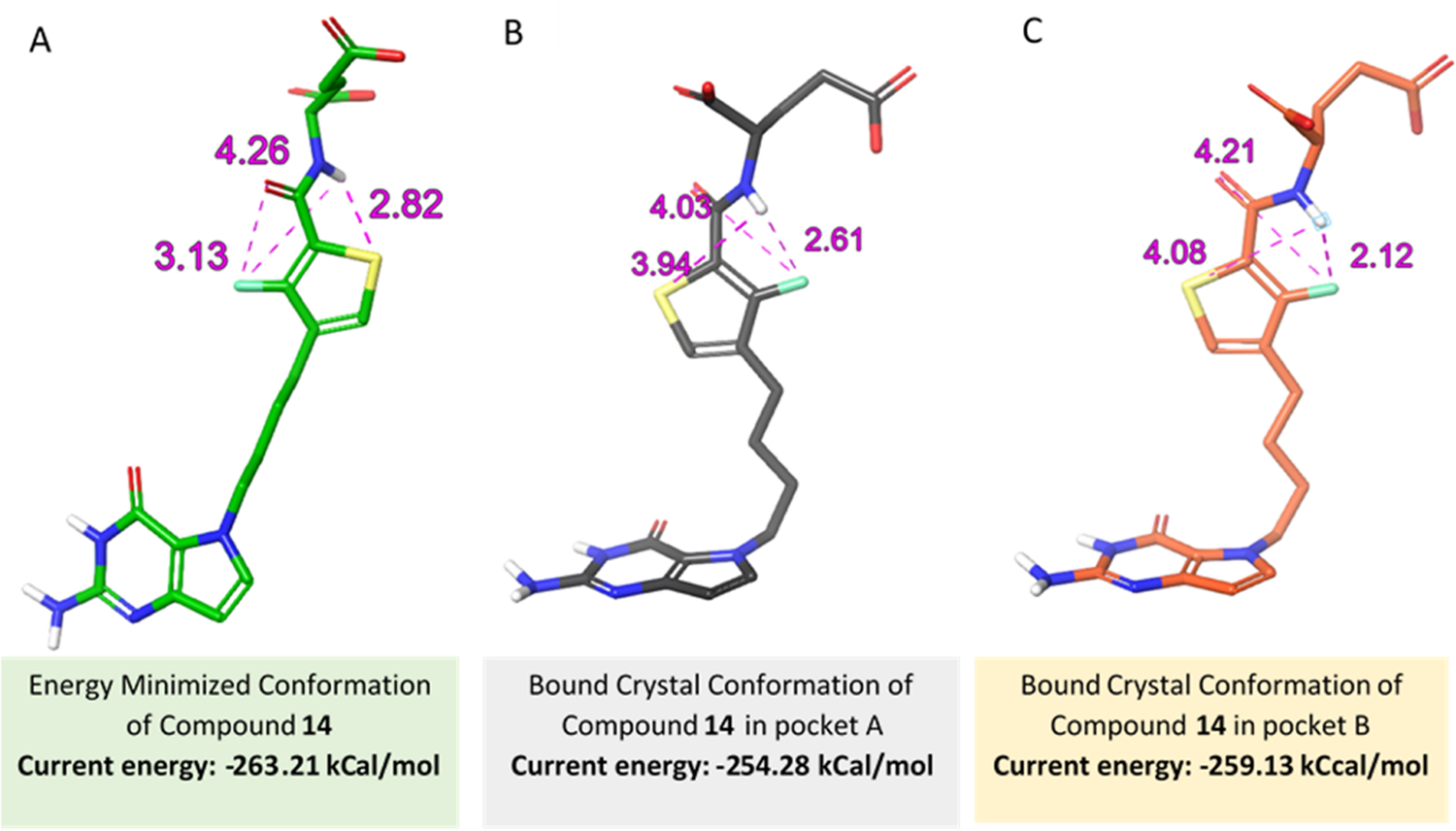
Comparison of **14** conformations in the molecular modeling and X-ray crystal structures for human SHMT2. (A) Energy-minimized structure of **14**. The distance between F–H–N is 4.26 Å, the distance between F–O is 3.13 Å, and the distance between S–H–N is 2.82 Å. (B) Ligand-bound crystal structure of **14** in SHMT2 pocket A with the distance between F–H–N of 2.61 Å; the distance between F–O is 4.03 Å; and the distance between S–H–N is 3.94 Å. (C) Ligand-bound crystal structure of **14** in SHMT2 pocket B with the distance between F–H–N of 2.12 Å; the distance between F–O is 4.21 Å; and the distance between S–H–N is 4.08 Å.

In one pocket (pocket A) of the SHMT2 binding site in complex with **14**, the binding of the l-glutamate tail in **14** is mediated by polar contacts with the side chain of Lys103 (O24 of the γ-COOH) via salt bridge formation, while the α-COOH has no polar contacts with the protein (Figure S9, Supplemental Information). In the other pocket (pocket B), both carboxylates remain solvent-exposed. For compound **14**, the hydrogen bond between the fluorine and the amide NH (N19) is observed in the SHMT2 bound conformation (Figure 12B,C). This interaction partially restricts the orientation of the l-glutamate tail in a specific conformation and is predicted to enhance the binding affinity. From molecular modeling, it was found that the energy-minimized conformation of **14** coordinates the fluorine and O18 of the amide-carbonyl on the same side of the aromatic ring (Figure 12A).

**Figure 13:**
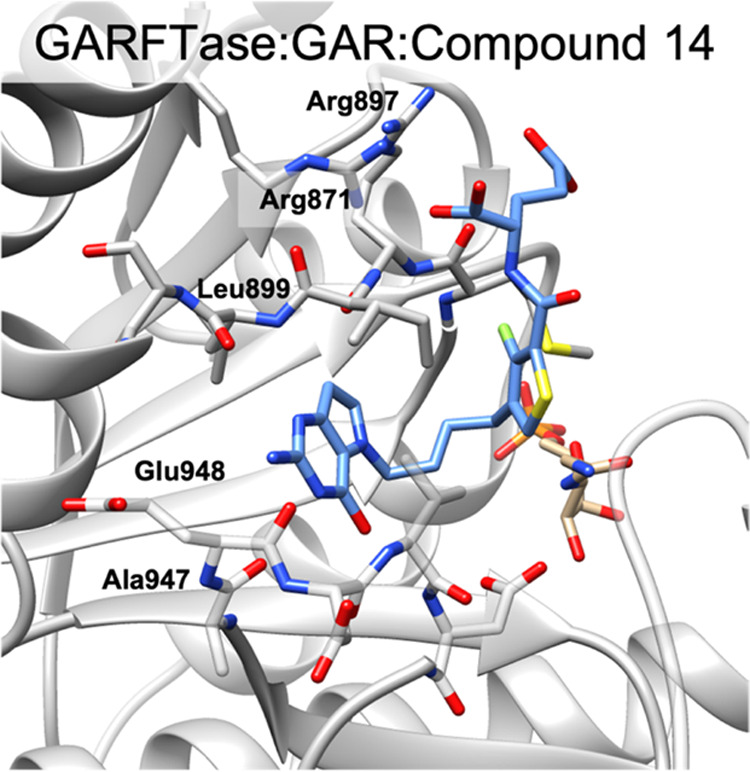
Structural analysis of 5-substituted pyrrolo[3,2-*d*]pyrimidine analogue **14** in complex with GARFTase and β-GAR. Crystal structure of **14** bound in the 10-formyl THF binding pocket of GARFTase including the natural cofactor β-GAR (tan).

For comparison to the initial GARFTase crystal structures of **1, 3**, and **2** (Figures 9; S3–S5, Supplemental Information), we determined the structure of GARFTase in complex with the newly designed analogue **14** (Figures 13 and S10, Supplemental Information). For all compounds, the pyrrolo[3,2-*d*]pyrimidine scaffold shares the same polar contacts with the backbone atoms of Leu899 and Glu948 in GARFTase. The N3 of the scaffold of **3** forms an additional polar contact with the backbone atoms of Ala947 (Figures 9B and S4). Compounds **1** and **14** coordinate a water molecule with N3 and O10 of the pyrrolo[3,2-*d*]pyrimidine scaffold (Figures 9C, 13, S3, and S10, Supplemental Information). In addition, **3** coordinates a water molecule with O10 of the pyrrolo[3,2-*d*]pyrimidine scaffold and the N3 of **3** makes a backbone contact with Ala947 (Figure S4). The binding of the l-glutamate is mediated by conserved contacts with the side chains of Arg871 and Arg897, as well as the backbone atoms of Ile898 and Met896 (seen in all structures). From this set, only **14** shows contact between the side chain of Arg897 and the γ-COOH (O25), rather than the α-COOH as observed with the **1**, **2**, and **3** structures.

### Biological Evaluation

#### *In Vitro* Validation of Intracellular Targets with Isolated C1 Metabolic Enzymes

We measured inhibition kinetics of the pyrrolo[3,2-*d*]pyrimidine antifolates **5**–**9** and **11**–**16** compared to compounds **1**–**4** from our previous report,^36^ with purified enzyme preparations of SHMT1, SHMT2, GARFTase, and ATIC to calculate inhibition dissociation constants (*K_i_*s) (Table 2). As expected, the compounds inhibited both SHMT2 and SHMT1 with affinities spanning ranges of 1331- and 304-fold, respectively. The potencies for the top (**11** and **9**, respectively) and bottom (**8**) compounds (Table 2) for each target paralleled the calculated docking scores (Table S1, Supplemental Information). The overall rank order for inhibition of SHMT2 [**11** > **3**–**9** > **2** > **1**–**13**–**14**–**5** > **16** > **4** > **15**–**12** > **7** > **6** > **8**] and SHMT1 [**9** > **3** > **11** > **1**–**13** > **2**–**15**–**16**–**14** > **5** > **12** > **7** > **4** > **6** > **8**] was highly congruous.

**Table 2:**
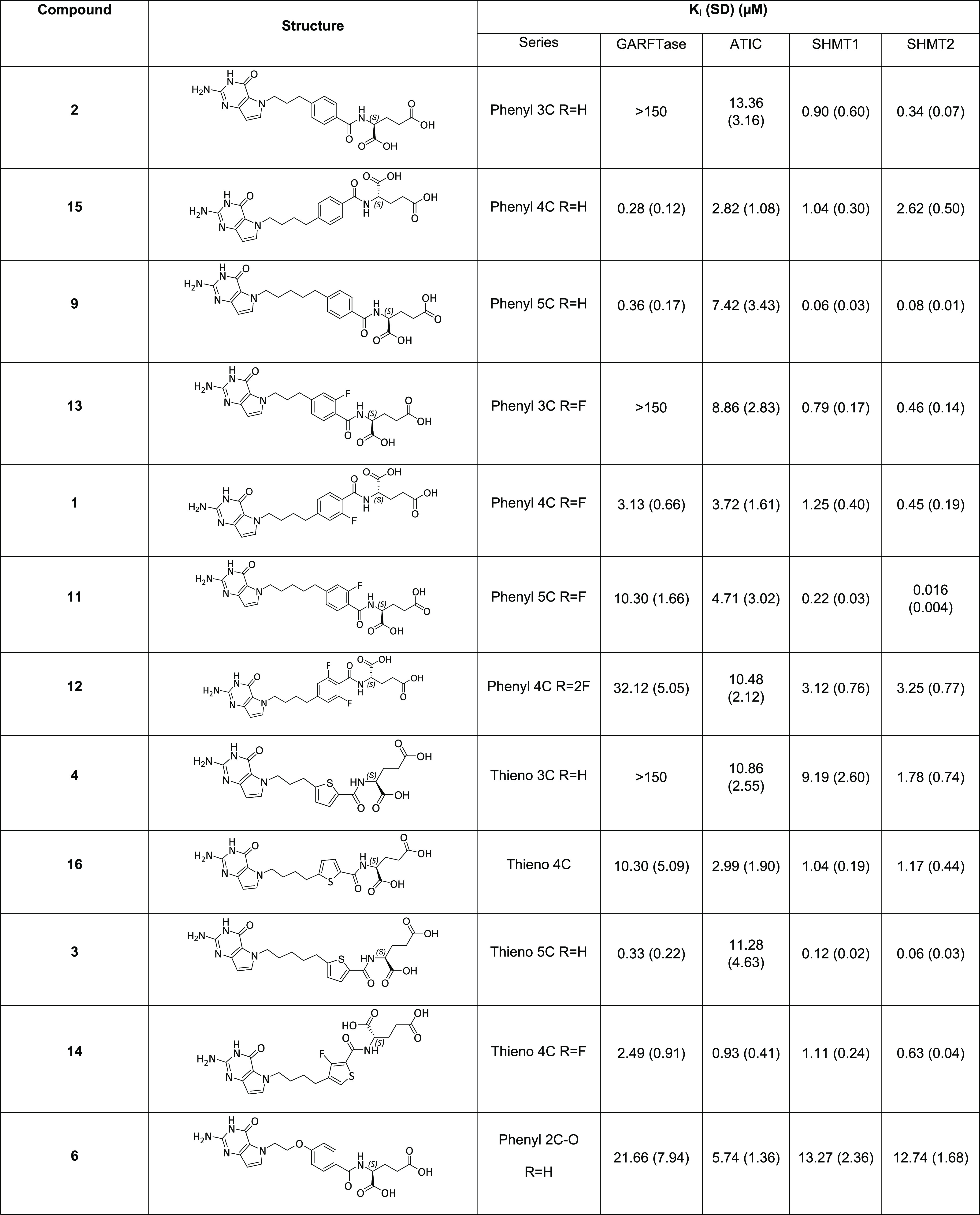

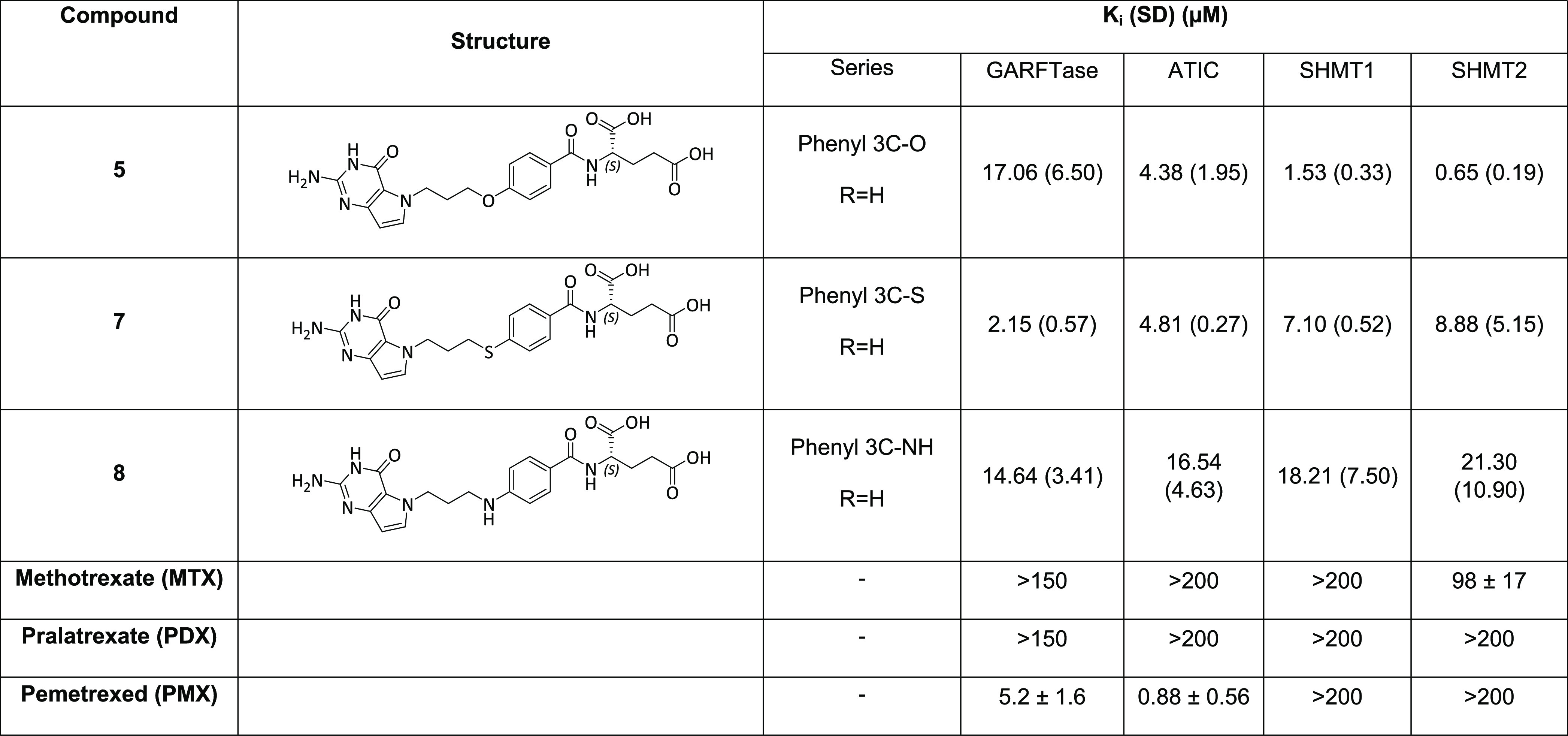
*K_i_*’s for Inhibition of Enzymes for 5-Substituted Pyrrolo[3,2-*d*]pyrimidine Antifolates^a^

a For the *in vitro* enzyme assays, *K_i_* values are presented as mean values (±standard deviations) from at least 3 replicate experiments. Methods are described in the Experimental Section.

For both SHMT1 and SHMT2 inhibition, compounds with a 5-atom bridge were preferred, although the bridge aromatic moiety was also a factor. Against SHMT2, **11** (5 atom, 2F phenyl bridge) showed a 28-fold increased potency over lead analogue **1** (4-atom, 2F phenyl bridge). This was followed by **3** (5-carbon, thiophene bridge) and **9** (5-carbon bridge, phenyl bridge) with a 7.5- and 5.6-fold increase in potency, respectively, over **1**. A 2-fluoro-phenyl bridge (**11**) increased the inhibition potency by 2-fold over a phenyl bridge (**9**), although replacement of the bridge phenyl with a 2,5-thiophene (*cf.***3** and **9**) had no impact on the inhibitory activity.

A slightly different inhibition profile emerged with SHMT1, for which the most potent inhibitor was **9** (21-fold more potent than **1**), followed by **3** (10.4-fold more potent than **1**) and **11** (5.68-fold more potent than **1**). Insertion of heteroatoms (O, S, or N) in the 4-atom bridge (i.e., **5**, **7**, or **8**) had varied effects. Oxygen (**5**) afforded similar inhibition of SHMT1 and SHMT2 to the carbon isostere **15**. Sulfur (**7**) and nitrogen (**8**) were generally detrimental to inhibition of both SHMT1 and SHMT2 compared to **15**. Insertion of oxygen in the 3-C bridge (**6**) was also detrimental to the inhibition of both SHMT1 and SHMT2. Although replacement of a phenyl (**2**) in the 3-carbon bridge series with a thiophene (**4**) decreased the potency for both SHMT2 and SHMT1, this did not extend to the 4-carbon (**15** and **16**) and 5-carbon (**9** and **3**) bridge compounds for which the activities were similar. Likewise, the impact of aromatic fluorine substitutions (**13**, **1**, **11**, **14**) on inhibitory potencies compared to the nonfluorinated counterparts (**2**, **15**, **9**, and **16**) was nominal.

For GARFTase, compounds **2**, **4**, and **13** (all 3-carbon bridge, phenyl, thiophene, or 2F phenyl, respectively) were not inhibitory up to 150 μM. The remaining compounds inhibited GARFTase over an ∼115-fold range with *K_i_* values from 0.28 to 32.1 μM. The most potent compounds were **3** (5-carbon, thiophene bridge), **9** (5-carbon, phenyl bridge), and **15** (4-carbon, phenyl bridge) with *K_i_* values of ∼0.3 μM (up to 10-fold better than **1**). Although compounds **7** (4-atom bridge, S insertion) and **14** (4-carbon, F-thiophene bridge) had similar potencies to **1** (4-carbon, 2F phenyl bridge) toward GARFTase, the remaining compounds (**5**, **6**, **8**, **11**, **12**, and **16**) were less potent than **1**. The *K_i_* values for GARFTase were only partly reflected by docked scores (Tables 2 and S1, Supplemental Information).

For ATIC, *K_i_* values spanned an ∼18-fold range with the most potent inhibitor **14** (4-carbon, thiophene bridge) showing a *K_i_* of 0.93 μM (4-fold better than **1**). However, the differences in inhibitory potencies for the remaining compounds were modest with no particularly significant structural motif. The three compounds with the best *K_i_* values (**14**, **15**, and **16**) for ATIC (Table 2) all showed the best docked scores (Table S1, Supplemental Information).

Taken together, the *in vitro* enzyme inhibition assays further confirm multitargeting of SHMT1, SHMT2, GARFTase, and ATIC and establish a robust SAR for the pyrrolo[3,2-*d*]pyrimidine compounds. We also tested the compounds as inhibitors of MTHFD2; these were all inactive up to 200 μM.

**Figure 14:**
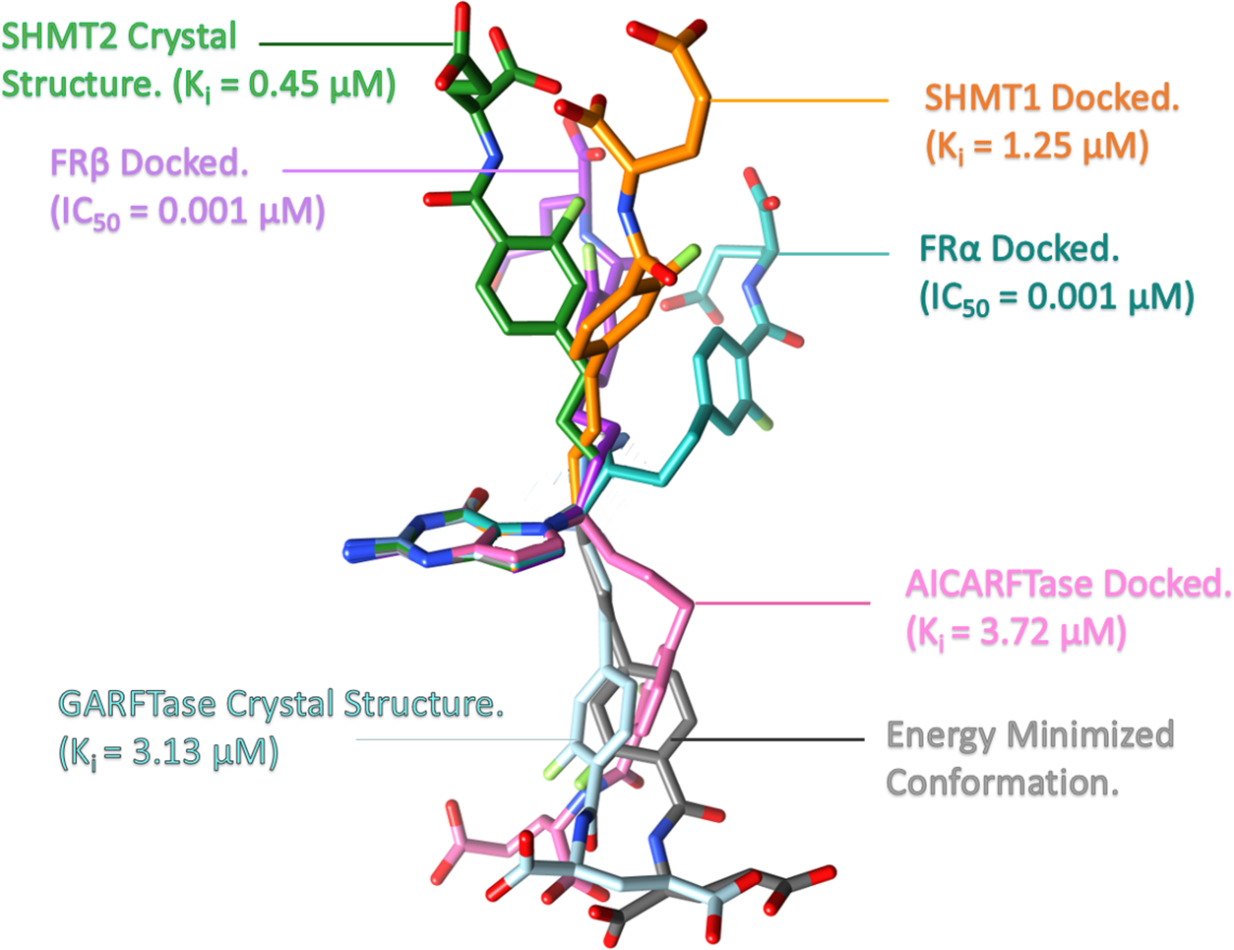
For visual comparisons, the bicyclic pyrrolo[3,2-*d*]pyrimidine ring system was superimposed for **1** in each binding pose, highlighting the conformational flexibility inherent in this compound to allow for multitargeting. Inhibitory activities from enzyme assays (SHMT1, SHMT2, GARFTase, AICARFTase; Table 2) and cell-based inhibition (FRα and FRβ; Table 3) are included.

The design premise of providing conformational flexibility to allow attachment to multiple targets was born out in the attachment of single agents to different target proteins in quite different conformations made possible by the inherent flexibility of the molecule. This flexibility is illustrated in Figure 14 with compound **1** as an example with the bound crystal structures or docked structure conformation along with the inhibitory potency.

When dealing with multitargeted single-agent inhibitors, it is unlikely that a particular analogue will have the best activity in all targets. Since the intracellular targets SHMT2, SHMT1, GARFTase, and ATIC have different binding sites and hence have different structural requirements including conformations for optimal binding as illustrated in Figure 14, a single analogue may not have the best inhibitory potencies toward all four target enzymes. Accordingly, the analogue with the best balance of inhibitory potencies in all four targets would be considered the most promising. In our series, **9** would be the most promising compound.

#### Antiproliferative Effects and Antitumor Activities of 5-Substituted Pyrrolo[3,2-*d*]pyrimidine Analogues

We measured the antiproliferative activities of the 11 novel pyrrolo[3,2-*d*]pyrimidine compounds compared to **1**, **2**, **3**, and **4** from our prior study^36^ in relation to the principal mechanisms of (anti)folate transport including the relative contributions of RFC, PCFT, FRα, and FRβ. We initially tested the compounds against a panel of isogenic Chinese hamster ovary (CHO) cell lines engineered from transporter (RFC/FR/PCFT)-null MTXRIIOua^R^2–4 (R2) cells,^62^ to express human RFC (PC43-10 cells), PCFT (R2/PCFT4 cells), FRα (RT16 cells), or FRβ (D4 cells).^63−65^ RFC, PCFT, and FR transport characteristics for these engineered CHO cell line models have been previously documented.^63−65^ By this assay, growth inhibition compared to R2 cells is a direct reflection of transport activity.^63,64^

The experimental design involved incubations of the CHO cell lines with the pyrrolo[3,2-*d*]pyrimidine compounds over a range of concentrations for 96 h, after which cell numbers were quantified with a fluorescence-based metabolic assay.^63^ The results are summarized in Table 3. By this assay, none of the analogues impacted the proliferation of transporter-null R2 cells up to 1000 nM. This establishes an absolute requirement for transport for drug activity. For RFC-expressing PC43-10 cells, **1**, **2**, and **13** showed inhibition (IC_50_ ∼ 42–200 nM), confirming RFC transport, whereas the additional 12 compounds were inactive up to 1000 nM, indicating a lack of RFC transport. Toward R2/PCFT4 cells, growth inhibition was in rank order, **13** > **2** > **1**–**4**–**6**, with the remaining compounds showing no inhibition (Table 3).

**Table 3:**
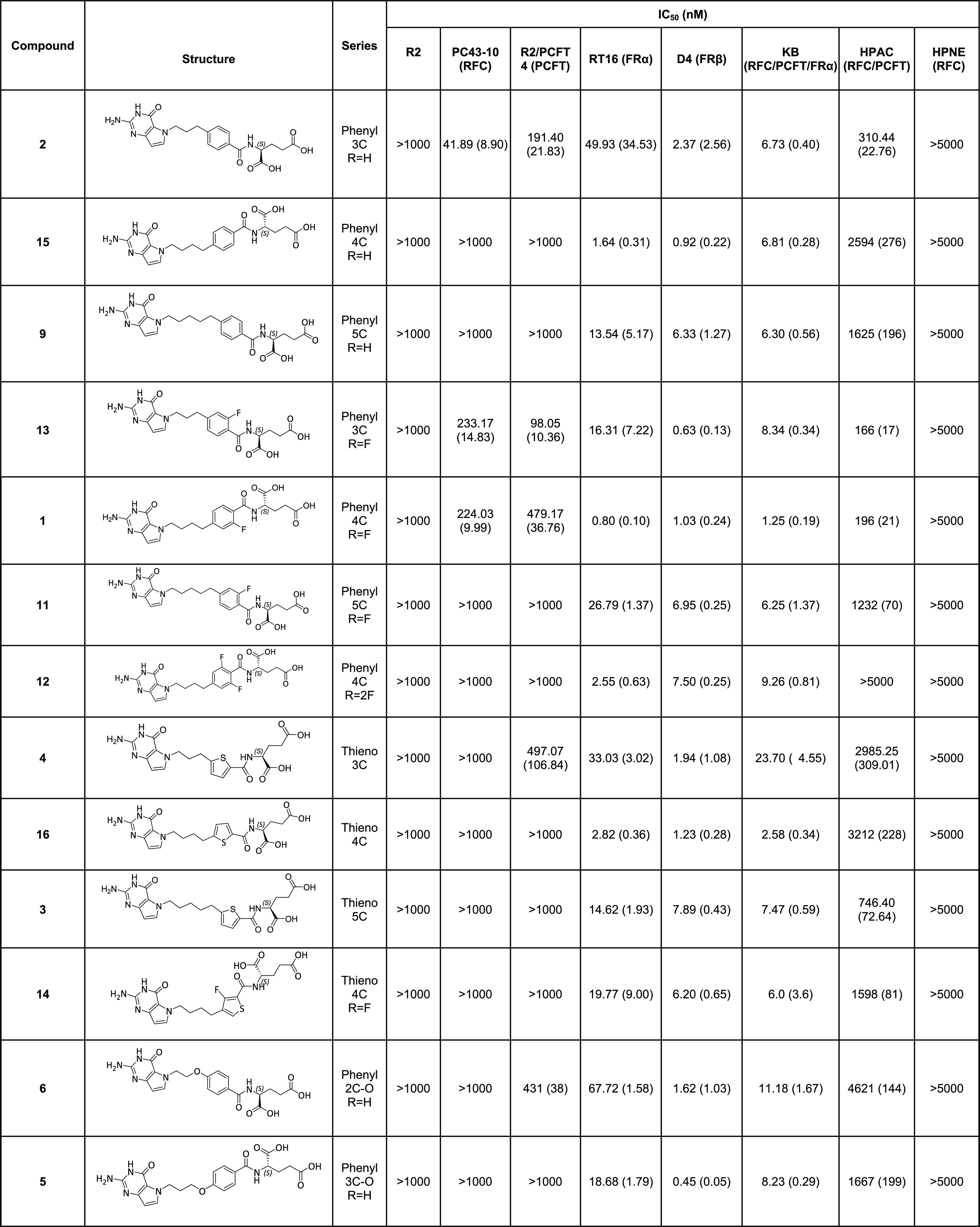

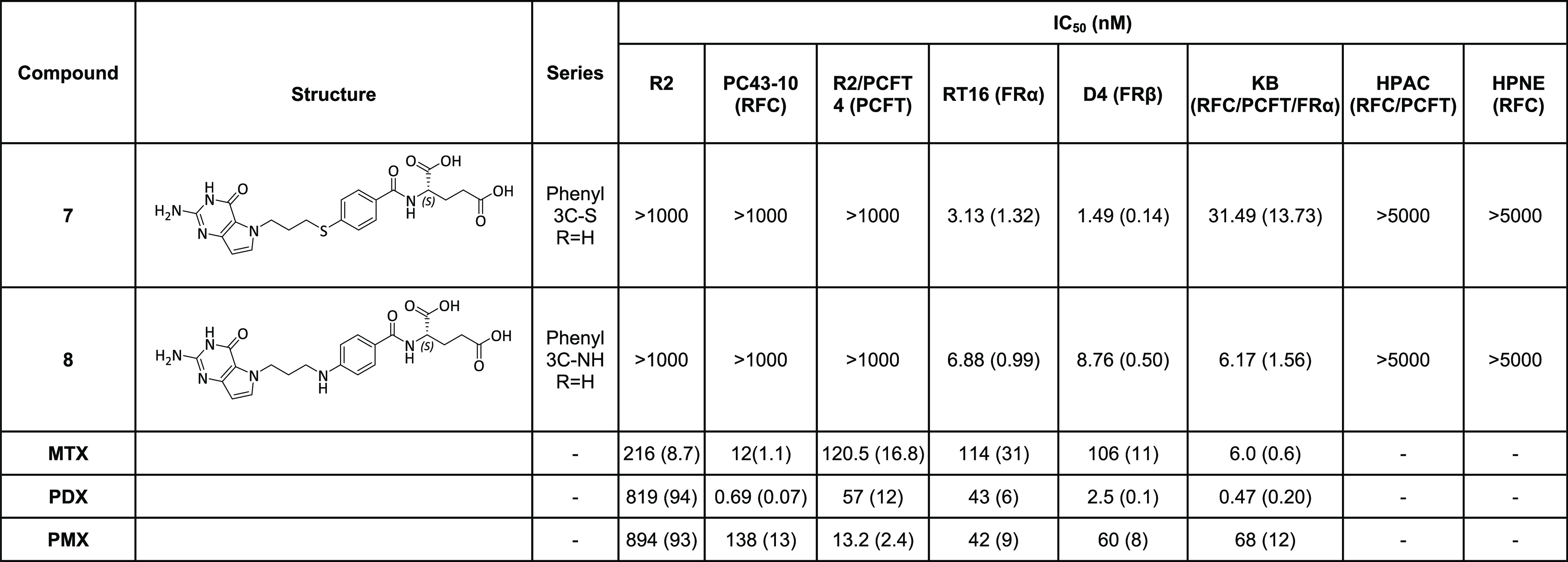
IC_50_’s for Inhibition of Proliferation of Isogeneic Chinese Hamster Ovary Cells and Human Tumor Cells by 5-Substituted Pyrrolo[3,2-*d*]pyrimidine Antifolates^a^

a Growth inhibition assays were performed for CHO sublines engineered to express human RFC (PC43-10), FRα (RT16), FRβ (D4), or PCFT (R2/PCFT4), for comparison with transporter-null (R2) CHO cells. Inhibition assays were also performed in KB (nasopharyngeal carcinoma; express RFC, FRα, and PCFT) and HPAC (pancreatic adenocarcinoma; express RFC and PCFT) cells and HPNE cells (human pancreatic normal epithelial cells; express RFC). For the RT16, D4, and KB cells, the experiments were performed in folate-free RPMI 1640 with dialyzed fetal bovine serum and antibiotics with 2 nM leucovorin;^63^ for the PC43-10 and R2/PCFT4 CHO experiments, 25 nM leucovorin was provided.^63,64^ For the RT16 and D4 cells, growth inhibition assays were performed in the presence and the absence of 200 nM folic acid (the latter is not shown). Proliferation inhibition assays were performed using the HPAC pancreatic adenocarcinoma cells and HPNE (human normal pancreatic epithelial) cell lines in folate- and glycine-free RPMI supplemented with 10% dialyzed fetal bovine serum and antibiotics, and 25 nM leucovorin.^5^ The data shown are mean values from 3–10 experiments (±standard errors in parentheses). Results are presented as IC_50_ values, corresponding to the concentrations that inhibit growth by 50% relative to cells incubated without drug. CHO cell line data for MTX, PDX, and PMX were previously published.^48,63−65,68,69^

All analogues of the series were inhibitory toward FR-expressing RT16 (FRα) and D4 (FRβ) CHO cells, differing within ∼2-fold in FR levels (measured by [^3^H]folic acid binding)^63^ (Table 3). For 9 compounds, the IC_50_’s for D4 (FRβ) were generally greater than for RT16 (FRα); with 5 compounds (**2**, **4**, **5**, **6**, and **13**), this difference was particularly notable (ca. 20- to 40-fold). Inhibition of RT16 and D4 cells was ablated by excess (200 nM) folic acid (not shown), consistent with uptake by FRs.^63−65^ Four compounds (**1**, **7**, **15**, and **16**) showed consistent high-level inhibitory activity (IC_50_ < 3 nM) toward both FRα and FRβ-expressing cells (Table 3).

Results for the 5-substituted pyrrolo[3,2-*d*]pyrimidine compounds were also compared to the classic antifolates MTX, PMX, and PDX (Table 3). Based on the CHO transport readout, the pyrrolo[3,2-*d*]pyrimdine analogues were much more selective than the classic antifolates for uptake by FRs (RT16, D4) over RFC (PC43-10). For the 5 compounds that showed PCFT-targeting (**1**, **2**, **4**, **6**, and **13**), selectivity over RFC was limited.

**Figure 15:**
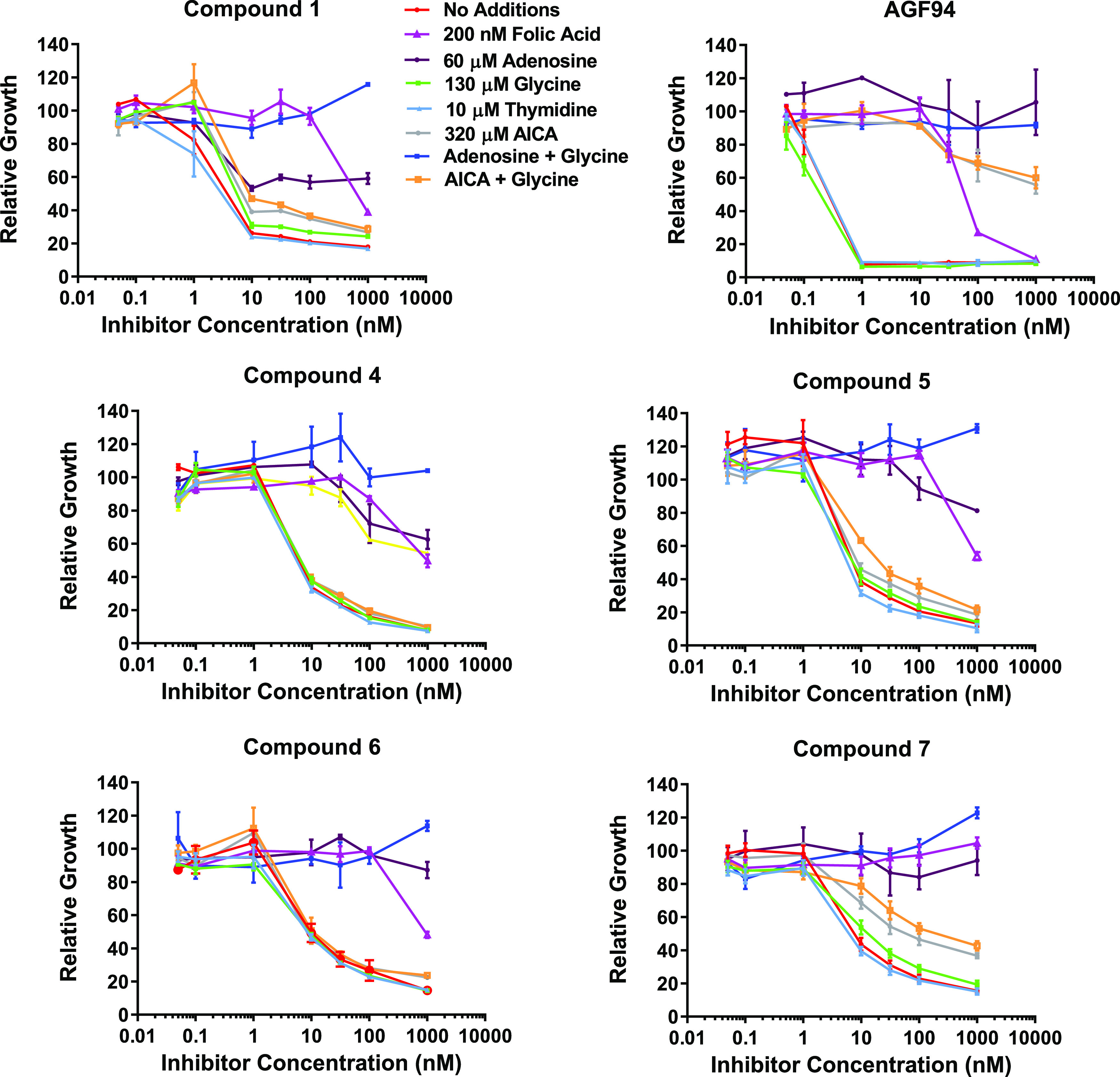
*In vitro* antitumor efficacy and identification of targeted pathways and enzymes by novel pyrrolo[3,2-*d*]pyrimidine analogues **4**–**7** in KB tumor cells compared to **1** and **AGF94**. Dose-response growth inhibition curves are shown for 6-substituted pyrrolo[3,2-*d*]pyrimidine antifolates compared to the established SHMT2 inhibitor **1**(36) and **AGF94**(69) for KB cells cultured in complete folate-, serine-, and glycine-free RPMI 1640 with 2 nM leucovorin, without additions, or in the presence of folic acid (200 nM) adenosine (60 μM), thymidine (10 μM), glycine (130 μM), and/or AICA (320 μM). The results shown are mean values ± standard deviations for three biological replicates. Results for compounds **8**, **9**, and **11**–**16** are shown in Figure S11 (Supplemental Information).

We compared the *in vitro* potencies of the 11 novel pyrrolo[3,2-*d*]pyrimidine compounds to compounds **1**–**4** against KB nasopharyngeal carcinoma cells, characterized by the expression of RFC, PCFT, and FRα^63,64,66^ (Table 3 and Figures 15 and S11, Supplemental Information), and HPAC pancreatic adenocarcinoma cells which express RFC and PCFT but not FRs (Table 3).^21^ Toward KB cells, all compounds were potently inhibitory within a narrow range, from 1.3 nM for **1** to 31. 5 nM for **7**. In the presence of 200 nM folic acid, the inhibitory effects were again abolished, although at higher concentrations for some of the analogues (e.g., **1**) folic acid was less effective (Figures 15 and S11, Supplemental Information). While this likely reflects uptake by other uptake systems including PCFT, the association between uptake by FRα (i.e., inhibition of RT16 cells) or PCFT (inhibition of R2/PCFT4; Table 3) and KB sensitivity or resistance was inexact. Toward HPAC cells, the potency of compound **13** was similar to that for compound **1** (IC_50_ values of 166 and 196 nM, respectively), followed by compounds **2** (310 nM) and **3** (746 nM), and compounds **5**, **9**, **11**, and **14** (ca. 1200–1600 nM). The remaining compounds showed modest inhibition (**4**, **6, 13**, **16**; IC_50_’s from ∼2600 to 4600 nM) or no activity (**7**, **8**, **12**; >5000 nM) toward HPAC cells. All of the pyrrolo[3,2-*d*]pyrimidine compounds were completely inert toward human pancreatic normal epithelial (HPNE) cells,^67^ demonstrating tumor cell selectivity over normal cells.

#### Identification of the Targeted Pathway(s) in KB Tumor Cells by Metabolite Rescue

To confirm the targeted pathways for the pyrrolo[3,2-*d*]pyrimidine antifolates in tumor cells, we treated KB human tumor cells cultured in serine- and glycine-free media with the pyrrolo[3,2-*d*]pyrimidine compounds (**4**–**9**, **11**–**16**) to test for the rescue by added nucleosides and glycine. Results were compared to those for **AGF94**, a pyrrolo[2,3-*d*]pyrimidine antifolate that inhibits GARFTase without effects on SHMT2,^69^ and to compounds **1**, **2**, and **3**, documented inhibitors of mitochondrial C1 metabolism and *de novo* purine biosynthesis in human tumor cells.^36^ The results are shown in Figures 15 and S11 (Supplemental Information). Inhibition by all of the compounds was unaffected by thymidine alone (10 μM), and there was no impact on cell proliferation of added thymidine (not shown) above the incomplete protection seen with added adenosine (60 μM) alone. In contrast, inhibition by **AGF94** was completely reversed by adenosine. Although glycine (130 μM) alone had no effect on growth inhibition, glycine combined with adenosine was completely protective for all of the pyrrolo[3,2-*d*]pyrimdine compounds in Table 3. This pattern of metabolite protection is diagnostic of mitochondrial C1 inhibitors.^36^ 5-Aminoimidazole-4-carboxamide (AICA) was partly protective from growth inhibition without or with glycine, reflecting inhibition of AICARFTase, the 2nd folate-dependent step in purine biosynthesis.^13,36,64^

These results suggest that pyrrolo[3,2-*d*]pyrimidine compounds **5**–**9** and **11**–**16**, like **1**, **2**, and **3** previously,^36^ are inhibitors of mitochondrial C1 metabolism along with *de novo* purine biosynthesis in the cytosol, results entirely consistent with the *in vitro* studies with isolated C1 enzymes (Table 2).

## Conclusions

We previously described multitargeted 5-substituted pyrrolo[3,2-*d*]pyrimidine antifolates **1**, **2**, **3**, and **4**.^36^ The lead compound of this series **1** was transported into tumor cells by RFC and PCFT (FR uptake was not evaluated).^5^ Compound **1** accumulated in the cytosol and mitochondria whereupon it inhibited C1 metabolism at critical mitochondrial (SHMT2) and cytosolic (SHMT1, GARFTase, AICARFTase) targets.^5,36^ Analogue **1** showed *in vitro* antitumor efficacy toward pancreatic cancer, lung adenocarcinoma, and colon cancer cell lines, with promising *in vivo* efficacy against early and late-stage MIA PaCa 2 pancreatic cancer xenografts.^5,36^

Compound **1** is a first-in-class classical antifolate with a unique spectrum of multitargeted inhibitory activity including SHMT in mitochondria and *de novo purine* biosynthesis in the cytosol.^36^ SHMT2 is a bona fide tumor target with significant impacts on glycine and formate availability for cell proliferation and redox homeostasis.^21,34,70^ There are compelling arguments for targeting *de novo* purine biosynthesis for cancer, as antipurine drugs inhibit tumors independent of wild-type p53 status,^71^ show selectivity based on loss of purine salvage^72^ and suppress mTOR signaling.^73^

The major goal of this investigation was to systematically interrogate the structure–activity profiles for pyrrolo[3,2-*d*]pyrimidine compounds related to compound **1** and similar compounds **2**–**4** from our prior study.^36^ We explored the impact of modifications of the bridge (length, heteroatom substitutions) and the bridge aromatic ring (phenyl, thiophene) including the effects of fluorine substitutions. To facilitate our SAR analysis, we determined the X-ray crystal structures of human SHMT2 complexed with **1** and **14**, and the X-ray crystal structures of human GARFTase in complex with **1**, **2**, **3**, and **14**. We also used molecular modeling based on our SHMT2 and GARFTase structures, as well as published X-ray crystal structures of ATIC,^37^ SHMT1,^19^ FRα,^38^ and FRβ,^39^ to design our multitargeted C1 inhibitors. We tested 11 new analogues and compared results to those for **1**, **2**, **3**, and **4** using a range of biological assays to measure transport and target engagement, with antitumor validation by *in vitro* efficacy studies in KB nasopharengeal tumor cells and HPAC pancreatic cancer cells, with parallel assays with normal (HPNE) cells.

In general, *in vitro* inhibition of SHMT1 paralleled the inhibition of SHMT2, as expected given the close homology between these proteins (66% sequence identity). In terms of SHMT2 inhibition, compounds with a 5-carbon bridge were the most potent, with compound **11** showing a remarkable 28-fold increase in SHMT2 inhibitory activity over the previous lead analogue **1** (4-carbon bridge). Significant albeit progressively decreased inhibition of SHMT2/SHMT1 was seen with the 4- (**1**, **5**, **14**, **15**, and **16**) and 3-carbon bridge analogues (**2**, **13**, and **16**). Although the nature of the bridge aromatic ring (phenyl versus thiophene) had no consistent impact on enzyme target inhibition, heteroatom replacements in the bridge region were generally detrimental to inhibition of both SHMT2 and SHMT1 except for oxygen, which provided similar inhibition compared to the corresponding carbon bridge analogue. For GARFTase, both the 4- and 5-atom bridge appeared to enhance inhibition, with increased inhibition by **15** (11-fold), **3** (9.5-fold), and **9** (8.7-fold) over **1**. For ATIC (AICARFTase), structural impacts on enzyme inhibition were more subtle, although a bridge thiophene (**14**) increased the potency ∼4-fold over **1** with a phenyl ring.

We established a broad spectrum capacity for selective FR-targeting for this series with generally limited transport for RFC and PCFT. With the exception of **1**, **2**, **4**, **6**, and **13**, all compounds were inactive for RFC and/or PCFT. Interestingly, PCFT-targeting appeared to be enhanced by fluorination (compare **13** to **2** and **1** to **15**). By metabolite rescue studies in KB cells, we identified mitochondrial C1 metabolism and *de novo* purine nucleotide biosynthesis as the targeted pathways by the pyrrolo[3,2-*d*]pyrimidine antifolates. By *in vitro* enzyme assays, we confirmed inhibition of SHMT2 by our pyrrolo[3,2-*d*]pyrimidine inhibitors over a 1331-fold range, as well as direct inhibition of the purine biosynthetic enzymes at GARFTase and/or AICARFTase. Our discovery of novel compounds that target both mitochondrial and cytosolic C1 metabolism is particularly noteworthy in that by direct targeting mitochondrial C1 metabolism, this would exacerbate the impact of inhibiting cytosolic targets by limiting the source of C1 units for cytosolic anabolism.^21^ Further, inhibition of both SHMT1 as well as SHMT2 is essential as this prevents metabolic “compensation” and reversal of SHMT1 (serine → glycine) in response to loss of SHMT2.^35^

Of course, for all of these cellular targets, extending *in vitro* results with monoglutamyl antifolate compounds to cells must be done with caution, reflecting the potential impact of membrane transport and polyglutamate synthesis on enzyme inhibition by antifolates in cells.^16^ As previously reported,^5^**1** is metabolized to polyglutamate forms in both the cytosol and mitochondria of MIA PaCa2 pancreatic cancer cells. In the present study, toward KB tumor cells, **1** was the best inhibitor of cell proliferation, and **13** was somewhat more potent than **1** toward HPAC pancreatic cancer cells. These results may reflect cellular pharmacodynamic determinants of drug activity. This is reflected in the results with compound **9**, considered the most promising compound of the series from SAR and enzymology studies but moderately inhibitory toward KB and HPAC tumors. SAR studies to further optimize all four targets’ inhibitory potencies in a single molecule and improve KB and HPAC anti-tumor activity are currently underway.

In conclusion, our results document novel first-in-class conformationally flexible pyrrolo[3,2-*d*]pyrimidine antifolate inhibitors which provide tumor selectivity via FR selectivity and inhibition of multiple essential metabolic pathways for malignant cells including mitochondrial C1 metabolism at SHMT2 and *de novo* purine nucleotide biosynthesis at GARFTase and ATIC. Multitargeted inhibitors such as those described would offer substantial advantages in circumventing resistance to single-target drugs. The specificity of these agents for FRs over the ubiquitously expressed RFC, and inhibition of *de novo* purine nucleotide biosynthesis and mitochondrial C1 metabolism are especially notable given the association of these pathways with malignant cells.^11,13,18,19,21,74^ These novel multitargeted pyrrolo[3,2-*d*]pyrimidine agents represent an exciting new structural motif for targeted cancer therapy with substantial advantages of selectivity and potency over clinically used antifolates.^74^

## Experimental Section

### General Chemistry

All evaporations were carried out at reduced pressure with a rotary evaporator. Analytical samples were dried *in vacuo* in a CHEM-DRY drying apparatus over P_2_O_5_ at 50 °C. Melting points were determined either using a MEL-TEMP, II melting point apparatus with FLUKE 51 K/J electronic thermometer or using an MPA100 OptiMelt automated melting point system and are uncorrected. Nuclear magnetic resonance spectra for proton (^1^H NMR) were recorded on the Bruker Avance II 400 (400 MHz) or Bruker Avance II 500 (500 MHz) NMR systems with TopSpin processing software. The chemical shift values (δ:) are expressed in, (parts per million) relative to tetramethylsilane as an internal standard: s, singlet; d, doublet; dd, doublet of doublet; t, triplet; q, quartet; m, multiplet; br, broad singlet; td, triplet of doublet; dt, doublet of triplet; quin, quintet; exch., exchangeable using D_2_O. Thin-layer chromatography (TLC) was performed on Whatman PE SIL G/UV254 flexible silica gel plates and the spots were visualized under 254 and 365 nm ultraviolet illumination. Proportions of solvents used for TLC are by volume. All analytical samples were homogeneous on TLC in at least two different solvent systems. Column chromatography was performed on silica gel (70 to 230 mesh, Fisher Scientific) column. Flash chromatography was carried out on the CombiFlash *Rf* systems, model COMBIFLASH *RF*. Pre-packed RediSep *Rf* normal-phase flash columns (230 to 400 mesh) of diverse sizes were used. The amount (weight) of silica gel for column chromatography was in the range of 50–100 times the amount (weight) of the crude compounds being separated. Elemental analyses were performed by Atlantic Microlab, Inc., Norcross, GA. Element compositions are within ±0.4% of the calculated values. Fractional moles of water or organic solvents frequently found in some analytical samples could not be prevented despite 24 to 48 h of drying *in vacuo* and were confirmed where possible by their presence in the ^1^H NMR spectra. The ^13^C NMR data was collected using Bruker Avance II 400 (400 MHz) NMR system with TopSpin processing software or Bruker 600 MHz Avance Neo NMR with a TCI Cryo Probe. The high-performance liquid chromatography (HPLC) was performed using UltiMate 3000 UHPLC+ system. Reversed-phase HPLC was carried out XSelect CSH C18 XP, 130Å, 2.5 μm, 3 mm × 100 mm column. Solvent A: water with 0.1% trifluoroacetic acid (TFA); Solvent B: acetonitrile. Mass spectrometry *m*/*z* determinations were performed by an Advion Expression-S CMS (a single quadrupole compact MS) controlled by Advion Chems Express 4.0.13.8 software. High-resolution mass spectroscopy (HRMS) was performed on a Thermo Scientific Q Exactive high-resolution mass spectrometer or Thermo Scientific Finnegan LTQ Orbitrap XL mass spectrometer. Purification and separation of compounds are carried out using Buchi Pure C-850 FlashPrep and XBridge Prep C18 5μM OBD 19 mm × 150 mm column. The purities of the final compounds determined by HPLC analysis were >95%.

Synthesis of **2**, **3**, **4**, **9**, **15**, and **16** are already published.^36^

#### General Procedure for the Synthesis of **18a**–**b**

Ethyl 4-hydroxybenzoate (**17**) (1 equiv), potassium carbonate (1.5 equiv), and appropriate alcohol (1.5 equiv) were added to 200 mL of acetonitrile, and the suspension was refluxed at 95 °C for 16 h. The solvent was evaporated under reduced pressure and to the solids were added water (20 mL) and ethyl acetate (3 × 50 mL). To the ethyl acetate layer was added anhyd. sodium sulfate, and the solution was filtered. Silica gel was added to the solvent and a plug was prepared. Flash chromatography was carried out using ethyl acetate-hexane to afford **18a**–**b**.

##### Ethyl 4-(2-Hydroxyethoxy)benzoate (**18a**)

Compound **18a** was synthesized using the general method described for the preparation of **18a**–**b** using ethyl 4-hydroxybenzoate (**17**) (6.00 g, 35.75 mmol), potassium carbonate (7.41 g, 53.62 mmol), and 2-bromoethanol (3.8 mL, 53.62 mmol), to give **18a** as a colorless oil (3.3 g, 43.9%); TLC *R*_*f*_ = 0.22 (EtOAc/Hexane, 1:2); ^1^H NMR (500 MHz, DMSO-*d*_6_) δ: 7.91 (d, *J* = 8.9 Hz, 2H, Ar), 7.08–7.03 (d, 2H, Ar), 4.92 (t, *J* = 5.7 Hz, 1H, exch., −OH), 4.28 (q, *J* = 7.1 Hz, 2H, −CH_2_−), 4.09–4.05 (m, 2H, −CH_2_−), 3.74 (dd, *J* = 10 Hz, 2H, −CH_2_−), 1.31 (t, *J* = 7.1, 3H, −CH_3_). This compound was used for the next reaction without further characterization.

##### Ethyl 4-(3-Hydroxypropoxy)benzoate (**18b**)

Compound **18b** was synthesized using the general method described for the preparation of **18a**–**b** using ethyl 4-hydroxybenzoate (**17**) (6 g, 39.44 mmol), potassium carbonate (8.18 g, 59.15 mmol), and 3-bromopropanol (8.22 g, 59.15 mmol), to give **18b** as a colorless oil (6.2 g, 75%); TLC *R*_*f*_ = 0.13 (EtOAc/Hexane, 1:2); ^1^H NMR (400 MHz, DMSO-*d*_6_) δ: 7.90 (dd, *J* = 8.8, 2.5 Hz, 2H, Ar), 7.16–6.95 (m, 2H, Ar), 4.61 (t, 2.2 Hz, 1H, exch., −OH), 4.26 (qd, *J* = 7.2, 2.5 Hz, 2H, −CH_2_−), 4.11 (td, *J* = 6.5, 2.5 Hz, 2H, −CH_2_−), 3.58 (qd, *J* = 6.0, 2.1 Hz, 2H, −CH_2_−), 1.89 (pd, *J* = 6.3, 2.2 Hz, 2H, −CH_2_−), 1.30 (td, *J* = 7.1, 2.5 Hz, 3H, −CH_3_). This compound was used for the next reaction without further characterization.

#### General Procedure for the Synthesis of **19a**–**b, 23**, and **31e**

To the alcohols **18a**–**b, 22** and **30e** were dissolved in dichloromethane (25 mL) and triethylamine (1 equiv) was added to the mixture. The mixture was cooled to 0 °C and stirred for 10 min. An addition funnel was attached at the top of the RBF, created vacuum inside the system, and then nitrogen gas was purged to create inert atmosphere. With the use of the addition funnel, under anhyd. conditions, methanesulfonyl chloride (1.05 equiv) was added dropwise over 30 min. The reaction was stirred at rt for 1 h and then the reaction was directly added to sodium bicarbonate solution (50 mL) in a separatory funnel and the bottom dichloromethane layer was collected. The water layer was washed with dichloromethane (3 × 100 mL). The dichloromethane was evaporated to obtain a semisolid product. To the intermediate in acetone, sodium iodide (1 equiv) was added and refluxed for 8 h. The reaction mixture was filtered. The filtrate was evaporated to obtain **19a**–**b, 23**, and **31e**.

##### Ethyl 4-(2-Iodoethoxy)benzoate (**19a**)

Compound **19a** was prepared using the general method described for the preparation of **19a**–**b, 23**, and **31e**, from **18a** (2.5 g, 11.89 mmol), methanesulfonyl chloride (1.08 mL, 13.91 mmol), and triethylamine (1.82 mL, 13.08 mmol) to form the intermediate product. To this was added sodium iodide (1.98 g, 13.09 mmol), and the general method was followed to give 1.45 g (38%) of **19a** as a colorless oil; TLC *R*_*f*_ = 0.7 (EtOAc/Hexane, 1:2); ^1^H NMR (500 MHz, DMSO-*d*_6_) δ: 7.94–7.89 (m, 2H, Ar), 7.09–7.05 (m, 2H, Ar), 4.34 (t, *J* = 6.1 Hz, 2H, −CH_2_−), 4.28 (q, *J* = 7.1 Hz, 2H, −CH_2_−), 3.55 (t, *J* = 6.1 Hz, 2H, −CH_2_−), 1.31 (t, *J* = 7.1 Hz, 3H, −CH_3_). This compound was used for the next reaction without further characterization.

##### Ethyl 4-(3-Iodopropoxy)benzoate (**19b**)

Compound **19b** was prepared using the general method described for the preparation of **19a**–**b, 23**, and **31e**, from **18b** (2.5 g, 11.15 mmol), methanesulfonyl chloride (1 mL, 13.02 mmol), and triethylamine (1.71 mL, 12.26 mmol) to form the intermediate. To this sodium iodide (1.24 g, 12.26 mmol) was added, and the procedure was followed to give 1.8 g (48%) of **19b** as a colorless oil; TLC *R*_*f*_ = 0.55 (EtOAc/Hexane, 1:2); ^1^H NMR (500 MHz, DMSO-*d*_6_) δ: 7.90 (dd, *J* = 9.0, 2.5 Hz, 2H, Ar), 7.02 (dd, *J* = 9.2, 2.4 Hz, 2H, Ar), 4.26 (q, *J* = 7.0 Hz, 2H, −CH_2_−), 4.11 (t, *J* = 6.4 Hz, 2H, −CH_2_−), 3.56 (dt, *J* = 9.2, 5.4 Hz, 2H, −CH_2_−), 1.89 (p, *J* = 6.2 Hz, 2H, −CH_2_−), 1.30 (t, *J* = 7.1 Hz, 3H, CH_3_). This compound was used for the next reaction without further characterization.

##### Methyl 4-Mercaptobenzoate (**21**)

To a solution of 4-mercaptobenzoic acid (**20**) (12 g, 64.86 mmol) in 200 mL of methanol was added 4 mL of concentrated sulfuric acid. The reaction was refluxed at 65 °C for 6 h. Silica gel (400 mg) was then added, and the solvent was evaporated under reduced pressure. The resulting plug was loaded onto a silica gel column with ethyl acetate-hexane as eluent. Fractions that showed the desired spot (TLC) were pooled and the solvent evaporated to dryness to **21** (9.8 g, 70%) as a pale liquid.^61^ TLC *R*_*f*_ = 0.58 (EtOAc/Hexane, 1:2); ^1^H NMR (400 MHz, DMSO-*d*_6_) δ: 7.86 (dd, *J* = 8.5, 1.7 Hz, 2H, Ar), 7.39 (dd, *J* = 8.5, 1.7 Hz, 2H, Ar), 3.83 (s, 1H, −OCH_3_), 3.60 (s, 1H, exch., -SH). The ^1^H NMR matches the ^1^H NMR reported in the literature.^58^

##### Methyl 4-((3-Hydroxypropyl)thio)benzoate (**22**)

Methyl 4-mercaptobenzoate (**21**) (5 g, 27.44 mmol), cesium carbonate (13.41 g, 41.16 mmol), and 2-bromopropanol (5.72 g, 41.16 mmol) were added to 200 mL of acetonitrile, and the suspension was refluxed at 95 °C for 16 h. The solvent was evaporated under reduced pressure, and to the solids were added water (20 mL) and ethyl acetate (3 × 50 mL). To the ethyl acetate layer was added anhyd. sodium sulfate, and the solution was filtered. Silica gel was added to the solvent and a plug was prepared. A flash column chromatography was carried out using ethyl acetate-hexane to afford **22** (3.2 g, 55%) as a colorless liquid; TLC *R*_*f*_ = 0.30 (EtOAc/Hexane, 1:2); ^1^H NMR (400 MHz, DMSO-*d*_6_) δ: 7.86 (dd, *J* = 8.7, 2.1 Hz, 2H, Ar), 7.39 (dd, *J* = 8.5, 1.7 Hz, 2H, Ar), 4.61 (t, *J* = 5.2 Hz, 1H, exch., −OH), 3.83 (s, 3H, −OCH_3_), 3.52 (q, *J* = 6.0 Hz, 2H, −CH_2_−), 3.09 (t, *J* = 7.3 Hz, 2H, −CH_2_−), 1.83–1.70 (m, 2H, −CH_2_−). The ^1^H NMR matches the ^1^H NMR reported in the literature.^75^

##### Methyl 4-((3-Iodopropyl)thio)benzoate (**23**)

Compound **23** was prepared using the general method described for the preparation of **19a**–**b**, **23**, and **31e**, from compound **22** (2.5 g, 10.40 mmol), methanesulfonyl chloride (1 mL, 13.02 mmol), and triethylamine (1.60 mL, 11.44 mmol) to form the intermediate. To this, sodium iodide (1.72 g, 11.44 mmol) was added, and the procedure was followed to give 1.5 g (41%) of **23** as a colorless oil; TLC *R*_*f*_ = 0.80 (EtOAc/Hexane, 1:2); ^1^H NMR (400 MHz, DMSO-*d*_6_) δ: 7.87 (d, *J* = 8.4 Hz, 2H, Ar), 7.42 (d, *J* = 8.4 Hz, 2H, Ar), 3.83 (s, 3H, −OCH_3_), 3.35 (t, *J* = 6.7 Hz, 2H, −CH_2_−), 3.13 (q, *J* = 7.1, 6.7 Hz, 2H, −CH_2_−), 2.08 (p, *J* = 6.8 Hz, 2H, −CH_2_−). This compound was used for the next reaction without further characterization.

##### Methyl 4-((3-Hydroxypropyl)amino)benzoate (**25**)

Methyl 4-iodobenzoate (**24**) (7 g, 26.71mmol) and DMSO (5ml) were added to a mixture of copper iodide (1.02 g, 5.34 mmol) and l-proline (1.23 g, 10.69 mmol), and the resulting blue solution was stirred for 5 min, before adding 3-aminopropanol (10 g, 133.56 mmol). The resulting mixture was stirred under argon at rt for 16 h. Upon completion, the reaction mixture was diluted with 30 mL of water and extracted 2 × 150 mL of ethyl acetate. The combined organic layers were successively washed with brine. Silica gel was then added, and the solvent was evaporated under reduced pressure. The resulting plug was loaded onto a silica gel column with ethyl acetate-hexane as the eluent. Fractions that showed the desired spot (TLC) were pooled and the solvent evaporated to dryness to afford **25** as a colorless oil (4.2 g, 75%); TLC *R*_*f*_ = 0.38 (MeOH/CHCl_3_/NH_4_OH, 1:10:0.5); ^1^H NMR (400 MHz DMSO-*d*_6_) δ: 7.68 (d, *J* = 8.8 Hz, 2H, Ar), 6.58 (d, *J* = 8.8 Hz, 2H, Ar), 6.51 (t, *J* = 4.0 Hz, 1H, exch., −NH), 4.51 (s, 1H, exch., −OH), 3.74 (s, 3H, −OCH_3_), 3.50 (t, *J* = 6.2 Hz, 2H, −CH_2_−), 3.18–3.07 (m, 2H, −CH_2_−), 1.70 (p, *J* = 6.6 Hz, 2H, −CH_2_−). This compound was taken for the next reaction without further characterization.

##### Methyl 4-((3-Iodopropyl)amino)benzoate (**27**)

To a solution of triphenylphosphine (2.51 g, 9.56 mmol) in dry methylene chloride (100 mL) were added iodine (2.43 g, 9.56 mmol) and imidazole (0.65 g, 9.56 mmol) at 0 °C. The resulting solution was stirred for 10 min, before a solution of methyl 4-((3-hydroxypropyl)amino)benzoate (**25**) (2 g, 9.56 mmol) in dry methylene chloride (50 mL) was added. The reaction mixture was then quenched with sat. aqueous sodium thiosulfate solution after 30 min. The organic layer was separated, washed with brine, and dried over Na_2_SO_4_. Silica gel was then added, and the solvent was evaporated under reduced pressure. The resulting plug was loaded onto a silica gel column with ethyl acetate-hexane as the eluent. Fractions that showed the desired spot (TLC) were pooled and the solvent evaporated to dryness to **27** (2.5 g, 82%) as a colorless liquid; TLC *R*_*f*_ = 0.50 (MeOH/CHCl_3_/NH_4_OH, 1:10:0.5); ^1^H NMR (400 MHz, DMSO-*d*_6_) δ: 7.70 (d, *J* = 8.4 Hz, 2H, Ar), 6.74–6.55 (m, 3H, Ar and exch. NH), 3.74 (s, 3H, −OCH_3_), 3.56 (t, *J* = 6.0 Hz, 2H, −CH_2_−), 3.16 (q, *J* = 5.9 Hz, 2H, −CH_2_−), 2.03 (p, *J* = 7.6, 7.2 Hz, 2H, −CH_2_−). This compound was used for the next reaction without further characterization.

#### General Procedure for the Synthesis of **29a**–**e**

To a 20 mL vial for a microwave reaction was added a mixture of palladium chloride (71 mg, 0.40 mmol), triphenylphosphine (131 mg, 0.40 mmol), triethylamine (10.1 g, 100 mmol), methyl 4-bromo-2-fluorobenzoate **28a** (2.5 g, 10.73 mmol), or methyl 4-bromo-2,6-difluorobenzoate **28b** (2.5g, 9.96 mmol), or methyl 4-bromo-3-fluorothiophene-2-carboxylate **28c** (2.5 g, 10.46 mmol), and anhyd. acetonitrile (10 mL). To the stirred mixture were added copper(I) iodide (304 mg, 1.60 mmol) and the appropriate alkyne alcohol (1.05 equiv), and the vial was sealed and maintained in the microwave reactor at 100 °C for 1 h. Silica gel (5 g) was added, and the solvent was evaporated under reduced pressure. The resulting plug was loaded onto a silica gel column (3.5 × 12 cm) and eluted with hexane followed by 20–30% EtOAc in hexane. The desired fractions (TLC) were collected, and the solvent was evaporated under reduced pressure to afford **29a**–**e**.

##### Methyl 2-Fluoro-4-(3-hydroxyprop-1-yn-1-yl)benzoate (**29a**)

Compound **29a** was synthesized using the general method described for the preparation of **29a**–**e** using methyl 4-bromo-2-fluorobenzoate **28a** (2.5 g, 10.73 mmol), prop-2-yn-1-ol (1.2 mL, 16.09 mmol), to give 2.02 g of **29a** as a yellow semisolid (2.02 g, 90.6%); TLC *R*_*f*_ = 0.3 (EtOAc/Hexane, 1:1); ^1^H NMR (400 MHz, DMSO-*d*_6_) δ: 7.88 (t, *J* = 7.9 Hz, 1H, Ar), 7.47–7.35 (m, 2H, Ar), 5.46 (s, 1H, exch., −OH), 4.34 (s, 2H, −CH_2_−), 3.86 (s, 3H, −OCH_3_). This compound was used for the next reaction without further characterization.

##### Methyl 2-Fluoro-4-(4-hydroxybut-1-yn-1-yl)benzoate (**29b**)

Compound **29b** was synthesized using the general method described for the preparation of **29a**–**e** using methyl 4-bromo-2-fluorobenzoate **28a** (2.5 g, 10.73 mmol), but-3-yn-1-ol (0.6 mL, 8 mmol), to give 1.86 g of **29b** as a yellow semisolid (1.86 g, 78%); TLC *R*_*f*_ = 0.3 (EtOAc/Hexane, 1:1); ^1^H NMR (400 MHz, DMSO-*d*_6_) δ: 7.85 (t, *J* = 8.0 Hz, 1H, Ar), 7.42–7.30 (m, 2H, Ar), 4.98 (t, *J* = 5.6 Hz, 1H, exch., −OH), 3.85 (s, 3H, −OCH_3_), 3.60 (td, *J* = 6.7, 5.6 Hz, 2H, −CH_2_−), 2.60 (t, *J* = 6.7 Hz, 2H, −CH_2_−). This compound was used for the next reaction without further characterization.

##### Methyl 2-Fluoro-4-(5-hydroxypent-1-yn-1-yl)benzoate (**29c**)

Compound **29c** was synthesized using the general method described for the preparation of **29a**–**e** using methyl 4-bromo-2-fluorobenzoate **28a** (2.5 g, 10.73 mmol), pent-4-yn-1-ol (1.5 mL, 16.01 mmol), to give 2.23 g of **29c** as a yellow semisolid (2.01 g, 79%); TLC *R*_*f*_ = 0.3 (EtOAc/Hexane, 1:1); ^1^H NMR (400 MHz, DMSO-*d*_6_) δ: 7.85 (t, *J* = 7.9 Hz, 1H, Ar), 7.38 (d, *J* = 11.6 Hz, 1H, Ar), 7.33 (d, *J* = 8.1 Hz, 1H, Ar), 6.67 (t, *J* = 3.0 Hz, 0H), 4.57 (t, *J* = 10.4 Hz, 1H, exch., −OH), 3.85 (s, 3H, −OCH_3_), 3.52 (q, *J* = 5.8 Hz, 2H, −CH_2_−), 1.69 (q, *J* = 6.7 Hz, 2H, −CH_2_−). This compound was used for the next reaction without further characterization.

##### Methyl 2,6-Difluoro-4-(4-hydroxybut-1-yn-1-yl)benzoate (**29d**)

Compound **29d** was synthesized using the general method described for the preparation of **29a**–**e** using methyl 4-bromo-2,6-difluorobenzoate **28b** (2.5g, 9.96 mmol), but-3-yn-1-ol (0.5 mL, 8 mmol), to give 1.3 g of **29d** as a brown solid (1.36 g, 85%); TLC *R*_*f*_ = 0.3 (EtOAc/Hexane, 1:1); mp, 123–131 °C; ^1^H NMR (500 MHz, DMSO-*d*_6_) δ: 7.34–7.30 (m, 2H), 4.96 (t, *J* = 5.6 Hz, 1H), 3.95–3.88 (m, 1H), 3.89 (s, 3H), 3.60 (td, *J* = 6.6, 5.6 Hz, 2H), 2.60 (t, *J* = 6.7 Hz, 2H). This compound was used for the next reaction without further characterization.

##### Methyl 3-Fluoro-4-(4-hydroxybut-1-yn-1-yl)thiophene-2-carboxylate (**29e**)

Compound **29e** was synthesized using the general method described for the preparation of **29a**–**e**, using methyl 4-bromo-3-fluorothiophene-2-carboxylate **28c** (2.5 g, 10.46 mmol), but-3-yn-1-ol (1.5 mL, 16.01 mmol), to give 1.63 g of **29e** as a yellow oil (79%); TLC *R*_*f*_ = 0.3 (EtOAc/Hexane, 1:1); ^1^H NMR (500 MHz, DMSO-*d*_6_) δ: 8.08 (d, *J* = 4.2 Hz, 1H, Ar), 4.84 (s, 1H, −OH, exch), 3.84 (s, 3H, −OCH_3_), 3.58 (t, *J* = 6.7 Hz, 2H, −CH_2_−), 2.58 (t, *J* = 6.6 Hz, 2H, −CH_2_−). This compound was used for the next reaction without further characterization.

#### General Procedure for the Synthesis of **30a**–**e**

To a Parr flask were added **29a**–**e**, 10% palladium on activated carbon (50% w/w), and MeOH (100 mL). Hydrogenation was carried out at 55 psi of H_2_ for 14 h. The reaction mixture was filtered through Celite, washed with MeOH (100 mL), and concentrated under reduced pressure to give a crude mixture containing **30a–e**. Without chromatographic separation, these compounds were used for the next reaction.

##### Methyl 2-Fluoro-4-(3-hydroxypropyl)benzoate (**30a**)

Compound **30a** was prepared using the general method described for the preparation of **30a**–**e**, from **29a** (2.02 g, 9.7 mmol) to give 1.98 g (96%) of **30a** as a colorless oil; TLC *R*_*f*_ = 0.3 (EtOAc/Hexane, 1:1); ^1^H NMR (400 MHz, DMSO-*d*_6_) δ: 7.81 (t, *J* = 7.8 Hz, 1H, Ar), 7.24–7.14 (m, 2H, Ar), 4.55 (s, 1H, exch., −OH), 3.84 (s, 3H, −OCH_3_), 3.41 (d, *J* = 6.3 Hz, 2H, −CH_2_−), 2.73–2.64 (m, 2H, −CH_2_−), 1.78–1.68 (m, 2H, −CH_2_−). This compound was used for the next reaction without further characterization.

##### Methyl 2-Fluoro-4-(4-hydroxybutyl)benzoate (**30b**)

Compound **30b** was prepared using the general method described for the preparation of **30a**–**e**, from **29b** (1.86 g, 8.4 mmol) to give 1.68 g (89%) of **30b** as a colorless oil; TLC *R*_*f*_ = 0.3 (EtOAc/Hexane, 1:1); ^1^H NMR (500 MHz, DMSO-*d*_6_) δ: 7.80 (t, *J* = 7.9 Hz, 1H, Ar), 7.22–7.14 (m, 2H, Ar), 4.39 (s, 1H, exch., −OH), 3.84 (s, 3H, −OCH_3_), 3.40 (d, *J* = 11.7 Hz, 2H, −CH_2_−), 2.65 (t, *J* = 7.7 Hz, 2H, −CH_2_−), 1.66–1.55 (m, 2H, −CH_2_−), 1.42 (dt, *J* = 13.4, 6.5 Hz, 2H, −CH_2_−). This compound was used for the next reaction without further characterization.

##### Methyl 2-Fluoro-4-(5-hydroxypentyl)benzoate (**30c**)

Compound **30c** was prepared using the general method described for the preparation of **30a**–**e**, from **29c** (2.23 g, 9.44 mmol) to give 2.07 g (91%) of **30c** as a colorless oil; TLC *R*_*f*_ = 0.3 (EtOAc/Hexane, 1:1); ^1^H NMR (400 MHz, DMSO-*d*_6_) δ: 7.80 (t, *J* = 7.9 Hz, 1H, Ar), 7.24–7.13 (m, 2H, Ar), 4.37 (s, 1H, exch., −OH), 3.84 (s, 3H, −OCH_3_), 3.37 (t, *J* = 6.4 Hz, 2H, −CH_2_−), 2.69–2.60 (m, 2H, −CH_2_−), 1.58 (p, *J* = 7.6 Hz, 2H, −CH_2_−), 1.44 (dd, *J* = 14.2, 7.4 Hz, 2H, −CH_2_−), 1.35–1.23 (m, 2H, −CH_2_−). This compound was used for the next reaction without further characterization.

##### Methyl 2,6-Difluoro-4-(4-hydroxybutyl)benzoate (**30d**)

Compound **30d** was prepared using the general method described for the preparation of **30a**–**e**, from **29d** (1.86 g, 8.4 mmol) to give 1.73 g (85%) of **30d** as a colorless oil; TLC *R*_*f*_ = 0.3 (EtOAc/Hexane, 1:1); ^1^H NMR (500 MHz, DMSO-*d*_6_) δ: 7.12 (d, *J* = 10.2 Hz, 2H, Ar), 4.40 (t, *J* = 5.2 Hz, 1H, exch., −OH), 3.87 (s, 3H, −CH_3_), 3.40 (q, *J* = 6.1 Hz, 2H, −CH_2_−), 2.65 (t, *J* = 7.7 Hz, 2H, −CH_2_−), 1.61 (p, *J* = 7.6 Hz, 2H, −CH_2_−), 1.41 (p, *J* = 6.8 Hz, 2H, −CH_2_−). This compound was used for the next reaction without further characterization.

##### Methyl 3-Fluoro-4-(4-hydroxybutyl)thiophene-2-carboxylate (**30e**)

Compound **30e** was synthesized using the general method described for the preparation of **30a**–**e**, from **29e** (1.86 g, 8.4 mmol) to give 1.87 g (83%) of **30e** as a pale yellow oil; TLC *R*_*f*_*=* 0.3 (EtOAc/Hexane, 1:1); ^1^H NMR (500 MHz, DMSO-*d*_6_) δ: 7.71–7.63 (m, 1H, Ar), 4.40 (t, *J* = 5.0 Hz, 1H, exch., −OH), 3.80 (s, 3H, −OCH_3_), 3.41 (q, *J* = 6.0 Hz, 2H, −CH_2_−), 1.58 (pent, *J* = 7.6 Hz, 2H, −CH_2_−), 1.48–1.38 (m, 2H, −CH_2_−). This compound was used for the next reaction without further characterization.

#### General Procedure for the Synthesis of **31a**–**d**

To a solution of triphenylphosphine (1.5 equiv) in dry methylene chloride (100 mL) were added iodine (1.5 equiv) and imidazole (1.5 equiv) at 0 °C. The resulting solution was stirred for 10 min, before a solution of **30a**–**d** (1 equiv) in dry methylene chloride (50 mL) was added. The reaction mixture was then quenched with sat. aqueous sodium thiosulfate solution after 30 min. The organic layer was separated, washed with brine, and dried over Na_2_SO_4_. Silica gel was then added, and the solvent was evaporated under reduced pressure. The resulting plug was loaded onto a silica gel column with ethyl acetate-hexane as the eluent. Fractions with the desired peak were collected to obtain **31a**–**d**.

##### Methyl 2-Fluoro-4-(3-iodopropyl)benzoate (**31a**)

Compound **31a** was prepared using the general method described for the preparation of **31a**–**d**, from **30a** (2.2 g, 10.37 mmol) and triphenylphosphine (4.08 g, 15.55 mmol) in dry methylene chloride (120 mL) were added iodine (3.95 g, 15.55 mmol) and imidazole (1.06 g, 15.55 mmol), and the procedure was followed to give 2.33 g (70%) of **31a** as a colorless oil; TLC *R*_*f*_ = 0.8 (EtOAc/Hexane, 1:1); ^1^H NMR (400 MHz, DMSO-*d*_6_) δ: 7.82 (t, *J* = 7.9 Hz, 1H, Ar), 7.28–7.16 (m, 2H, Ar), 3.84 (s, 3H, −OCH_3_), 3.24 (t, *J* = 6.9 Hz, 2H, −CH_2_−), 2.79–2.70 (m, 2H, −CH_2_−), 2.13–2.04 (m, 2H, −CH_2_−). This compound was used for the next reaction without further characterization.

##### Methyl 2-Fluoro-4-(4-iodobutyl)benzoate (**31b**)

Compound **31b** was prepared using the general method described for the preparation of **31a**–**d**, from **30b** (1.98 g, 8.75 mmol) and triphenylphosphine (3.44 g, 13.13 mmol) in dry methylene chloride (100 mL) were added iodine (3.33 g, 13.13 mmol) and imidazole (0.89 g, 13.13 mmol), and the procedure was followed to give 2.07 g (81%) of **31b** as a colorless oil; TLC *R*_*f*_ = 0.8 (EtOAc/Hexane, 1:1); ^1^H NMR (500 MHz, DMSO-*d*_6_) δ: 7.86–7.77 (m, 1H, Ar), 7.25–7.13 (m, 2H, Ar), 3.84 (s, 3H, Ar), 3.32–3.25 (m, 2H, −CH_2_−), 2.67 (t, *J* = 7.4 Hz, 2H, −CH_2_−), 1.82–1.72 (m, 2H, −CH_2_−), 1.70–1.60 (m, 2H, −CH_2_−). This compound was used for the next reaction without further characterization.

##### Methyl 2-Fluoro-4-(5-iodopentyl)benzoate (**31c**)

Compound **31c** was prepared using the general method described for the preparation of **31a**–**d**, from **30c** (1.82 g, 7.57 mmol) and triphenylphosphine (2.98 g, 11.36 mmol) in dry methylene chloride (90 mL) were added iodine (2.88 g, 11.36 mmol) and imidazole (0.77 g, 11.36 mmol), and the procedure was followed to give 1.97 g (84%) of **31c** as a colorless oil; TLC *R*_*f*_*=* 0.8 (EtOAc/Hexane, 1:1); ^1^H NMR (400 MHz, DMSO-*d*_6_) δ: 7.81 (t, *J* = 7.9 Hz, 1H, Ar), 7.30–7.18 (m, 2H, Ar), 3.84 (s, 3H, −OCH_3_), 3.28 (t, *J* = 6.9 Hz, 2H, −CH_2_−), 2.66 (t, *J* = 7.6 Hz, 2H, −CH_2_−), 1.83–1.73 (m, 2H, −CH_2_−), 1.67–1.58 (m, 2H, −CH_2_−), 1.37 (q, *J* = 7.8 Hz, 2H, −CH_2_−). This compound was used for the next reaction without further characterization.

##### Methyl 2,6-Difluoro-4-(4-iodobutyl)benzoate (**31d**)

Compound **31d** was prepared using the general method described for the preparation of **31a**–**d**, from **30d** (3.0 g, 12.28 mmol) and triphenylphosphine (4.88 g, 18.42 mmol) in dry methylene chloride (150 mL) were added iodine (4.69 g, 18.42 mmol) and imidazole (1.27 g, 18.42 mmol), and the procedure was followed to give 2.81 g (69%) of **31d** as a colorless oil; TLC *R*_*f*_ = 0.8 (EtOAc/Hexane, 1:1); ^1^H NMR (500 MHz, DMSO-*d*_6_) δ: 7.14 (d, *J* = 10.1 Hz, 2H), 3.87 (s, 3H), 3.30 (t, *J* = 6.8 Hz, 2H), 2.67 (t, *J* = 7.4 Hz, 2H), 1.75 (p, *J* = 7.0 Hz, 2H), 1.67 (p, *J* = 7.4 Hz, 2H). This compound was used for the next reaction without further characterization.

##### Methyl 3-Fluoro-4-(4-iodobutyl)thiophene-2-carboxylate (**31e**)

Compound **31e** was prepared using the general method described for the preparation of **19a**–**b**, **23**, and **31e**, from compound **30e** (2.5 g, 10.40 mmol), methanesulfonyl chloride (0.96 mL, 12.34 mmol), and triethylamine (1.9 mL, 13.92 mmol) to form the intermediate. To this, sodium iodide was added and the procedure was followed to give 2.47 g (82%) of **31e** as a colorless oil; TLC *R*_*f*_ = 0.9 (EtOAc/Hexane, 1:1); ^1^H NMR (500 MHz, DMSO-*d*_6_) δ: 7.69 (d, *J* = 4.6 Hz, 1H, Ar), 3.81 (s, 3H, −CH_3_), 3.31 (t, *J* = 6.8 Hz, 2H, −CH_2_−), 2.55 (d, *J* = 7.5 Hz, 2H, −CH_2_−), 1.78 (p, *J* = 7.0 Hz, 2H, −CH_2_−), 1.64 (p, *J* = 7.5 Hz, 2H, −CH_2_−). This compound was used for the next reaction without further characterization.

#### General Procedure for the Synthesis of **33a**–**e**

To a solution of ethyl 3-amino-1*H-*pyrrole-2-carboxylate hydrochloride (0.5 g, 3.24 mmol) in dry DMF (10 mL) was slowly added NaH (0.17 g, 7.1 mmol) under nitrogen at rt. The resulting mixture was stirred for about 15 min when there was no more gas evolved, and then the appropriate iodide **31a**–**e** (1 equiv) was added. The reaction mixture was stirred at rt for 4 h, and DMF was evaporated at elevated temp. to offer a gummy residue **32a**–**e**, which was used for the next step without purification. The gummy residue was dissolved in MeOH (10 mL), and 1,3-bis(methoxycarbonyl)-2-methyl-2-thiopseudourea (0.7 g, 3.3 mmol) was added followed by AcOH (1.0 g, 15 mmol). The mixture was stirred at rt overnight and became a thick paste. NaOMe in MeOH (25%) (7 mL, 22 mmol) was added, and stirring was continued at rt overnight. The mixture was neutralized with AcOH, and the methanol was removed under reduced pressure. To the residue was added water (20 mL), and the pH was adjusted to 10–11 by adding NH_3_·H_2_O. The solid was collected by filtration and washed well with water. The resulting solid was added to 1 N NaOH (2 mL), and the mixture was heated at 55–65 °C for 3 h. The mixture was cooled and acidified using 1 N HCl. The precipitate was collected and dried overnight under reduced pressure to obtain **33a**–**e**.

#### General Procedure for the Synthesis of **34a**–**d**

Diethyl l-glutamate HCl (1 equiv) was dissolved in DMF (20 mL). In a separate flask, pteroic acid **33a**–**d** (1 equiv) was dissolved in DMF (20 mL). The solution of diethyl l-glutamate HCl (1 equiv) in DMF was then added, followed by DIPEA (3 equiv) and HATU (1 equiv). The mixture was stirred overnight at rt and then diluted with EtOAc (200 mL). The organic layer was washed sequentially with 50 mL of half-saturated sodium chloride, 50 mL of 10% citric acid (aq.), 50 mL of half-saturated sodium chloride, 50 mL of saturated sodium bicarbonate (aq.), 50 mL water, and then with 2 × 50 mL brine. The organic layer was dried over sodium sulfate, filtered, and concentrated under reduced pressure. Purification by column chromatography eluting 0–5% MeOH/DCM gave the desired product **34a**–**d**.

##### Diethyl (4-(3-(2-Amino-4-oxo-3,4-dihydro-5*H*-pyrrolo[3,2-*d*]pyrimidin-5-yl)propyl)-2-fluorobenzoyl)-l-glutamate (**34a**)

Using the general method for the synthesis of compounds **33a**–**e**, crude **32a** (1.1 g) was used to obtain **33a** (0.3 g, 0.9 mmol) as a white solid; TLC *R*_*f*_ = 0.0 (MeOH/CHCl_3_/HCl, 1:5:0.5); mp, 121.8–156.3 °C; ^1^H NMR (500 MHz, DMSO-*d*_6_) δ: 7.77 (t, *J* = 7.9 Hz, 1H, Ar), 7.24 (d, *J* = 2.9 Hz, 1H, Ar), 7.16–7.10 (m, 2H, Ar), 6.09 (s, 2H, exch., 2-NH_2_), 5.94 (d, *J* = 2.8 Hz, 1H, Ar), 4.26 (t, *J* = 7.0 Hz, 2H, −CH_2_−), 2.59 (dd, *J* = 8.9, 6.8 Hz, 2H, −CH_2_−), 2.09–2.02 (m, 2H, −CH_2_−). Using the general method for the synthesis of compounds **34a**–**d**, **33a** (0.15 g, 0.45 mmol) was used to obtain **34a** (0.18 g, 75%) as a light brown semisolid; TLC *R*_*f*_ = 0.3 (MeOH/CHCl_3_/NH_4_OH, 1:10:0.5); ^1^H NMR (500 MHz, DMSO-*d*_6_) δ: 11.06 (s, 1H, exch., −NH), 8.58 (dd, *J* = 7.6, 2.0 Hz, 1H, Ar), 7.51 (t, *J* = 7.8 Hz, 1H, Ar), 7.21 (d, *J* = 2.9 Hz, 1H, Ar), 7.18–7.08 (m, 2H, Ar), 5.91 (d, *J* = 2.8 Hz, 1H, Ar), 5.80 (s, 2H, exch., 2-NH_2_), 4.45–4.37 (m, 1H, −CH), 4.25 (t, *J* = 7.0 Hz, 2H, −CH_2_−), 4.12 (qq, *J* = 7.0, 3.7 Hz, 2H, −CH_2_−), 4.05 (q, *J* = 7.1 Hz, 2H, −CH_2_−), 3.86 (s, 2H, −CH_2_−), 2.58 (dt, *J* = 15.2, 8.0 Hz, 2H, −CH_2_−), 2.46–2.40 (m, 2H, −CH_2_−), 2.07–1.95 (m, 4 H, −CH_2_−), 1.19 (dt, *J* = 14.0, 7.1 Hz, 6 H, −CH_3_). This compound was used for the next reaction without further characterization.

##### Diethyl (4-(4-(2-Amino-4-oxo-3,4-dihydro-5*H-*pyrrolo[3,2-*d*]pyrimidin-5-yl)butyl)-2-fluorobenzoyl)-l-glutamate (**34b**)

Using the general method for the synthesis of compounds **33a**–**e**, crude **32b** (1.1 g) was used to obtain **33b** (0.25 g, 0.73 mmol) as a gray solid; TLC *R*_*f*_ = 0.0 (MeOH/CHCl_3_/HCl, 1:5:0.5); ^1^H NMR (400 MHz, DMSO-*d*_6_) δ: 7.76 (t, *J* = 8.1 Hz, 1H, Ar), 7.23 (d, *J* = 2.9 Hz, 1H, Ar), 7.16–7.05 (m, 2H, Ar), 6.03 (s, 2H, exch., 2-NH_2_), 5.91 (d, *J* = 2.8 Hz, 1H, Ar), 4.25 (t, *J* = 6.8 Hz, 2H, −CH_2_−), 2.62 (t, *J* = 7.7 Hz, 2H, −CH_2_−), 1.72 (p, *J* = 6.9 Hz, 2H, −CH_2_−), 1.48 (qd, *J* = 9.3, 8.8, 6.3 Hz, 2H, −CH_2_−). Using the general method for the synthesis of compounds **34a**–**d**, **33b** (0.15 g, 0.44 mmol) was used to obtain **34b** (0.1 g, 65%) as a brown semisolid; TLC *R*_*f*_ = 0.3 (MeOH/CHCl_3_/NH_4_OH, 1:10:0.5); ^1^H NMR (400 MHz, DMSO-*d*_6_) δ: 7.88 (s, 1H, exch., −NH), 7.55–7.45 (m, 2H, Ar), 7.17–7.06 (m, 2H, Ar), 6.15 (d, *J* = 2.8 Hz, 1H, Ar), 4.42 (s, 1H, −CH), 4.29 (t, *J* = 6.8 Hz, 2H, −CH_2_−), 4.17–4.01 (m, 4 H, −CH_2_−), 4.05–3.93 (m, 2H, −CH_2_−), 3.66 (t, *J* = 12.4 Hz, 2H, −CH_2_−), 2.64 (t, *J* = 7.6 Hz, 2H, −CH_2_−), 1.74 (m, 2H, −CH_2_−), 1.51 (d, *J* = 6.8 Hz, 2H, −CH_2_−), 1.26–1.13 (m, 6 H, −CH_3_). This compound was used for the next reaction without further characterization.

##### Diethyl (4-(5-(2-Amino-4-oxo-3,4-dihydro-5*H-*pyrrolo[3,2-*d*]pyrimidin-5-yl)pentyl)-2-fluorobenzoyl)-l-glutamate (**34c**)

Using the general method for the synthesis of compounds **33a**–**e**, crude **32c** (0.93 g) was used to obtain **33c** (0.15 g, 0.42 mmol) as a white solid; TLC *R*_*f*_ = 0.0 (MeOH/CHCl_3_/HCl, 1:5:0.5); mp, 135.3–148.1 °C; ^1^H NMR (500 MHz, DMSO-*d*_6_) δ: 10.45 (s, 1H, exch., −COOH), 7.76 (t, *J* = 7.9 Hz, 1H), 7.19–7.08 (m, 3H, Ar), 5.87 (d, *J* = 2.9 Hz, 1H, Ar), 5.76 (s, 2H, exch., −NH_2_), 4.20 (t, *J* = 7.1 Hz, 2H, −CH_2_−), 2.60 (t, *J* = 7.9 Hz, 2H, −CH_2_−), 1.73 (p, *J* = 7.3 Hz, 2H, −CH_2_−), 1.57 (p, *J* = 8.0 Hz, 2H, −CH_2_−), 1.20 (p, *J* = 7.9 Hz, 2H, −CH_2_−). This compound was used for the next reaction without further characterization. Using the general method for the synthesis of compounds **34a**–**d**, **33c** (0.15 g, 0.4 mmol) was used to obtain **34c** (0.07 g, 69%) as a gray solid TLC *R*_*f*_ = 0.3 (MeOH/CHCl_3_/NH_4_OH, 1:10:0.5); ^1^H NMR (400 MHz, DMSO-*d*_6_) δ: 11.17 (s, 1H, exch., −NH), 8.57 (dd, *J* = 7.5, 2.0 Hz, 1H, exch., −NH), 7.50 (t, *J* = 7.8 Hz, 1H, Ar), 7.38 (d, *J* = 2.9 Hz, 1H, Ar), 7.16–7.07 (m, 2H, Ar), 6.07 (d, *J* = 2.8 Hz, 1H, Ar), 4.43 (ddd, *J* = 9.5, 7.4, 5.1 Hz, 1H, −CH), 4.24 (t, *J* = 7.1 Hz, 2H, −CH_2_−), 4.12 (qq, *J* = 7.0, 3.7 Hz, 2H, −CH_2_−), 4.08–4.03 (m, 2H, −CH_2_−), 2.61 (t, *J* = 7.7 Hz, 2H, −CH_2_−), 2.46–2.40 (m, 2H, −CH_2_−), 2.09 (m, 2H, −CH_2_−), 1.75 (p, *J* = 7.3 Hz, 2H), 1.57 (q, *J* = 7.6 Hz, 2H), 1.22–1.17 (m, 8 H, −CH_2_– and −CH_3_). This compound was used for the next reaction without further characterization.

##### Diethyl (4-(4-(2-Amino-4-oxo-3,4-dihydro-5*H*-pyrrolo[3,2-*d*]pyrimidin-5-yl)butyl)-2,6-difluorobenzoyl)-l-glutamate (**34d**)

Using the general method for the synthesis of compounds **33a**–**e**, crude **32d** (0.93 g) was used to obtain **33d** (0.15 g, 0.42 mmol) as a white solid; TLC *R*_*f*_ = 0.0 (MeOH/CHCl_3_/HCl, 1:5:0.5); mp, 128–138.1 °C; ^1^H NMR (500 MHz, DMSO-*d*_6_) δ: 7.27–7.17 (m, 1H, Ar), 7.08–6.96 (m, 2H, Ar), 6.00–5.85 (m, 3H, Ar and exch. −NH_2_), 4.24 (dq, *J* = 13.2, 6.9 Hz, 2H, −CH_2_−), 2.61 (s, 2H, −CH_2_−), 1.71 (s, 2H, −CH_2_−), 1.48 (s, 2H, −CH_2_−). This compound was used for the next reaction without further characterization. Using the general method for the synthesis of compounds **34a**–**d**, **33d** (0.15 g, 0.4 mmol) was used to obtain **34d** (0.07 g, 62%) as a gray solid TLC *R*_*f*_ = 0.3 (MeOH/CHCl_3_/NH_4_OH, 1:10:0.5); ^1^H NMR (400 MHz, DMSO-*d*_6_) δ: 10.49 (s, 1H), 9.07 (d, *J* = 7.6 Hz, 1H), 7.21 (d, *J* = 2.8 Hz, 1H), 7.05–6.94 (m, 2H), 5.89 (d, *J* = 2.8 Hz, 1H), 5.80 (s, 2H), 4.43 (ddd, *J* = 9.8, 7.6, 5.1 Hz, 1H), 4.26 (t, *J* = 6.8 Hz, 2H), 4.20–4.01 (m, 4H), 2.61 (t, *J* = 7.7 Hz, 2H), 2.51–2.34 (m, 2H), 2.13–2.00 (m, 1H), 1.86 (dddd, *J* = 14.0, 9.7, 8.3, 6.0 Hz, 1H), 1.72 (p, *J* = 6.9 Hz, 2H), 1.53–1.41 (m, 2H), 1.19 (dt, *J* = 9.0, 7.1 Hz, 6H). This compound was used for the next reaction without further characterization.

##### Diethyl (4-(4-(2-Amino-4-oxo-3,4-dihydro-5*H*-pyrrolo[3,2-*d*]pyrimidin-5-yl)butyl)-3-fluorothiophene-2-carbonyl)-l-glutamate (**34e**)

Using the general method for the synthesis of compounds **33a**–**e**, crude **32e** (2.0 g) was used to obtain **33e** (0.11 g, 23%) as a white solid; TLC *R*_*f*_ = 0.0 (MeOH/CHCl_3_/HCl, 1:5:0.5); mp, 132.8–151.7 °C; ^1^H NMR (500 MHz, DMSO-*d*_6_) δ: 10.62 (s, 1H, exch., −COOH), 7.38 (s, 1H, Ar), 7.19 (d, *J* = 2.9 Hz, 1H, Ar), 5.88 (d, *J* = 2.9 Hz, 1H, Ar), 5.84 (s, 2H, exch., −NH_2_), 4.24 (t, *J* = 6.9 Hz, 2H, −CH_2_−), 2.50–2.39 (m, 2H, −CH_2_−), 1.73 (p, *J* = 7.1 Hz, 2H, −CH_2_−), 1.44 (h, *J* = 8.2 Hz, 2H, −CH_2_−). The compound was used for the next reaction without further characterization. To a solution of **33e** in anhyd. DMF (10 mL) was added to *N*-methylmorpholine (73 mg, 0.72 mmol) and 2-chloro-4,6*-*dimethoxy-1,3,5-triazine (127 mg, 0.72 mmol). The resulting mixture was stirred at rt for 2 h. To this mixture were added *N*-methylmorpholine (73 mg, 0.72 mmol) and l-glutamate diethyl ester hydrochloride (144 mg, 0.6 mmol). The reaction mixture was stirred for an additional 4 h at rt. Silica gel (400 mg) was then added, and the solvent was evaporated under reduced pressure. The resulting plug was loaded onto a silica gel column with 5% MeOH in CHCl_3_ as the eluent. Fractions that showed the desired spot (TLC) were pooled, and the solvent was evaporated to dryness to afford compound **34e**.

##### General Method for the Synthesis of Target Compounds (**1, 11**–**14**)

To a solution of **34a**–**e** were added 4 mL of methanol and 2 mL of 1 N NaOH. The reaction mixture was stirred for 1 h at rt, and the disappearance of the starting material was followed with TLC. The mixture was acidified to pH 2–3 using 1 N HCl to obtain target compounds (**1**, **11**–**14**) as precipitated residues on filtration.

##### (4-(3-(2-Amino-4-oxo-3,4*-*dihydro-5*H-*pyrrolo[3,2-*d*]pyrimidin-5-yl)propyl)-2-fluorobenzoyl)-l-glutamic Acid (**13**)

Using the general method for the synthesis of target compounds (**1**, **11**–**14**), **34a** (0.075 g, 0.15 mmol) was used to obtain **13** (0.04 g, 60%) as a white solid; TLC *R*_*f*_ = 0.0 (MeOH/CHCl_3_/HCl, 1:5:0.5); mp, 175.8–183.1 °C; ^1^H NMR (400 MHz, DMSO-*d*_6_) δ: 12.10–10.50 (s, br, 3H, exch., −COOH and −NH), 8.41 (dd, *J* = 7.6, 2.7 Hz, 1H, Ar), 7.53 (t, *J* = 7.8 Hz, 1H, Ar), 7.21 (d, *J* = 2.8 Hz, 1H, Ar), 7.21–7.09 (m, 2H), 5.91 (d, *J* = 2.8 Hz, 1H), 5.85 (s, 2H), 4.39 (ddd, *J* = 9.6, 7.6, 4.8 Hz, 1H), 4.25 (t, *J* = 7.0 Hz, 2H), 2.61–2.54 (m, 2H), 2.40–2.31 (m, 2H), 2.11–2.03 (m, 3H), 1.90 (dddd, *J* = 14.0, 9.5, 7.9, 6.3 Hz, 1H). ^13^C NMR (400 MHz, DMSO-*d*_6_) δ: 174.34, 173.56, 164.25, 161.04, 158.56, 154.95, 151.20, 147.50, 130.61, 128.21, 121.19, 116.28, 116.06, 112.12, 99.91, 52.32, 47.83, 32.66, 32.03, 30.64, 26.42. HPLC analysis: *t*_R_, 13.000 min; purity, 97.644% at 290 nm. HRMS calculated for C_21_H_22_FN_5_O_6_ [M + H]^+^, 460.1627. Found: 460.1627.

##### (4-(4-(2-Amino-4-oxo-3,4-dihydro-5*H*-pyrrolo[3,2-*d*]pyrimidin-5-yl)butyl)-2-fluorobenzoyl)-l-glutamic Acid (**1**)

Using the general method for the synthesis of target compounds (**1**, **11**–**14**), **34b** (0.40 g, 0.845 mmol) was used to obtain **1** (0.2 g, 56%) as a white solid; TLC *R*_*f*_ = 0.0 (MeOH/CHCl_3_/HCl, 1:5:0.5); mp, 161.6–164.4 °C; ^1^H NMR (500 MHz, DMSO-*d*_6_) δ: 8.43 (dd, *J* = 7.6, 2.6 Hz, 1H, exch., −NH), 7.51 (t, *J* = 7.7 Hz, 1H, Ar), 7.23 (d, *J* = 2.9 Hz, 1H, Ar), 7.13–7.05 (m, 2H, Ar), 6.2 (s, 2H, exch., −NH_2_), 5.92 (d, *J* = 2.8 Hz, 1H, Ar), 4.38 (ddd, *J* = 9.5, 7.5, 4.8 Hz, 1H, −CH), 4.25 (t, *J* = 6.8 Hz, 2H, −CH_2_−), 2.61 (t, *J* = 7.7 Hz, 2H, −CH_2_−), 2.40–2.28 (m, 2H, −CH_2_−), 2.08–1.84 (m, 2H, −CH_2_−), 1.72 (p, *J* = 7.1 Hz, 2H, −CH_2_−), 1.48 (td, *J* = 8.5, 4.1 Hz, 2H, −CH_2_−). Anal. Calcd. for C_22_H_24_FN_5_O_6_ 0.58 HCl: C, 55.81; H, 5.11; N, 14.79; F, 4.01. Found: C, 53.50; H, 5.15; N, 14.18; S, 3.81; Cl, 1.47. HPLC analysis: *t*_R_, 12.773 min; purity, 95.963% at 254 nm. MS calculated for C_22_H_24_FN_5_O_6_ [M + H]^+^, 474.46. Found: 474.6.

##### (4-(5-(2-Amino-4-oxo-3,4-dihydro-5*H-*pyrrolo[3,2-*d*]pyrimidin-5-yl)pentyl)-2-fluorobenzoyl)-l-glutamic Acid (**11**)

Using the general method for the synthesis of target compounds (**1**, **11**–**14**), **34c** (0.05 g, 0.09 mmol) was used to obtain **11** (0.02 g, 45%) as a gray solid; TLC *R*_*f*_ = 0.0 (MeOH/CHCl_3_/HCl, 1:5:0.5); mp, 138.5–145.7 °C; ^1^H NMR (400 MHz, DMSO-*d*_6_) δ: 8.42 (d, *J* = 9.0 Hz, 1H, exch., −NH), 7.52 (d, *J* = 15.6 Hz, 1H, Ar), 7.17 (d, *J* = 2.8 Hz, 1H, Ar), 7.11 (t, *J* = 25.0 Hz, 2H, Ar), 5.79 (bs, 2H, exch., −NH_2_), 5.87 (d, *J* = 2.8 Hz, 1H, Ar), 4.39 (m, *J* = 21.9 Hz, 1H, −CH), 4.20 (d, *J* = 14.1 Hz, 2H, −CH_2_−), 2.59 (t, *J* = 15.7 Hz, 2H, −CH_2_−), 2.38–2.32 (m, 2H, −CH_2_−), 2.12–1.91 (m, 2H, −CH_2_−), 1.73 (p, *J* = 7.2 Hz, 2H, −CH_2_−), 1.57 (p, *J* = 7.7 Hz, 2H, −CH_2_−), 1.23 (m, *J* = 51.8 Hz, 2H, −CH_2_−). HPLC analysis: *t*_R_, 10.833 min; purity, 97.133% at 250 nm. MS calculated for C_23_H_26_FN_5_O_6_ [M + H]^+^, 488.49. Found: 489.

##### (4-(4-(2-Amino-4-oxo-3,4-dihydro-5*H*-pyrrolo[3,2-*d*]pyrimidin-5-yl)butyl)-2,6-difluorobenzoyl)-l-glutamic Acid (**12**)

Using the general method for the synthesis of target compounds (**1**, **11**–**14**), **34d** (0.05 g, 0.09 mmol) was used to obtain **12** (0.01 g, 47%) as a white solid; TLC *R*_*f*_ = 0.0 (MeOH/CHCl_3_/HCl, 1:5:0.5); mp, 141.5–147.3 °C; ^1^H NMR (400 MHz, DMSO-*d*_6_) δ: 10.55 (s, 2H, exch., 2-COOH), 8.93 (d, *J* = 7.9 Hz, 1H, exch., −NH, Ar), 7.21 (d, *J* = 2.8 Hz, 1H, Ar), 7.00 (d, 2H, Ar), 5.89 (d, *J* = 2.8 Hz, 1H, −CH, Ar), 5.80 (s, 2H, exch., −NH_2_), 4.39 (ddd, *J* = 9.7, 7.9, 4.8 Hz, 1H, −CH), 4.25 (t, *J* = 6.8 Hz, 2H, −CH_2_−), 2.61 (t, *J* = 7.6 Hz, 2H, −CH_2_−), 2.40–2.25 (m, 2H, −CH_2_−), 2.04 (dtd, *J* = 13.0, 7.9, 4.9 Hz, 1H, 1^st^ H of −CH_2_−), 1.87–1.73 (m, 1H, 2^nd^ H of −CH_2_−), 1.71 (p, *J* = 7.0 Hz, 2H, −CH_2_−), 1.53–1.41 (m, 2H, −CH_2_−). ^13^C NMR (400 MHz, DMSO-*d*_6_) δ: 174.18, 173.19, 160.48, 160.33, 157.92, 154.99, 151.14, 131.29, 128.21, 121.19, 112.12, 111.92, 99.89, 52.06, 47.72, 34.42, 31.04, 30.37, 27.68, 26.63. HPLC analysis: *t*_R_, 12.123 min; purity, 98.962% at 290 nm. HRMS calculated for C_22_H_23_F_2_N_5_O_6_ [M + H]^+^, 492.1689. Found: 492.1692.

##### (4-(4-(2-Amino-4-oxo-3,4-dihydro-5*H*-pyrrolo[3,2-*d*]pyrimidin-5-yl)butyl)-3-fluorothiophene-2-carbonyl)-l-glutamic Acid (**14**)

Using the general method for the synthesis of target compounds (**1**, **11**–**14**), **34e** (0.05 g, 0.09 mmol) was used to obtain **14** (0.02 g, 45%) as a white solid; TLC *R*_*f*_ = 0.0 (MeOH/CHCl_3_/HCl, 1:5:0.5); mp, 138.5–145.7 °C; ^1^H NMR (500 MHz, DMSO-*d*_6_) δ: 10.97 (s, 2H, exch., 2-COOH), 7.96 (dd, *J* = 7.7, 3.5 Hz, exch., −NH), 7.48 (d, *J* = 4.3 Hz, 1H, Ar), 7.21 (d, *J* = 3.0 Hz, 1H, Ar), 5.89 (d, *J* = 2.9 Hz, 1H, Ar), 5.86 (s, 2H), 4.37 (td, *J* = 8.3, 4.6 Hz, 1H, −CH), 4.25 (t, *J* = 6.7 Hz, 2H, −CH_2_−), 2.32 (t, *J* = 7.4 Hz, 2H, −CH_2_−), 2.10 (dt, *J* = 13.7, 6.3 Hz, 1H, 1^st^ H of −CH_2_−), 2.02–1.91 (m, 1H, 2^nd^ H of −CH_2_−), 1.75 (p, *J* = 7.3 Hz, 2H, −CH_2_−), 1.47 (p, *J* = 7.5 Hz, 2H, −CH_2_−). ^13^C NMR (400 MHz, DMSO-*d*_6_) δ: 174.43, 173.37, 159.68, 154.88, 153.16, 151.12, 131.93, 131.70, 131.31, 117.56, 112.03, 99.73, 52.30, 47.78, 31.21, 30.66, 26.38–26.11 (m). HPLC analysis: *t*_R_, 12.310 min; purity, 100% at 255 nm. HRMS calculated for C_20_H_22_FN_5_O_6_S [M + H]^+^, 480.1348. Found: 480.1349.

#### General Method for the Synthesis of Target Compounds (**5**–**8**)

To a solution of ethyl 3-amino-1*H-*pyrrole-2-carboxylate hydrochloride (0.5 g, 3.24 mmol) in dry DMF (10 mL) was slowly added NaH (0.17 g, 7.1 mmol) under nitrogen at rt. The resulting mixture was stirred for about 15 min when there was no more gas evolved, and then the appropriate iodide **19a**–**b**, **23**, or **27** (1 equiv) was added. The reaction mixture was stirred at rt for 4 h, and DMF was evaporated at elevated temp. to offer a gummy residue **32f**–**i**, which was used for the next step without purification. The gummy residue was dissolved in MeOH (10 mL), and 1,3-bis(methoxycarbonyl)-2-methyl-2-thiopseudourea (0.7 g, 3.3 mmol) was added followed by AcOH (1.0 g, 15 mmol). The mixture was stirred at room temp. overnight and became a thick paste. NaOMe in MeOH (25%) (7 mL, 22 mmol) was added, and stirring was continued at rt overnight. The mixture was neutralized with AcOH, and the methanol was removed under reduced pressure. To the residue was added water (20 mL), and the pH was adjusted to 10–11 by adding NH_3_·H_2_O. The solid was collected by filtration and washed well with water. The resulting solid was added to 1 N NaOH (2 mL), and the mixture was heated at 55 °C for 3 h. The mixture was cooled and acidified using 1 N HCl. The precipitate was collected and dried overnight under reduced pressure to obtain crude **33f**–**i**. To a solution of crude **33f**–**i** in anhyd. DMF (10 mL) was added *N*-methylmorpholine (73 mg, 0.72 mmol) and 2-chloro-4,6*-*dimethoxy-1,3,5-triazine (127 mg, 0.72 mmol). The resulting mixture was stirred at rt for 2 h. To this mixture were added *N*-methylmorpholine (73 mg, 0.72 mmol) and l-glutamate diethyl ester hydrochloride (144 mg, 0.6 mmol). The reaction mixture was stirred for an additional 12 h at rt, and the solvent was evaporated to obtain crude **34f**–**i**. To a solution of crude **34f**–**i** was added 2 mL of 1 N NaOH. The reaction mixture was stirred for 1 h at rt, and the disappearance of the starting material was followed with TLC. The mixture was acidified to pH 2–3 using 1 N HCl to obtain pure target compounds (**5**–**8**) as precipitated residues on filtration.

##### (4-(3-(2-Amino-4-oxo-3,4-dihydro-5*H*-pyrrolo[3,2-*d*]pyrimidin-5-yl)ethoxy) benzoyl)-l-glutamic Acid (**6**)

Using the general method for the synthesis of target compounds (**5**–**8**), **19a** (1 g, 3.12 mmol) was used to obtain **33f** (0.3 g, crude 27%), which was taken directly to the next step. Compound **33f** (120 mg, 0.38 mmol) was used to obtain **34f** (90 mg, crude 47%) as a gray semisolid. Compound **34f** (0.10 g, 0.2 mmol) was used to obtain **6** (0.04 g, 45%) as a white solid, which was purified and separated using preperative HPLC. TLC *R*_*f*_ = 0.0 (MeOH/CHCl_3_/HCl, 1:5:0.5); mp, 216.2–223.7 °C; ^1^H NMR (400 MHz, DMSO-*d*_6_) δ: 12.25 (s. 2H, exch., −COOH), 8.50 (d, *J* = 7.7 Hz, 1H, exch., −NH), 7.85 (d, *J* = 8.9 Hz, 2H, Ar), 7.28 (d, *J* = 2.7 Hz, 1H, Ar), 7.00 (d, *J* = 8.9 Hz, 2H, Ar), 5.93 (s, 2H, exch., 2-NH_2_), 4.62 (t, *J* = 5.5 Hz, 2H, −CH_2_−), 4.40–4.32 (m, 3H, −CH– and −CH_2_−), 2.35 (t, *J* = 7.5 Hz, 2H, −CH_2_−), 2.11–1.88 (m, 2H, −CH_2_−). ^13^C NMR (400 MHz, DMSO-*d*_6_) δ: 174.52, 174.07, 166.31, 161.00, 151.41, 148.99, 148.58, 132.17, 129.77, 128.21, 126.81, 121.18, 114.43, 111.99, 100.13, 52.41, 47.38, 31.03, 26.45. Anal. Calcd. for C_20_H_21_N_5_O_7_ 1.68 H_2_O: C, 54.20; H, 4.80; N, 15.80. Found: C, 50.66; H, 4.99; N, 14.86. HPLC analysis: *t*_R_, 9.777 min; purity, 98.195% at 260 nm. HRMS calculated for C_20_H_2__1_N_5_O_7_ [M + H]^+^, 444.1504. Found: 444.1513.

##### (4-(3-(2-Amino-4-oxo-3,4-dihydro-5*H*-pyrrolo[3,2-*d*]pyrimidin-5-yl)propoxy)benzoyl)-l-glutamic Acid (**5**)

Using the general method for the synthesis of target compounds (**5**–**8**), **19b** (1 g, 3.13 mmol) was used to obtain **33g** (0.30 g, crude 27%) as a white semisolid. Compound **33g** (130 mg, 0.41 mmol) was used to obtain **34g** (110 mg, crude 53%) as a gray semisolid. Compound **34g** (0.10 g, 0.2 mmol) was used to obtain **5** (0.05 g, 56%) as a white solid; TLC *R*_*f*_ = 0.0 (MeOH/CHCl_3_/HCl, 1:5:0.5); mp, 147.2–155.9 °C; ^1^H NMR (400 MHz, DMSO-*d*_6_) δ: 12.09 (s. 2 H, exch., −COOH), 8.46 (d, *J* = 7.9 Hz, 1H, exch., −NH), 7.85 (d, *J* = 8.4 Hz, 2H, Ar), 7.20 (d, *J* = 2.9 Hz, 1H, Ar), 6.96 (d, *J* = 8.6 Hz, 2H, Ar), 6.21 (s, 2H, exch., 2-NH_2_), 5.93 (d, *J* = 2.8 Hz, 1H, Ar), 4.41–4.35 (m, 3H, −CH– and −CH_2_−), 3.94 (t, 2H, −CH_2_−), 2.35 (t, *J* = 7.5 Hz, 2H, −CH_2_−), 2.21 (m, 2H, −CH_2_−), 1.95 (m, 2H, −CH_2_−). ^13^C NMR (400 MHz, DMSO-*d*_6_) δ: 174.35, 174.06, 166.46, 161.33, 154.42, 151.15, 148.98, 131.84, 129.74, 121.18, 114.34, 111.78, 99.06, 52.32, 45.51, 42.55, 30.92 (d, *J* = 11.9 Hz), 26.37. Anal. Calcd. for C_21_H_23_N_5_O_7_ 0.78 HCl: C, 52.86; H, 5.20; N, 14.00. Found: C, 52.73; H, 5.086; N, 14.40. HPLC analysis: *t*_R_, 9.996 min; purity, 95.694% at 260 nm. HRMS calculated for C_21_H_23_N_5_O_7_ [M + H]^+^, 458.1670. Found: 458.1672.

##### (4-((3-(2-Amino-4-oxo-3,4-dihydro-5*H*-pyrrolo[3,2-*d*]pyrimidin-5-yl)propyl)thio)benzoyl)-l-glutamic Acid (**7**)

Using the general method for the synthesis of target compounds (**5**–**8**), **23** (1 g, 3.57 mmol) was used to obtain **33h** (0.28 g, crude 23%) as a white solid. Compound **33h** (150 mg, 0.45 mmol) was used to obtain **34h** (100 mg, crude 43%) as a gray semisolid; Compound **34h** (0.1 g, 0.03 mmol) was used to obtain **7** (0.04 g, 50%) as a white solid; TLC *R*_*f*_ = 0.0 (MeOH/CHCl_3_/HCl, 1:5:0.5); mp, 63.3–67.9 °C; ^1^H NMR (400 MHz, DMSO-*d*_6_) δ: 12.12 (s, br, 2H, exch., −COOH), 8.71 (d, *J* = 7.8 Hz, 1H, exch., −NH), 7.77 (d, *J* = 8.2 Hz, 2H, Ar), 7.27 (d, *J* = 8.2 Hz, 2H, Ar), 7.20 (d, *J* = 2.8 Hz, 1H, Ar), 5.97 (s, 2H, exch., 2-NH_2_), 5.90 (d, *J* = 2.8 Hz, 1H, Ar), 4.31 (m, 3H, −CH and −CH_2_−), 2.87 (t, *J* = 7.5 Hz, 2H, −CH_2_−), 2.53 (t, *J* = 7.6 Hz, 2H, −CH_2_−), 1.96–2.08 (m, 4 H, −CH_2_−). Anal. Calcd. for C_21_H_23_N_5_O_6_S 0.70 CH_3_OH 1.6 HCl: C, 47.02; H, 4.98; N, 12.64; S, 5.79. ^13^C NMR (151 MHz, DMSO-*d*_6_) δ: 174.73, 173.40, 165.50, 154.59, 151.11, 147.66, 140.32, 131.19, 130.79, 127.87, 126.23, 111.67, 99.48, 52.56, 47.01, 31.63, 30.47, 27.69, 26.58. Found: C, 47.05; H, 4.86; N, 12.47; S, 5.93. HPLC analysis: *t*_R_, 10.436 min; purity, 96.411% at 260 nm. HRMS calculated for C_21_H_23_N_5_O_6_S [M + H]^+^, 474.1442. Found: 474.1442.

##### (4-((3-(2-Amino-4-oxo-3,4-dihydro-5*H*-pyrrolo[3,2-*d*]pyrimidin-5-yl)propyl)amino)benzoyl)-l-glutamic Acid (**8**)

Using the general method for the synthesis of target compounds (**5**–**8**), compound **27** (1 g, 3.13 mmol) was used to obtain **33i** (0.35 g, crude 34%) as a white solid. Compound **33i** (120 mg, 0.38 mmol) was used to obtain crude **34i** (90 mg, crude 47%) as a gray semisolid. Compound **34i** (0.10 g, 0.2 mmol) was used to obtain **8** (0.056 g, 34%) as a white solid; TLC *R*_*f*_ = 0.0 (MeOH/CHCl_3_/HCl, 1:5:0.5); mp, 76.7–83.9 °C; ^1^H NMR (400 MHz, DMSO-*d*_6_) δ: 12.02 (s, 2H, −COOH), 8.14 (d, *J* = 7.9 Hz, 1H, exch., −NH), 7.66 (d, *J* = 8.3 Hz, 2H, Ar), 7.28 (d, *J* = 2.6 Hz, 1H, Ar), 6.52 (d, *J* = 8.4 Hz, 2H, Ar), 6.34 (s, 2H, exch., 2-NH_2_), 6.26 (s, 1H, -exch., −NH−), 5.97 (d, *J* = 2.7 Hz, 1H, Ar), 4.38–4.31 (m, 3H, −CH and −CH_2_−), 2.98 (t, *J* = 6.5 Hz, 2H, −CH_2_−), 2.33 (t, *J* = 7.5 Hz, 2H, −CH_2_−), 2.10–1.88 (m, 4 H, −CH_2_−). Anal. Calcd. for C_21_H_24_N_6_O_6_ 0.5 CH_3_OH 0.8 HCl: C, 51.47; H, 5.40; N, 16.74. Found: C, 51.50; H, 5.26; N, 16.66. ^13^C NMR (400 MHz, DMSO-*d*_6_) δ: 174.43, 174.35, 166.92, 154.52, 151.77, 151.16, 131.84, 129.48, 128.33, 120.92, 111.71, 111.05, 99.03, 52.18, 46.17, 30.90, 26.45. HPLC analysis: *t*_R_, 9.778 min; purity, 99.275% at 260 nm. HRMS calculated for C_21_H_24_N_6_O_6_ [M + H]^+^, 457.1830. Found: 460.1835.

#### Molecular Modeling and Computational Studies

Molecular modeling was performed for all analogues with the X-ray crystal structure of human SHMT2 with **1** (PDB ID: 8FJU) and human GARFTase with **3** (PDB ID: 8FJX), and the published crystal structure of human AICARFTase (PDB ID: 1P4R).^37^ For docking studies in SHMT1,^19^ the X-ray crystal structure of rabbit (*O. cuniculus*) cytosolic SHMT1 crystallized with 5-formyl THF tri-glutamate (PDB ID: 1LS3)^19^ was used. To optimize the transport of our designed analogues, we used the X-ray crystal structures of human FRα in complex with our novel antifolate *N*-(4-{[2-(2-amino-4-oxo-4,7-dihydro-3*H*-pyrrolo[2,3-*d*]pyrimidin-6-yl)ethyl]amino}benzene-1-carbonyl)-l-glutamic acid (**10**) (PDB ID: 5IZQ)^38^ and human FRβ in complex with PMX (PDB ID: 4KN2).^39^ Docking used the induced-fit docking with Maestro12.1. (Maestro, Schrödinger, LLC, New York, NY, 2021). The docking scores of the analogues are reported in Table S1 (Supplemental Information).

#### Reagents for Biological Studies

Folic acid was purchased from Sigma Chemical Co. (St. Louis, MO). Leucovorin [(6*R*,*S*)5-formyl tetrahydrofolate] was provided by the Drug Development Branch, National Cancer Institute, Bethesda, MD. The sources of the classical antifolate drugs were as follows: MTX (Drug Development Branch, National Cancer Institute, Bethesda, MD); PDX [*N*-(4-{1-[(2,4-diaminopteridin-6-yl)methyl]but-3-yn-1-yl}benzoyl)-l-glutamic acid] (Allos Therapeutics, Westminster, CO); and PMX [*N*-{4-[2-(2-amino-3,4-dihydro-4-oxo-7*H*-pyrrolo[2,3-*d*]pyrimidin-5-yl)ethyl]benzoyl}-l-glutamic acid] (Alimta) (Eli Lilly and Co., Indianapolis, IN). **AGF94** [(*S*)-2-({5-[3-(2-amino-4-oxo-4,7-dihydro-3*H*-pyrrolo[2,3-*d*]pyrimidin-6-yl)-propyl]-thiophene-2-carbonyl}-amino)-pentanedioic acid] was synthesized as previously described.^69^ Other chemicals were obtained from commercial sources in the highest available purity.

#### Cell Lines and Assays of Antitumor Drug Activities

The RFC-, PCFT-, and FR-null MTXRIIOua^R^2–4 (R2), and RFC- (PC43-10), PCFT- (R2/PCFT4), FRα- (RT16), and FRβ- (D4) expressing CHO sublines were previously described.^62−64,76^ HPAC pancreatic cancer cells were generously provided by Dr. Yubin Ge (Karmanos Cancer Institute, Detroit, MI). Human pancreatic normal epithelial (HPNE) cells^67^ were a gift from M. Oulette (University of Nebraska Medical Center, Omaha, NE). KB human nasopharyngeal carcinoma cells were purchased from the American Type Culture Collection (Manassas, VA). All human cell lines were validated by STR analysis by Genetica DNA Laboratories (Burlington, NC). Cell lines were tested for *Mycoplasma* by PCR using a *Mycloplasma* testing kit (Venor GeM *Mycoplasma* Detection Kit, Sigma). Frozen stocks were generated from authenticated *Mycoplasma*-free cultures.

The CHO cells were cultured in α-minimal essential medium (MEM) supplemented with 10% bovine calf serum (Fisher Scientific), penicillin (1000 U/mL)/streptomycin (1000 μg/mL), and 2 mM l-glutamine at 37 °C with 5% CO_2_. All of the R2 transfected cells (PC43-10, RT16, R2/PCFT4) were cultured in complete α-MEM media plus 1 mg/mL G418. Prior to the cytotoxicity assays (see below), RT16 and D4 cells were cultured for 3 days in complete folate-free RPMI 1640 (without added folate), and R2/PCFT4 and R2 cells were cultured in complete folate-free RPMI 1640 supplemented with dialyzed fetal bovine serum (Fisher Scientific) and 25 nM leucovorin plus 1 mg/mL G418. KB cells were routinely cultured in folate-free RPMI 1640 medium, supplemented with 10% fetal bovine serum (Fisher Scientific), penicillin-streptomycin solution, and 2 mM l-glutamine at 37 °C with 5% CO_2_.

For growth inhibition studies, cells (CHO, KB) were plated in 96-well dishes (∼2500–5000 cells/well, total volume of 200 μL medium) over a range of antifolate concentrations.^69,77^ The medium was folate-free RPMI 1640 with 10% dialyzed serum and antibiotics supplemented with 25 nM leucovorin for experiments with R2, PC43-10, and R2/PCFT4 cells. For experiments with RT16, D4, and KB cells, cells were cultured in folate-free RPMI medium with 10% dialyzed fetal bovine serum and antibiotics; the medium was supplemented with 2 nM leucovorin. FR-mediated drug uptake in these experiments was established by adding 200 nM folic acid to parallel incubations, conditions which had no significant impact on the growth inhibition with R2/PCFT4 cells. Incubations were routinely for up to 96 h. Viable cells (reflecting cell viability) were assayed with Cell Titer Blue reagent (Promega, Madison, WI), with fluorescence measured (590 nm emission, 560 nm excitation) with a fluorescence plate reader. Relative fluorescence data were analyzed for calculations of IC_50_s, corresponding to the drug concentrations that resulted in 50% loss of cell proliferation.

For some experiments with KB tumor cells, cells were cultured in serine-, glycine-, and folate-free RPMI 1640 (Fisher Scientific), supplemented with 10% dialyzed fetal bovine serum, antibiotics, 25 nM leucovorin, and 2 mM l-glutamine. *In vitro* growth inhibition was measured (above), without additions, or in the presence of folic acid (200 nM), or thymidine (10 μM), adenosine (60 μM), 5-aminoimidazole-4-carboxamide (AICA) (320 μM), and/or glycine (130 μM) to establish the inhibitory effects of the antifolate drugs on *de novo* thymidylate synthase versus *de novo* purine nucleotide biosynthesis (GARFTase and AICARFTase) or mitochondrial C1 metabolism at SHMT2.^36,63,64,68,69^ For *de novo* purine biosynthesis, the AICA protection experiments were used to distinguish inhibitory effects at GARFTase from those at AICARFTase.^63,64,68,69^ SHMT2 inhibition was reflected as protection with adenosine combined with glycine as previously described.^36^

### Enzyme Expression and Purification

The formyltransferase domain of human GARFTase (residues 808–1010) with an N-terminal hexahistidine(His_6_)-tag and N-terminal His_6_-tagged full-length human ATIC were expressed in Rosetta (DE3)pLysS cells.^36,78^ The cells were grown at 37 °C in 1 L cultures of LB media containing 100 mg/mL ampicillin and 34 mg/mL chloramphenicol until OD_600_ reached 0.6. Protein expression was induced by the addition of isopropyl β-D1-thiogalactopyranoside (IPTG, 500 μM). The cells were then grown for 16–18 h at 20 °C. The cells were then pelleted and resuspended in 40 mL of 25 mM Tris-HCl, pH 7.5, 300 mM NaCl, 5 mM β-mercaptoethanol (β-Me), 10 mM CaCl_2_, 10 mM MgCl_2_, 40 mg lysozyme, and 8 U DNAse I (Sigma); the cells were lysed by emulsification.

His-GARFTase was purified by Ni-NTA chromatography (Qiagen, Valencia, CA) on an ÄKTA FPLC (GE Healthcare).^36,78^ Five column volumes of 25 mM Tris-HCl, pH 8, 300 mM NaCl, 10% glycerol, and 5 mM β-Me were used to wash the Ni-NTA column. The protein was eluted over 10 column volumes with a 0–75% gradient of 300 mM imidazole, 25 mM Tris-HCl, pH 8, 300 mM NaCl, 10% glycerol, and 5 mM β-Me. Size exclusion chromatography on a Superdex 75 16/60 (GE Healthcare) column was used for final purification. The final buffer conditions for His-GARFTase were 25 mM Tris-HCl, pH 8, 10 mM β-Me, and 300 mM NaCl. For assays, His-GARFTase was stored at −80 °C at 90 μM protein concentration in 20% glycerol.

His-ATIC was purified by Ni-NTA chromatography (Gold Biotechnology).^36^ The cell lysate was passed over the column twice and washed with 5 column volumes of 10 mM imidazole, 25 mM Tris-HCl, pH 7.5, 300 mM NaCl, and 5 mM β-Me, and 10 column volumes of 25 mM imidazole, 25 mM Tris-HCl, pH 7.5, 300 mM NaCl, and 5 mM β-Me. His-ATIC was eluted in 1 column volume fractions, 5 times with buffer containing 300 mM imidazole, 25 mM Tris-HCl, pH 7.5, 300 mM NaCl, and 5 mM β-Me. Size exclusion chromatography on a Superdex 200 16/60 (GE Healthcare) column was used for final purification using 20 mM Tris-HCl, pH 7.5, 150 mM NaCl, 50 mM KCl, 5 mM EDTA, and 5 mM dithiothreitol. The final purified enzyme was stored at 4 °C at 150 μM for up to 6 months, or at −80 °C in 20% glycerol at 100 μM for a longer term.

Truncated SHMT2 (residues 30–504) with an N-terminal His_6_-tag, full-length SHMT1 with an N-terminal His_6_-tag, and full-length MTHFD2 with an N-terminal His_6_-tag were expressed in Rosetta (DE3)pLysS cells.^36^ Cultures were grown in 1 L LB media with 100 mg/mL ampicillin and 34 mg/mL chloramphenicol added at 37 °C. Cells were grown until they reached an OD_600_ of 0.6, induced with 500 μM IPTG and grown for an additional 16–18 h at 18 °C. The cells were then pelleted and resuspended in 25 mL of resuspension buffer, which included 40 mg of lysozyme and 8 U DNAse for lysis. The resuspended cells were incubated on ice for 15 min and lysed by emulsification.

Purification of the three enzymes was performed by Ni-NTA chromatography (Gold Biotechnology), as described for His-ATIC. After purification by Ni-NTA, pyridoxal-5′-phosphate (PLP) was added to SHMT1 or SHMT2 in 3-fold molar excess and allowed to incubate for 16–18 h. Final purification of SHMT enzymes was completed with size exclusion chromatography with a Superdex 200 16/60 column (GE Healthcare). Buffer containing 20 mM sodium phosphate, pH 7.5, 100 mM KCl, 0.2 mM EDTA, and 5 mM β-Me was used for the chromatography and selection of PLP-loaded SHMT enzyme was performed by monitoring absorbance at 435 nm. (the wavelength of the Lys-PLP covalent bond). His-MTHFD2 was buffer-exchanged using a G25 Sephadex desalting column (GE Healthcare) for final purification using 50 mM Tris-HCl, pH 7.5, 250 mM NaCl, 5 mM magnesium chloride, 5% glycerol, and 0.5 mM Tris(2-carboxyethyl)phosphine (TCEP) for protein assays.

### Crystallization and Structure Determination of Human GARFTase and Human SHMT2

The purified human GARFTase (residues 808–1010) with a noncleavable C-terminal His_6_-tag was buffer-exchanged into 25 mM Tris-HCl, pH 8.0, 200 mM NaCl, and 0.6 mM TCEP. The protein was then concentrated to 10 mg/mL and incubated in a 3-fold ratio with α,β-GAR in excess at 4 °C for 30 min in the presence of an inhibitor. Crystals were formed by hanging drop plates containing 1 μL of protein-ligand solution, 0.8 μL of crystal condition, and 0.2 μL of 9 mM *N*-decyl β-d-thiomaltoside (Hampton Research, Aliso Viejo, CA), which was incubated over 0.5 mL crystallant containing 0.1 M Tris-HCl, pH 7.5, 330 mM NaCl, 16–21% poly(ethylene glycol) (PEG) 4000, and 2% PEG 400. After a few days, cube-shaped crystals formed and were cryo-protected with a stepwise transfer into crystallant with 35% PEG 4000, then flash-frozen by immersion into liquid nitrogen.^79^

Purified human His_6_-SHMT2 loaded with PLP was crystallized in bulk solution. The protein was stored in buffer containing 20 mM sodium phosphate, pH 7.5, 100 mM NaCl, 0.2 mM EDTA, and 0.5 mM TCEP at 10 to 20 μM. After storing at 4 °C for 5–7 days, rod-shaped crystals formed. Crystals were soaked with serine and inhibitor at a 5:5:1 ratio of serine:inhibitor:His-SHMT2 and transferred to crystallant containing 35% glycerol, 100 μM serine, and 100 μM inhibitor for cryo-protection before being plunged into liquid nitrogen.

Data collection was performed at the Advanced Light Source beamline 4.2.2 using the Taurus CMOS detector at Lawrence Berkeley National Laboratory. Data sets for GARFTase were processed in space group P3_2_2 (XDS).^80,81^ Protein Data Bank (PDB) entry 1J9F with waters and ligands removed was used as the molecular replacement search model (PHE-NIX).^82^ Data sets for SHMT2 were processed to space group P6_5_22 (XDS). Molecular replacement was performed using PDB ID: 5V7I with waters and ligands removed as the search model.^83^ Coot was used for model building and Phenix was used for structure refinement.^82,84^

### *In Vitro* Enzymatic Assays and *K_i_* Determinations

Inhibition of GARFTase activity was evaluated by monitoring the conversion of 10-formyl THF to THF by absorbance at 298 nm in the presence of an inhibitor.^36,48^ The GARFTase inhibition assays utilized 40 μM 10-formyl THF, 50 nM His-GARFTase, 15 μM α,β-GAR, and a range of inhibitor concentrations (final concentrations) in assay buffer containing 25 mM Tris-HCl, pH 8, 300 mM NaCl, and 5 mM β-Me at 37 °C.^36^ AICARFTase activity was also measured by monitoring the conversion of 10-formyl THF to THF by absorbance at 298 nm in the presence of inhibitor.^36^ The AICARFTase inhibition assays utilized 50 μM 10-formyl THF, 100 nM His-ATIC, 50 μM ZMP, and a range of inhibitor concentrations in assay buffer containing 32.6 mM Tris-HCl, pH 7.5, 25 mM KCl, and 5 mM β-Me at 25 °C.^36^ MTHFD2 activities were assayed by a reaction with methylene THF and NAD^+^. NADH production was measured by fluorescence (excitation 340 nm, emission 480 nm). Reaction conditions included 100 nM MTHFD2, 5 μM methylene THF, and concentrations of small-molecule inhibitor ranging from 0.02 nM to 2000 nM; NAD^+^ (2.5 mM) was added to initiate the reaction. The reaction buffer consisted of 50 mM Tris-HCl, pH 7.5, 250 mM NaCl, 5 mM magnesium chloride, 5% glycerol, and 0.5 mM TCEP. Kinetic measurements were recorded in triplicate using a flat, black-bottom, black-walled 96-well plate (Greiner Bio-One, 655900) and a BioTek Synergy Neo2 Plate Reader. MTHFD2 Inhibition was not detected with any of the small-molecule inhibitors in this work. To assay SHMT1 or SHMT2 (SHMT1/2) enzymes, a coupled reaction with His-MTHFD2 in 200-fold molar excess was used where the production of NADH was monitored by fluorescence with excitation at 360 nm and emission at 470 nm at 25 °C. The reactions contained 5 nM His-SHMT1/2, 50 μM THF, 20 mM serine, 10 μM His-MTHFD2, 2.5 mM NAD^+^, and a range of inhibitor concentrations (final concentrations). The reactions were performed in buffer containing 20 mM sodium phosphate, pH 7.5, 100 mM KCl, 0.2 mM EDTA, and 5 mM β-Me. A BioTek Synergy Neo2 Plate Reader was used to record kinetic measurements in triplicate in a UV-transparent 96-well plate (Costar 3635) for GARFTase and AICARFTase assays and a black-well, black-bottom 96-well plate (Corning 3603). To calculate IC_50_ values for each inhibitor, initial velocities were graphed as a function of inhibitor concentrations and fit with a three-parameter nonlinear regression (GraphPad Prism 8.0). The equation [*K_i_* = IC_50_/([*S*]/*K*_M_ + 1)] was used to convert IC_50_’s to *K_i_* values. *K*_M_ values were calculated as 84.8 μM for His-GARFTase, 100 μM for His-ATIC, 63 μM for SHMT1, and 108 μM for SHMT2.^36^

#### Statistics

All data reflect at least three biological replicates unless noted otherwise. All statistical comparisons were performed using unpaired *t*-tests after data were transformed to meet normality assumptions, and no *p*-value adjustments were made for multiple comparisons. A log_2_ transformation was used for data with positive values and, when data included zero values, a square root transformation was used. For depicting data in plots, all data were summarized with mean values and standard deviations using data without transformation.

## Data Availability

Atomic coordinates and structure factors for the reported crystal structures have been deposited in the Protein Data Bank (8FJT, 8FJU, 8FJV, 8FJW, 8FJX, 8FJY); see Table S2, Supporting Information. The authors will release the atomic coordinates and experimental data upon article publication.

## Supporting Information Available

Spectral and other characterization data for compounds **5**–**8** and **12**–**14** including ^1^H NMR, HPLC, HRMS (Tables S1–S2) (Figures S1–S11) (PDF)
Molecular formula string and the associated biological data (CSV)

## Abbreviations Used

δ: chemical shift in parts per million downfield from tetramethylsilane
μ: micro
°C: degrees Celsius
AICA: 5-aminoimidazole-4-carboxamide
AICARFTase: 5-aminoimidazole-4-carboxamide ribonucleotide formyltransferase
anhyd: anhydrous
ATIC: 5-aminoimidazole-4-carboxamide ribonucleotide formyltransferase/inosine monophosphate cyclohydrolase
β-ME: β-mercaptoethanol
C1: one-carbon
CHO: Chinese hamster ovary
FR: folate receptor
GAR: glycinamide ribonucleotide
GARFTase: glycinamide ribonucleotide formyltransferase
HPLC: high-performance liquid chromatography
HPNE: human pancreatic normal epithelial
HRMS: high-resolution mass spectroscopy
IC_50_: half-maximum inhibitory concentration
IPTG: isopropylthio-β-galacoside
*K_i_*: inhibition constant
mol: molecular (as in mol wt)
mp: melting point
MTHFD1: 5,10-methylene tetrahydrofolate dehydrogenase 1
MTHFD2: 5,10-methylene tetrahydrofolate dehydrogenase 2
MTHFD2L: 5,10-methylene tetrahydrofolate dehydrogenase 2 like
MTX: methotrexate
MEM: minimal essential media
NAD+: nicotinamide adenine dinucleotide
NADH: reduced nicotinamide adenine dinucleotide
NADPH: reduced nicotinamide adenine dinucleotide phosphate
NMR: nuclear magnetic resonance
PCFT: proton-coupled folate transporter
PDB: Protein Data Bank
PDP: pyridoxal phosphate
PDX: pralatrexate
PEG: poly(ethylene glycol)
PMX: pemetrexed
*R*_*f*_: retention factor
*t*_R_: retention time
RFC: reduced folate carrier
rt: room temperature
ppm: part(s) per million
psi: pounds per square inch
RTX: raltitrexed
SAR: structure–activity relationships
SHMT1: serine hydroxymethyl transferase 1
SHMT2: serine hydroxymethyl transferase 2
TAMs: tumor-associated macrophages
TCEP: tris(2-chloroethyl) phosphate
temp: temperature
TLC: thin-layer chromatography
