# Supplementary material for: Structure-Based Design of Transport-Specific Multitargeted One-Carbon Metabolism Inhibitors in Cytosol and Mitochondria
Source: J Med Chem. 2023 Aug 15;66(16):11294–323. doi: 10.1021/acs.jmedchem.3c00763 (PMC10461232; doi:10.1021/acs.jmedchem.3c00763)

SUPPLEMENTAL INFORMATION

STRUCTURE-BASED DESIGN OF TRANSPORT SPECIFIC MULTI-TARGETED ONE-CARBON METABOLISM INHIBITORS IN CYTOSOL AND MITOCHONDRIA

Md. Junayed Nayeen[a,δ], Jade M. Katinas[d,δ], Tejashree Magdum[a], Khushbu Shah[a], Jennifer E. Wong[d], Carrie E. O'Connor[b], Alexandra N. Fifer[d], Adrianne Wallace-Povirk[b], Zhanjun Hou[b, c], Larry H. Matherly[b,c,¥,*], Charles E. Dann III[d,¥,*] and Aleem Gangjee[a,¥,*]

[a] Division of Medicinal Chemistry, Graduate School of Pharmaceutical Sciences, Duquesne University, Pittsburgh PA 15282

[b] Department of Oncology, Wayne State University School of Medicine, Detroit, Michigan 48201

[c] Molecular Therapeutics Program, Barbara Ann Karmanos Cancer Institute, 4100 John R, Detroit, Michigan 48201

[d] Department of Chemistry, Indiana University, Bloomington, IN 47408

δ, ¥ Equal contributions

*To whom correspondence should be addressed. gangjee@duq.edu; cedann@indiana.edu; matherly@karmanos.org

TABLE OF CONTENTS

Supplementary Tables S1-S2 ………………………………………………………… S2-S3

Supplementary Figures S1-S11 …………………………………………………… S4-S15

Spectral and Other Characterization Data for Compounds 5-8, 12-14…........... S16-S26

SUPPLEMENTAL TABLES

Table S1. Docking scores of 5-substituted pyrrolo[3,2-*d*]pyrimidine analogs in different targets.

**Compound Number**	**SHMT2** Docking Score (kcal/mol) **PDB: 8FJU**	**SHMT1** Docking Score (kcal/mol) **PDB: 1LS3**	**GARFTase** Docking Score (kcal/mol) **PDB: 5J9F**	**ATIC** Docking Score (kcal/mol) **PDB: 1P4R**	**FRα** Docking Score (kcal/mol) **PDB: 5IZQ**	**FRβ** Docking Score (kcal/mol) **PDB: 4KN2**
**1**	-10.0	-10.9	-15.4	-12.9	-21.7	-19.0
**13**	-9.5	-10.8	-8.1	-13.8	-19.2	-19.4
**14**	-9.4	-10.3	-14.4	-14.4	-20.0	-18.9
**12**	-9.5	-10.9	-15.7	-11.0	-20.5	-19.2
**5**	-8.1	-10.2	-16.5	-12.8	-17.3	-18.2
**6**	-8.5	-10.2	-13.7	-12.1	-17.3	-18.2
**7**	-8.6	-10.3	-14.4	-11.9	-19.1	-17.9
**8**	-7.5	-9.3	-16.1	-11.3	-14.5	-17.1
**15**	-9.5	-10.2	-16.0	-14.4	-19.5	-20.1
**9**	-10.2	-12.3	-13.6	-11.1	-18.7	-18.4
**16**	-9.8	-10.4	-14.6	-13.9	-18.2	-18.4
**11**	-13.3	-10.5	-16.1	-11.0	-18.5	-18.2
**2**	-10.3	-10.8	-9.6	-12.0	-18.8	-19.6
**3**	-10.0	-11.9	-15.9	-11.7	-17.2	-18.7
**4**	-9.7	-10.1	-9.3	-12.0	-17.4	-19.2

Table S2. Data collection and refinement statistics for SHMT2 and GARFTase crystal structures in complex with 5-substituted pyrrolopyrimidines and substrates PLP/PLG and β-GAR, respectively.*

	SHMT2	SHMT2	GARFTase	GARFTase	GARFTase	GARFTase
	**Compound 1**	**Compound 14**	**Compound 2**	**Compound 3**	**Compound 1**	**Compound 14**
**PDB ID**	**8FJU**	**8FJT**	**8FJY**	**8FJX**	**8FJW**	**8FJV**
**Data Collection**						
Space group	$P6_{5}22$	$P6_{5}22$	$P3_{2}21$	$P3_{2}21$	$P3_{2}21$	$P3_{2}21$
Cell dimensions						
a=b, c (Å)	157.34, 207.55	160.39, 208.75	75.48, 100.77	75.25, 100.04	74.37, 100.34	74.94, 101.01
Molecule per a.s.u.	2	2	1	1	1	1
	48.54-2.43	48.85-2.47	39.91-2.98	39.68-2.17	39.58-2.08	39.86-2.69
Resolution (Å)	2.50-2.43	2.54-2.47	3.16-2.98	2.24-2.17	2.14-2.08	2.82-2.69
$I/\sigma I$	7.2 (0.5)	9.9 (0.7)	5.0 (0.9)	11.6 (0.6)	16.0 (0.7)	8.0 (0.8)
Completeness (%)	100%	100%	100%	100%	95.60%	100%
Redundancy	12.8	12.8	10.3	10.0	7.7	10.2
R_{meas}	0.422 (4.604)	0.306 (4.180)	0.562 (2.859)	0.196 (3.680)	0.096 (1.819)	0.322 (3.037)
R_{pim}	0.116 (1.245)	0.085 (1.151)	0.174 (0.905)	0.062 (1.344)	0.033 (1.068)	0.101 (0.938)
CC1/2	0.989 (0.335)	0.995 (0.382)	0.977 (0.407)	0.997 (0.437)	0.998 (0.384)	0.993 (0.347)
**Refinement**						
	48.54-2.51	48.85-2.47	39.906-2.98	39.68-2.17	39.58-2.08	39.86-2.69
Resolution (Å)	(2.60-2.51)	(2.56-2.47)	(3.09-2.98)	(2.25-2.17)	(2.15-2.08)	(2.79-2.69)
No. reflections						
Used for refinement	52021	57003	7082	16944	18770	9498
Used for R_{free} calculation	4996	5405	645	1272	1351	933
R_{factor} (%)	23.0	24.2	20.6	20.3	20.8	19.2
R_{res} (%)	26.9	28.5	25.1	24.6	23.6	24.5
**No. atoms**						
Protein	7126	7117	1489	1523	1496	1493
Ligand	102	66	50	51	52	51
Water molecules	205	243	15	76	91	40
**B-factors** (Å^2, average)						
Overall	48.5	51.5	44.2	44.5	43.7	60.1
Protein	48.6	51.6	44.0	44.2	43.5	59.3
Ligand	54.2	60.4	51.3	54.2	55.6	92.9
r.m.s. deviations						
Bond lengths (Å)	0.002	0.002	0.008	0.004	0.002	0.008
Bond angles (°)	0.549	0.556	0.977	0.788	0.705	1.101
MolProbity clash score	6.0	6.8	3.9	2.5	1.9	4.5
MolProbity score	1.68	1.78	1.79	1.33	1.26	1.61

**Values in parentheses are for the highest resolution shell.*

SUPPLEMENTAL FIGURES

A

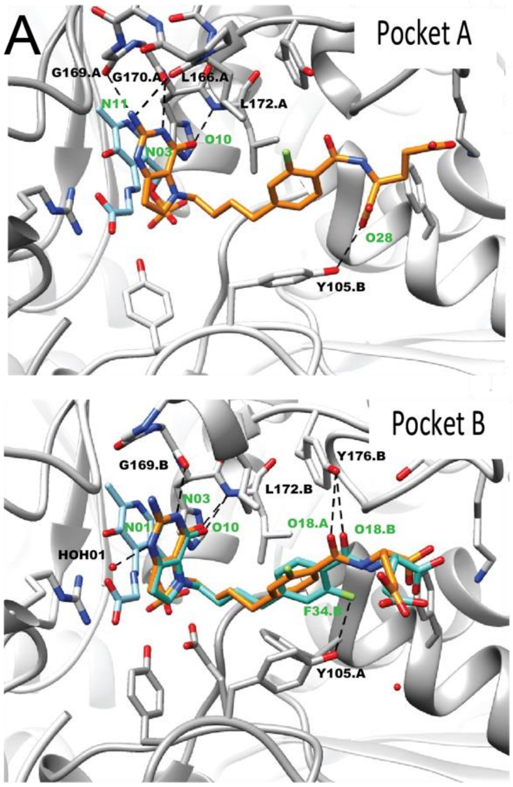

Binding Pocket	Atom 1	Atom 2	Distance (Å)
A	Gly169 O.A	Cmp1 N11	3.25
A	Gly170 O.A	Cmp1 N03	2.77
A	Leu172 N.A	Cmp1 O10	3.20
A	Tyr105 OH.B	Cmp1 O28	2.80
A	Leu166 O.A	Cmp1 N11	3.29
B	HOH01	Cmp1 N01.A/N01.B	2.93/3.04
B	Gly170 O.B	Cmp1 N03.A/N03.B	2.88/2.88
B	Leu172 N.B	Cmp1 O10.A/O10.B	3.20/3.20
B	Tyr105 OH.A	Cmp1 F34.B	3.23
B	Tyr176 OH.B	Cmp1 O18.A/O18.B	3.28/3.34

B

Pocket A - Compound 1 Electron Density

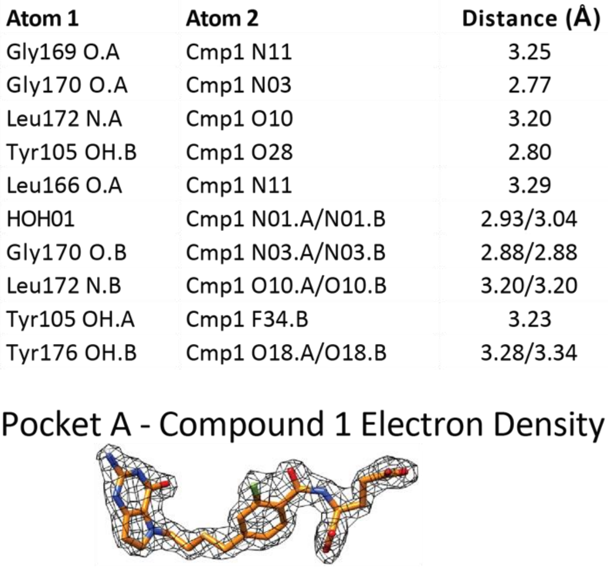

Pocket B - Compound 1 Electron Density

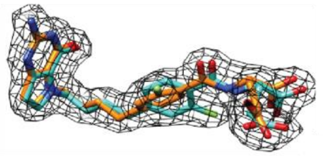

Figure S1. Crystal structure of Compound 1 bound in the folate binding pocket of PLP-loaded SHMT2. (A) Hydrogen-bond and charge-charge interactions between **Compound 1** and SHMT2 are shown as dashed lines. SHMT2 is shown in ribbon except for interacting residues, which are shown as sticks. The distances between interacting atoms in **Compound 1** and the enzyme are shown in the table. Because the binding pocket for SHMT2 contains chains A and B of the dimer, the chain is denoted after the residue atom. In pocket A, **Compound 1** is represented in orange. In pocket B where two conformations of **Compound 1** are modeled, the conformation of the molecule is denoted after the interacting atom. Conformation A, with an occupancy of 41%, is represented in orange and conformation B, with occupancy of 59%, is represented in cyan. In the two pockets, an adduct of PLP-bound to free glycine (represented in light blue) was modeled. Detailed SHMT2: inhibitor contacts for these compounds are provided in the table. **(B)** The structure is shown of **Compound 1** with the 2Fo – Fc map density in mesh contoured at 0.7 σ.

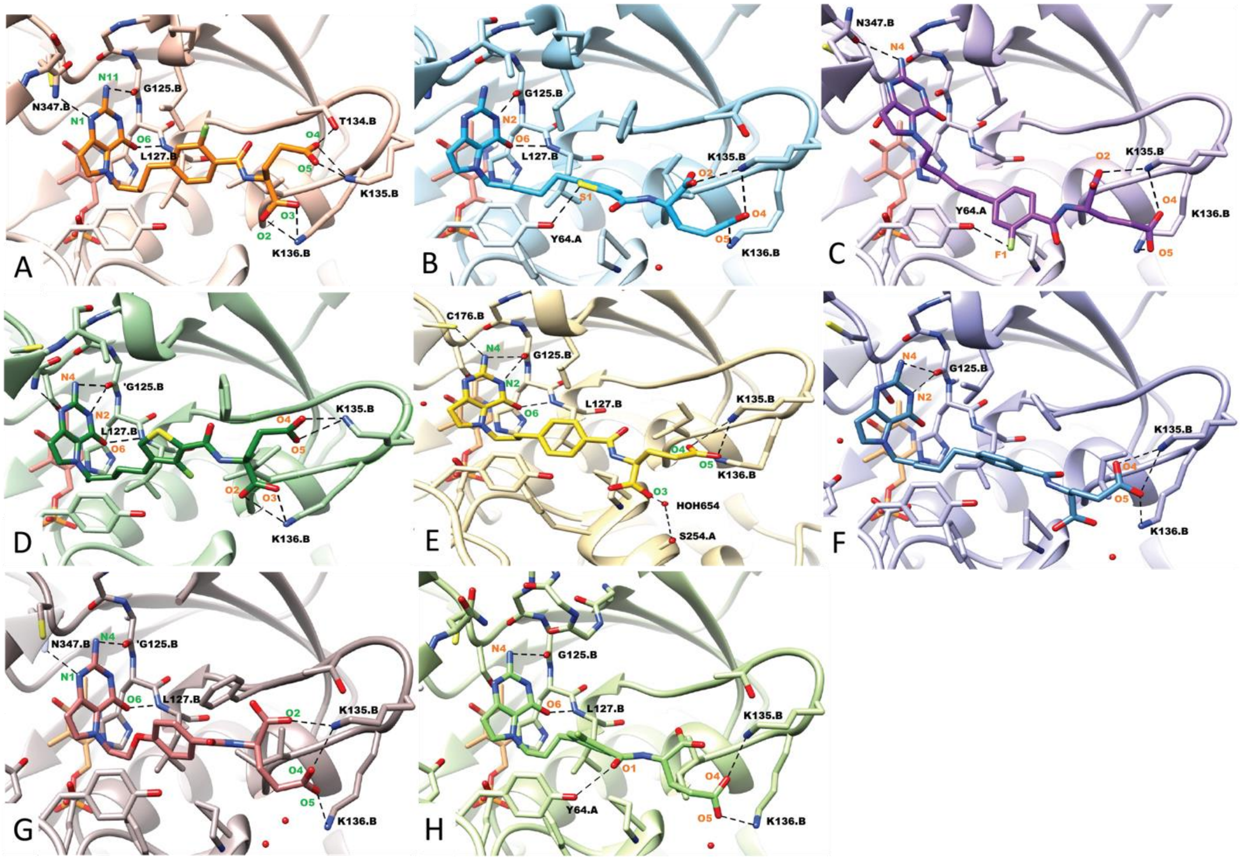

Figure S2. Molecular modeling studies with SHMT1 bound to 5-formyl THF tri-glutamate (PDB: 1LS3) (A) Docked pose of Compound 1. Protein is shown as a ribbon with interacting side-chains represented as sticks. Polar contacts are noted with dashed lines. PLP is colored in salmon in all figures. **(B)** Docked pose of Compound 3. **(C)** Docked pose of Compound 11. **(D)** Docked pose of Compound 14. **(E)** Docked pose of Compound 2. **(F)** Docked pose of Compound 9. **(G)** Docked pose of Compound 6. **(H)** Docked pose of Compound 13. Docked scores are summarized in **Table S1**.

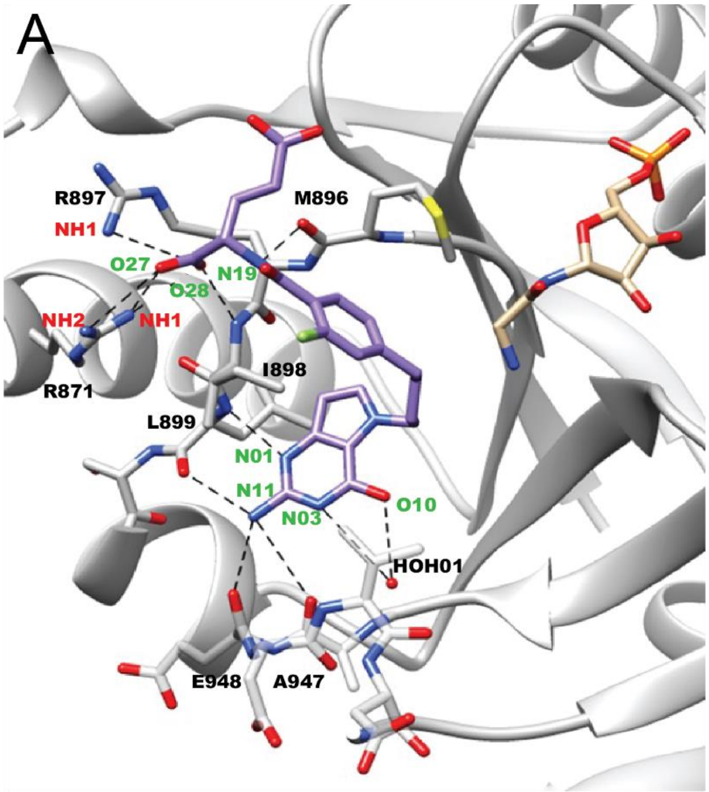

Atom 1	Atom 2	Distance (Å)
Leu899 N	Cmp1 N01	2.95
Leu899 O	Cmp1 N11	3.27
Glu948 O	Cmp1 N11	3.39
Met896 O	Cmp1 N19	3.49
Ala947 O	Cmp1 N03	3.46
Ile898 N	Cmp1 O27	2.09
HOH01	Cmp1 N03	3.83
HOH01	Cmp1 O10	3.03
Arg871 NH2	Cmp1 O27	2.63
Arg871 NH1	Cmp1 O28	3.00
Arg897 NH1	Cmp1 O28	4.54

B

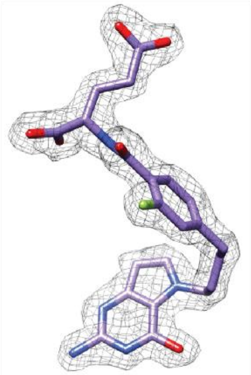

Figure S3. Crystal structure of Compound 1 bound in the folate binding pocket of GARFTase. (A) Hydrogen-bond and charge-charge interactions between **Compound 1** and GARFTase are shown as dashed lines. Atoms that participate in longer distance charge-charge interactions are labeled in red. GARFTase is shown in ribbon except for interacting side-chain and backbone atoms, which are shown as sticks. Distances between interacting atoms are shown in the table with distances greater than 3.2 Å in red. **(B)** The structure is shown of **Compound 1** with the 2Fo – Fc map density in mesh contoured at 0.7 σ.

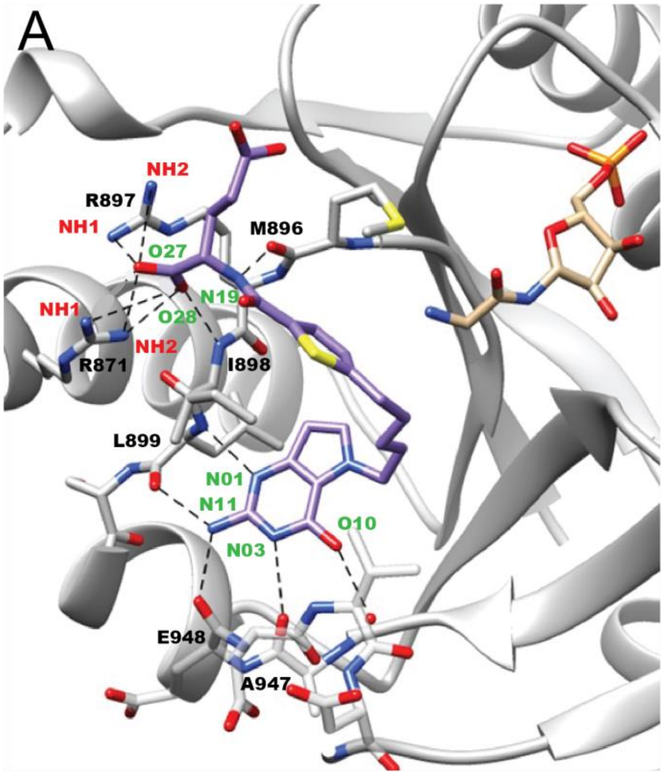

Atom 1	Atom 2	Distance (Å)
Leu899 N	Cmp3 N01	2.91
Leu899 O	Cmp3 N11	2.87
Glu948 O	Cmp3 N11	3.12
Met896 O	Cmp3 N19	2.73
Ala947 O	Cmp3 N03	2.92
Ile898 N	Cmp3 O27	2.93
HOH01	Cmp3 O10	2.77
Arg871 NH2	Cmp3 O27	2.83
Arg871 NH1	Cmp3 O28	2.83
Arg897 NH1	Cmp3 O27	2.99
Arg897 NH2	Cmp3 O28	3.56

B

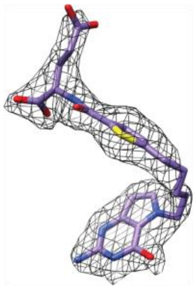

Figure S4. Crystal structure of Compound 3 bound in the folate binding pocket of GARFTase. (A) Hydrogen-bond and charge-charge interactions between **Compound 3** and GARFTase are shown as dashed lines. Atoms that participate in longer distance charge-charge interactions are labeled in red. GARFTase is shown in ribbon except for interacting side-chain and backbone atoms, which are shown as sticks. Distances between interacting atoms are shown in the table. **(B)** The structure is shown of **Compound 3** with the 2Fo – Fc map density in mesh contoured at 0.7 σ.

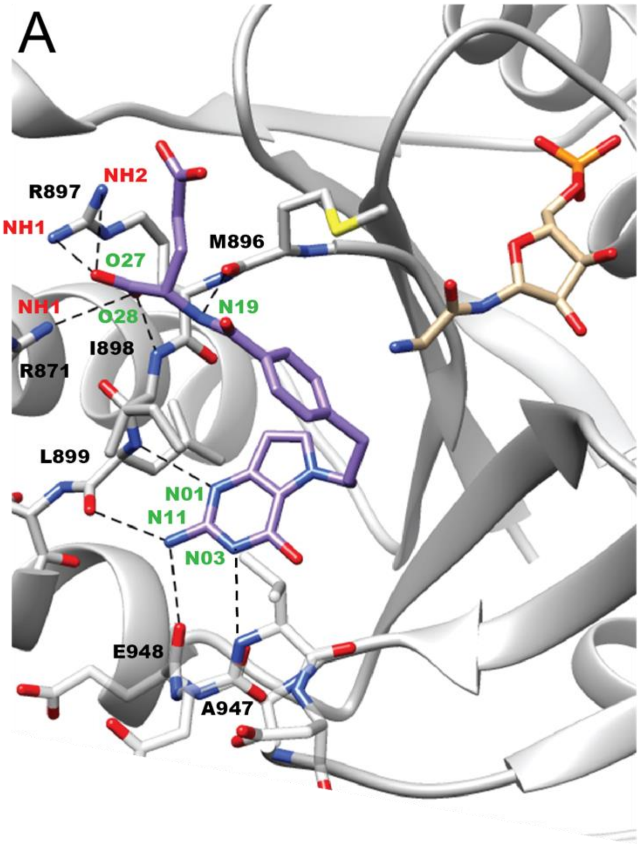

Atom 1	Atom 2	Distance (Å)
Leu899 N	Cmp2 N01	3.27
Leu899 O	Cmp2 N11	3.14
Glu948 O	Cmp2 N11	3.53
Met896 O	Cmp2 N19	3.04
Ala947 O	Cmp2 N03	3.39
Ile898 N	Cmp2 O27	2.67
Arg871 NH1	Cmp2 O27	3.42
Arg871 NH2	Cmp2 O28	3.64
Arg897 NH2	Cmp2 O27	3.80
Arg897 NH1	Cmp2 O28	3.76

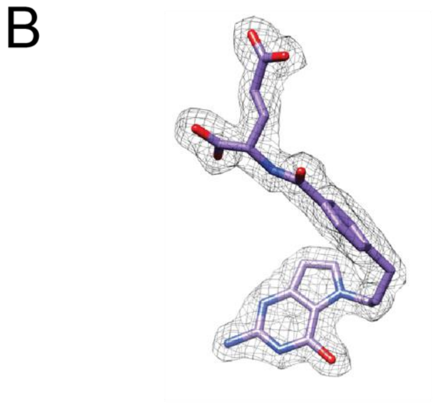

Figure S5. Crystal structure of Compound 2 bound in the folate binding pocket of GARFTase. (A) Hydrogen-bond and charge-charge interactions between **Compound 2** and GARFTase are shown as dashed lines. Atoms that participate in longer distance charge-charge interactions are labeled in red. GARFTase is shown in ribbon except for interacting side-chain and backbone atoms, which are shown as sticks. Distances between interacting atoms are shown in the table with distances greater than 3.2 Å in red. **(B)** The structure is shown of **Compound 2** with the 2Fo – Fc map density in mesh contoured at 0.7 σ.

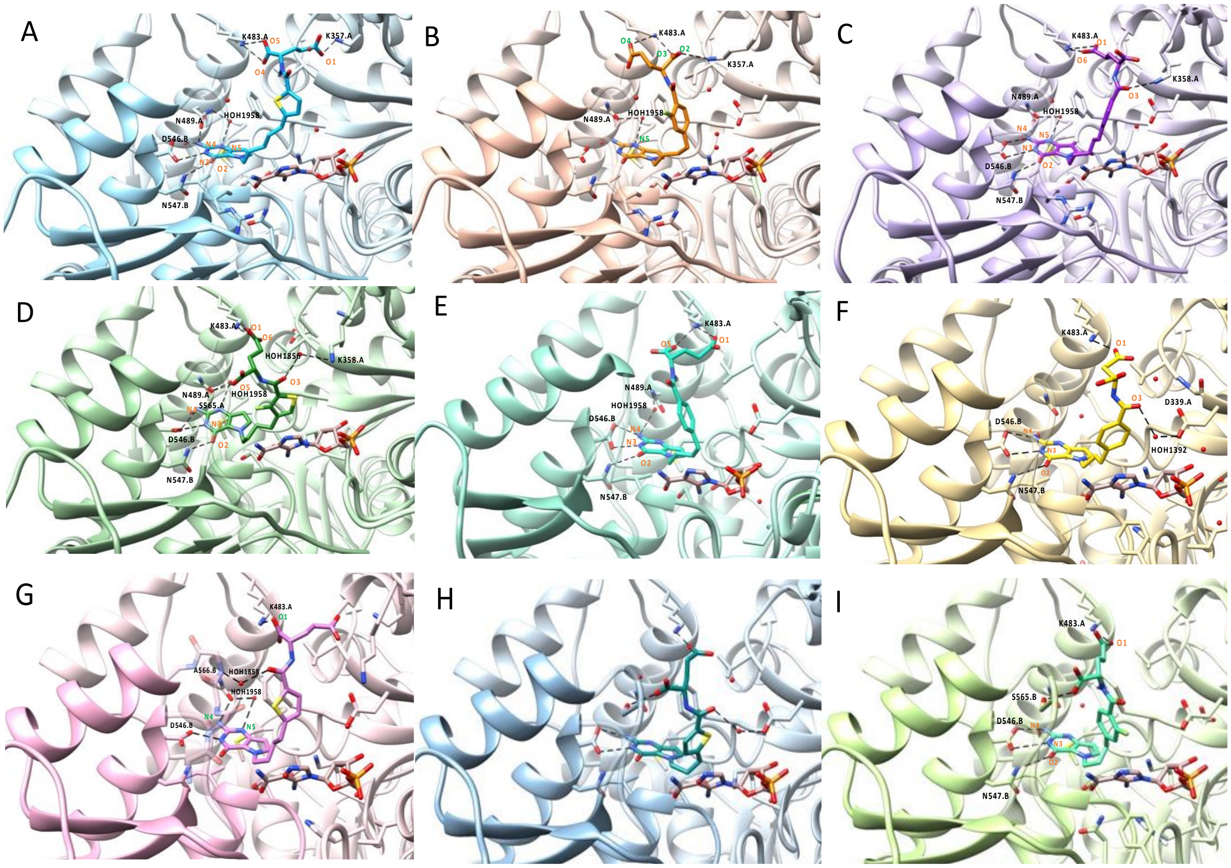

Figure S6. Molecular modeling studies with AICARFTase (ATIC) bound to BW1540U88UD (PDB: 1P4R) (A) Docked pose of Compound 3. Protein is shown as a ribbon with interacting side-chains represented as sticks. Polar contacts are noted with dashed lines. **(B)** Docked pose of Compound 1. **(C)** Docked pose of Compound 11. **(D)** Docked pose of Compound 14. **(E)** Docked pose of Compound 15. **(F)** Docked pose of Compound 2. **(G)** Docked pose of Compound 16. **(H)** Docked pose of Compound 4. **(I)** Docked pose of Compound 13.

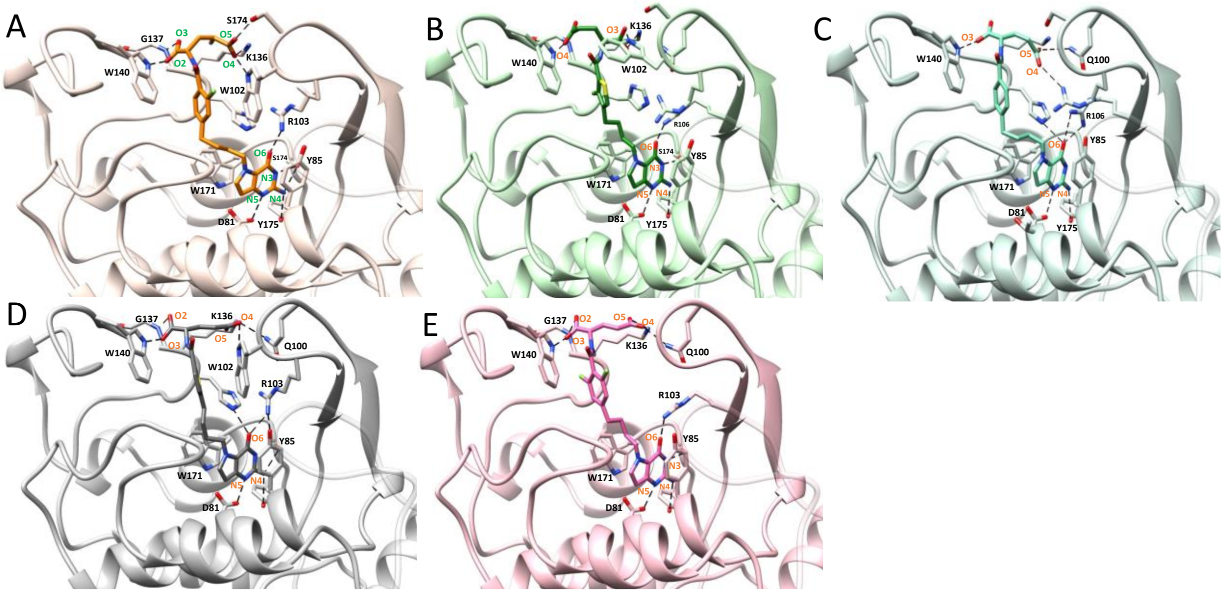

Figure S7. Molecular modeling studies with FRα bound to Compound 10 (PDB: 5IZQ) (A) Docked pose of **Compound 1**. Protein is shown as a ribbon with interacting side-chains represented as sticks. Polar contacts are noted with dashed lines. **(B)** Docked pose of **Compound 14**. **(C)** Docked pose of **Compound 15**. **(D)** Docked pose of **Compound 16**. **(E)** Docked pose of **Compound 12**.

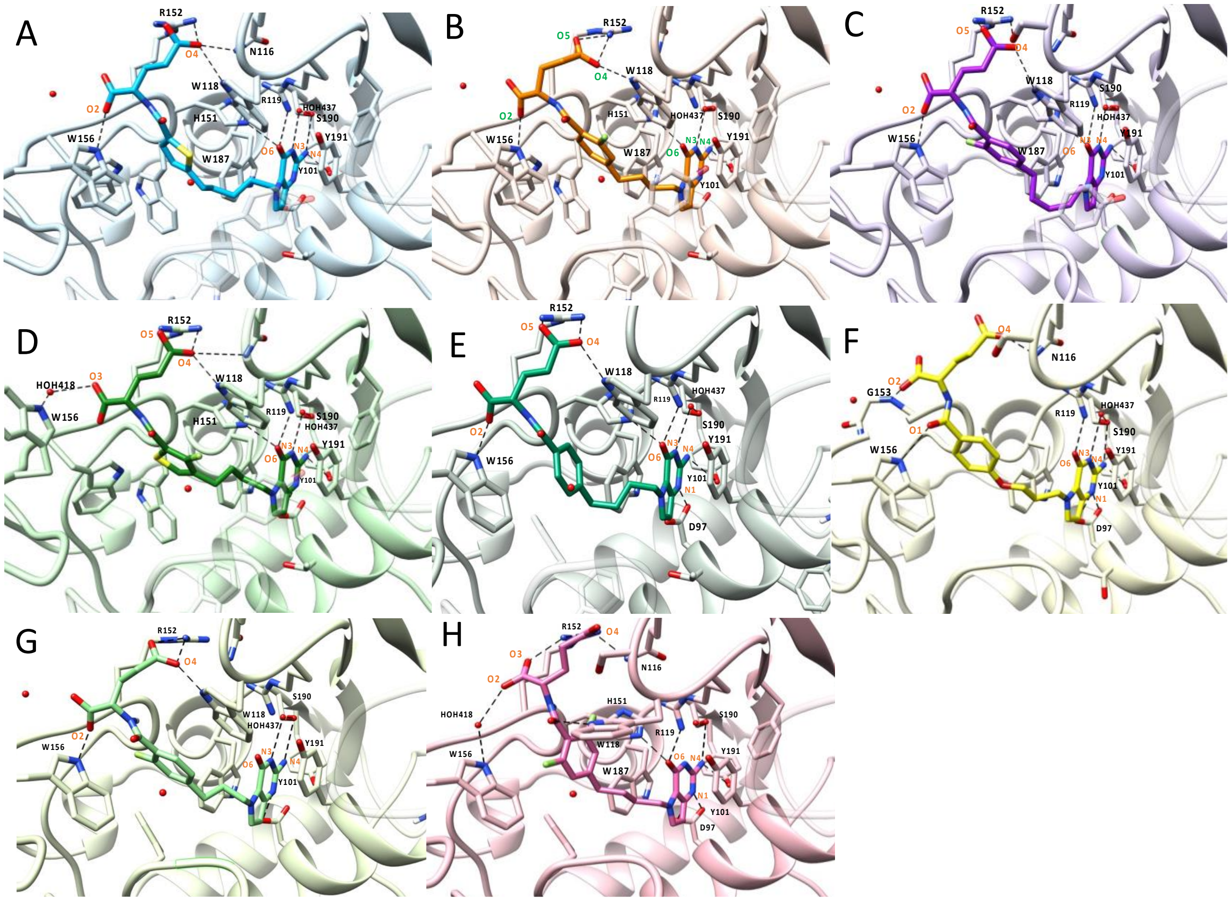

Figure S8. Molecular modeling studies with FRβ bound to PMX (10) (PDB: 4KN2) (A) Docked pose of Compound 3. Protein is shown as a ribbon with interacting side-chains represented as sticks. Polar contacts are noted with dashed lines. **(B)** Docked pose of Compound 1. **(C)** Docked pose of Compound 11. **(D)** Docked pose of Compound 14. **(E)** Docked pose of Compound 15. **(F)** Docked pose of Compound 5. **(G)** Docked pose of Compound 13. **(H)** Docked pose of Compound 12.

A

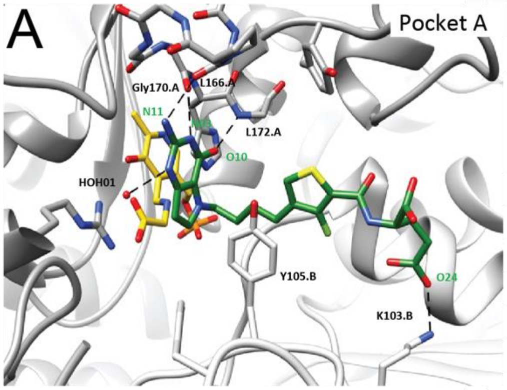

Binding Pocket	Atom 1	Atom 2	Distance (Å)
A	HOH01	Cmp14 N01	3.03
A	Gly170 O.A	Cmp14 N03	2.76
A	Leu172 N.A	Cmp14 O10	3.12
A	Leu166 O.A	Cmp14 N11	2.96
A	Lys103 O.B	Cmp14 O24	2.61
B	HOH02	Cmp14 N01	2.90
B	Gly170 O.B	Cmp14 N03	2.76
B	Leu172 N.B	Cmp14 O10	3.24
B	Leu166 O.B	Cmp14 N11	3.24

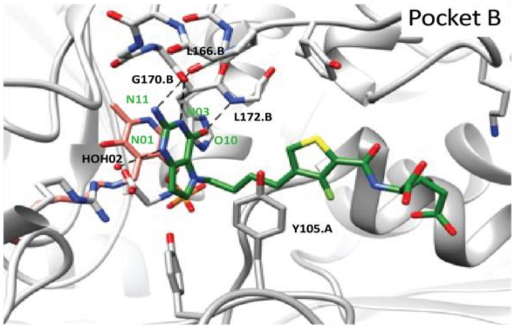

B

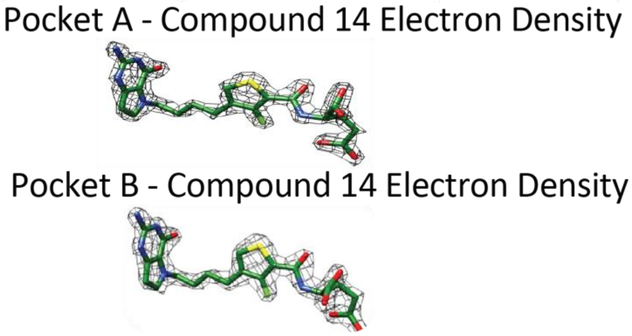

Figure S9. Crystal structure of Compound 14 bound in the folate binding pocket of PLP-loaded SHMT2. (A) Hydrogen-bond and charge-charge interactions between **Compound 14** and SHMT2 are shown as dashed lines. SHMT2 is shown in ribbon except for interacting residues, which are shown as sticks. The distances between interacting atoms in **Compound 14** and the enzyme are shown in the table except the distance between the planes of the phenol ring in Tyr105 to the thiophene ring plane. In pocket A, the distance between the two planes is 3.5 Å. In pocket B, the distance between the two planes is 3.66 Å. Because the binding pocket for SHMT2 contains chains A and B of the dimer, the chain is denoted after the residue atom. In pocket A, an adduct of PLP-bound to free glycine (represented in yellow) was modeled, but in pocket B, the electron density supported that PLP bound to Lys280 with free glycine (represented in salmon) be modeled. Detailed SHMT2: inhibitor contacts for these compounds are provided in the table. **(B)** The structure is shown of **Compound 14** with the 2Fo – Fc map density in mesh contoured at 0.7 σ.

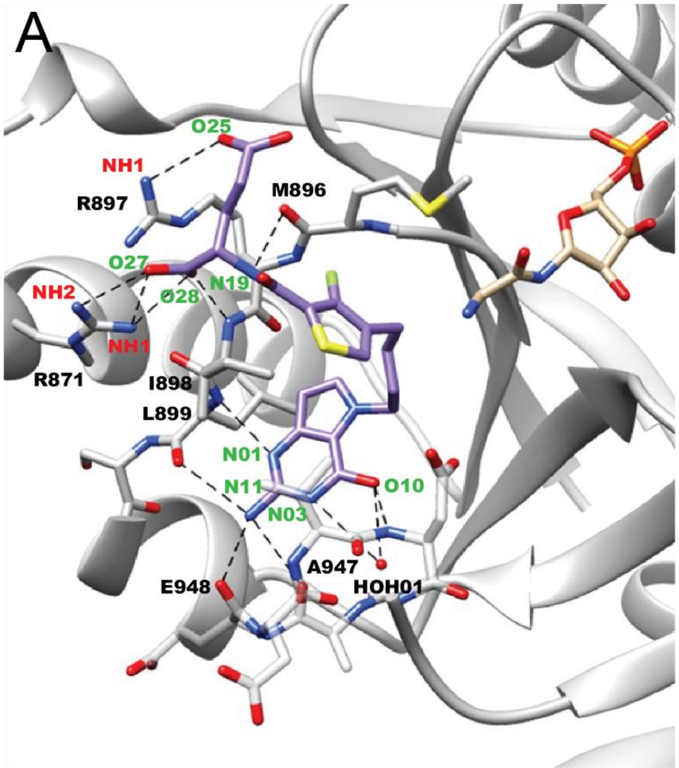

Atom 1	Atom 2	Distance (Å)
Leu899 N	Cmp14 N01	3.19
Leu899 O	Cmp14 N11	3.84
Glu948 O	Cmp14 N11	3.46
Met896 O	Cmp14 N19	3.84
Ala947 O	Cmp14 N03	3.18
Ile898 N	Cmp14 O27	2.98
HOH01	Cmp14 N03	3.45
HOH01	Cmp14 O10	2.94
Asp951 N	Cmp14 O10	3.44
Arg871 NH2	Cmp14 O27	3.30
Arg871 NH1	Cmp14 O28	2.95
Arg897 NH2	Cmp14 O25	3.50

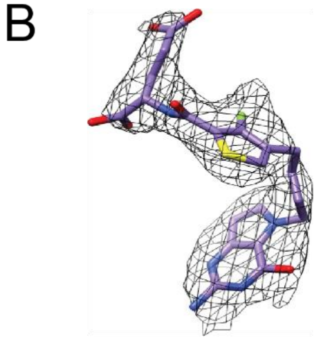

Figure S10. Crystal structure of Compound 14 bound in the folate binding pocket of GARFTase. (A) Hydrogen-bond and charge-charge interactions between **Compound 14** and GARFTase are shown as dashed lines. Atoms that participate in longer distance charge-charge interactions are labeled in red. GARFTase is shown in ribbon except for interacting side-chain and backbone atoms, which are shown as sticks. Distances between interacting atoms are shown in the table with distances greater than 3.2 Å in red. **(B)** The structure is shown of **Compound 14** with the 2Fo – Fc map density in mesh contoured at 0.7 σ.

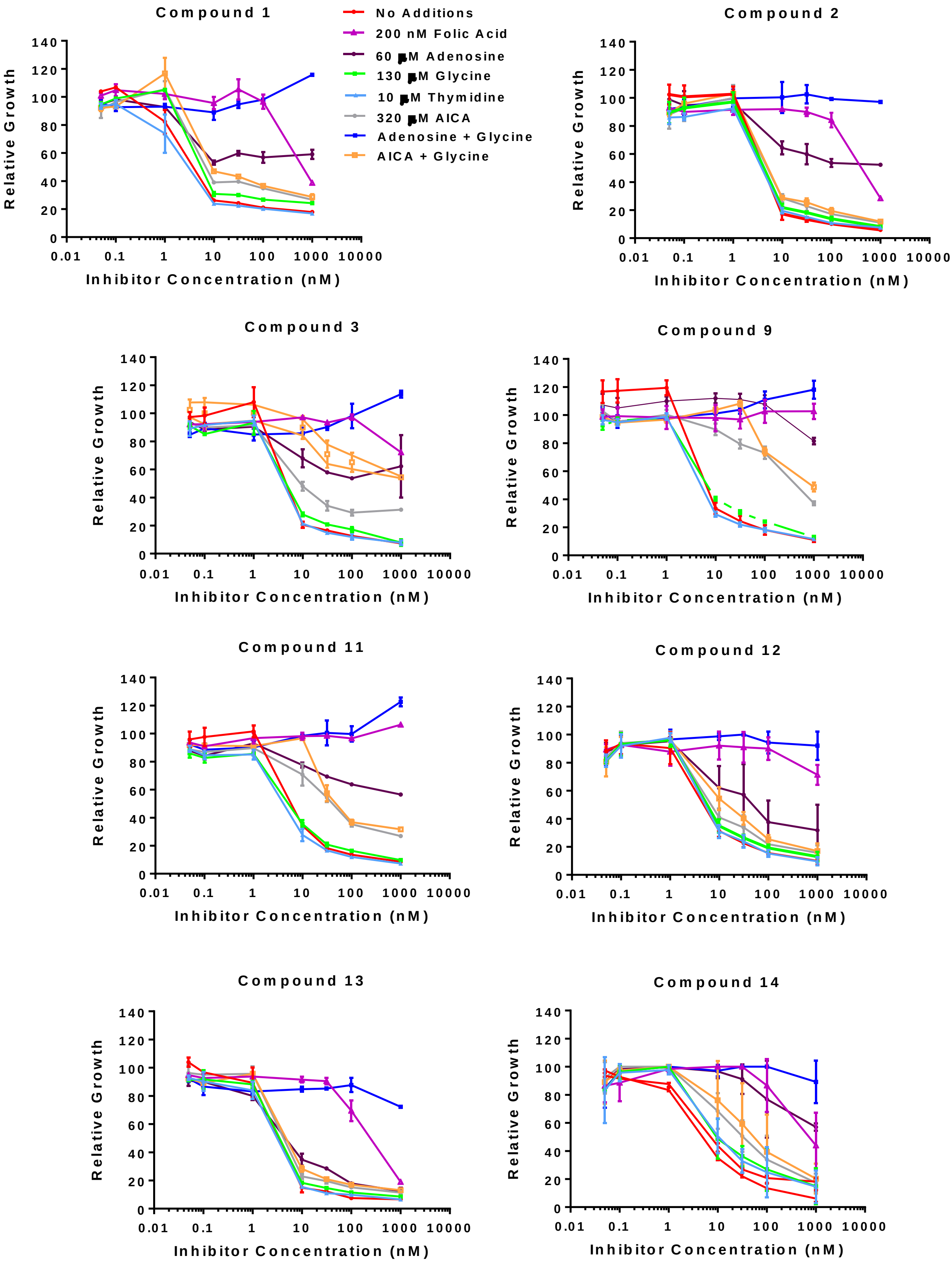
Compound 1
No Additions
200 nM Folic Acid
60 μM Adenosine
130 μM Glycine
10 μM Thymidine
320 μM AICA
Adenosine + Glycine
AICA + Glycine
Relative Growth
Inhibitor Concentration (nM)
Compound 2
Compound 3
Compound 9
Compound 11
Compound 12
Compound 13
Compound 14

Figure S11. *In vitro* antitumor efficacy and identification of targeted pathways and enzymes by novel pyrrolo[3,2-*d*]pyrimidine analogs. Dose-response growth inhibition curves are shown for 5-substituted pyrrolo[3,2-*d*]pyrimidine antifolates compared to the established SHMT2 inhibitor **1** for KB cells cultured in complete folate-, serine, and glycine-free RPMI1640 with 2 nM leucovorin, without additions, or in the presence of folic acid (200 nM) adenosine (60 µM), thymidine (10 µM), glycine (130 µM), and/or AICA (320 µM). The results shown are mean values +/-standard deviations for three biological replicates.

Spectral and Other Characterization Data for Compounds 5-8, 12-14

Compound 5: ^{1}H NMR

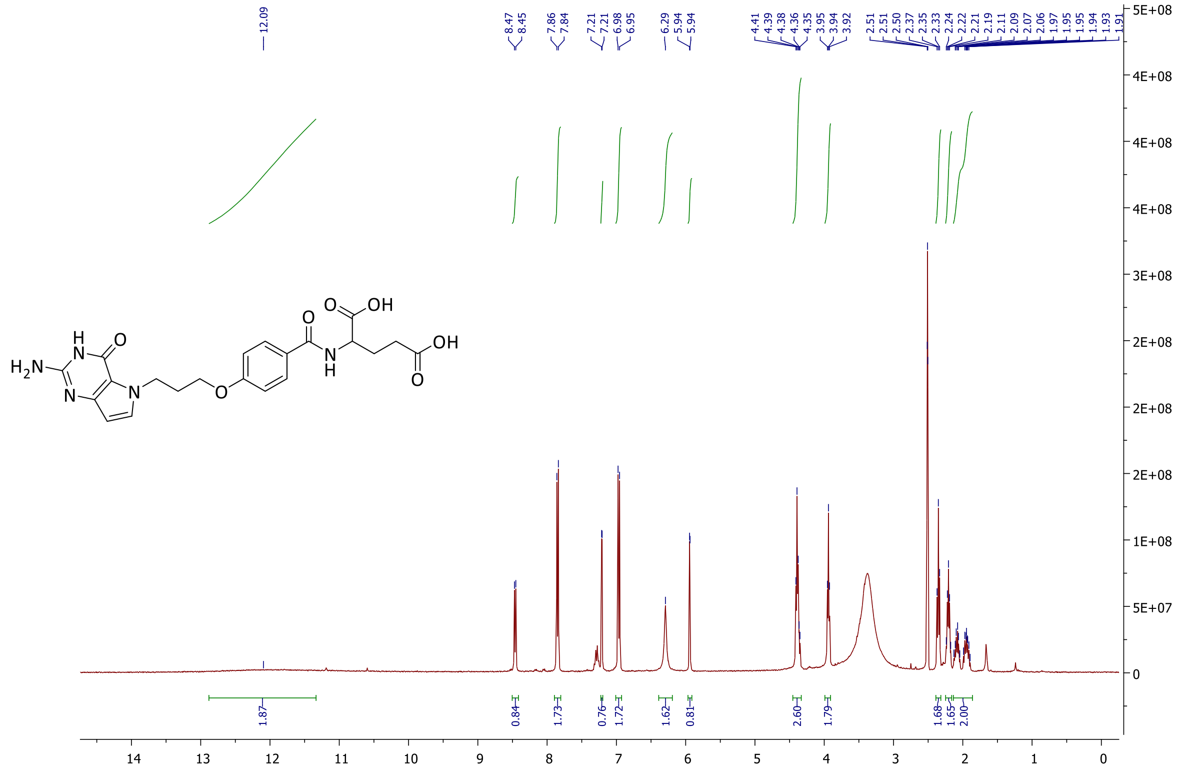

Compound 5: HPLC

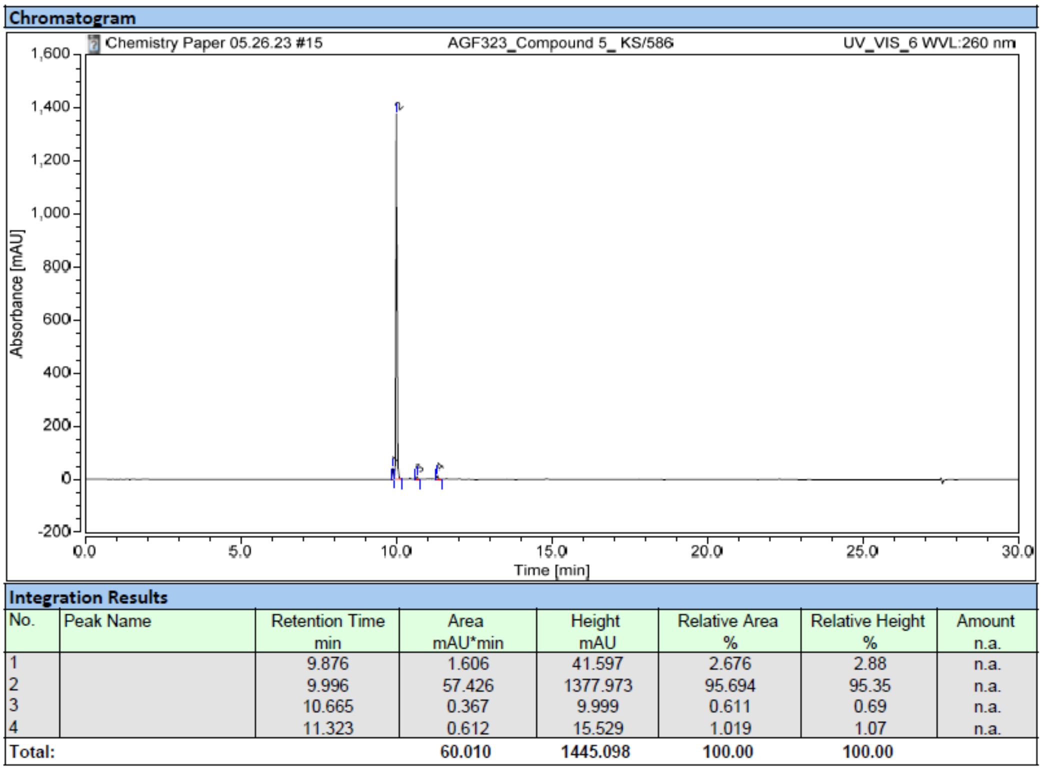

Integration Results

No.	Peak Name	Retention Time min	Area mAU*min	Height mAU	Relative Area %	Relative Height %	Amount n.a.
1		9.876	1.606	41.597	2.676	2.88	n.a.
2		9.996	57.426	1377.973	95.694	95.35	n.a.
3		10.665	0.367	9.999	0.611	0.69	n.a.
4		11.323	0.612	15.529	1.019	1.07	n.a.
**Total:**			**60.010**	**1445.098**	**100.00**	**100.00**	

Compound 5: HRMS

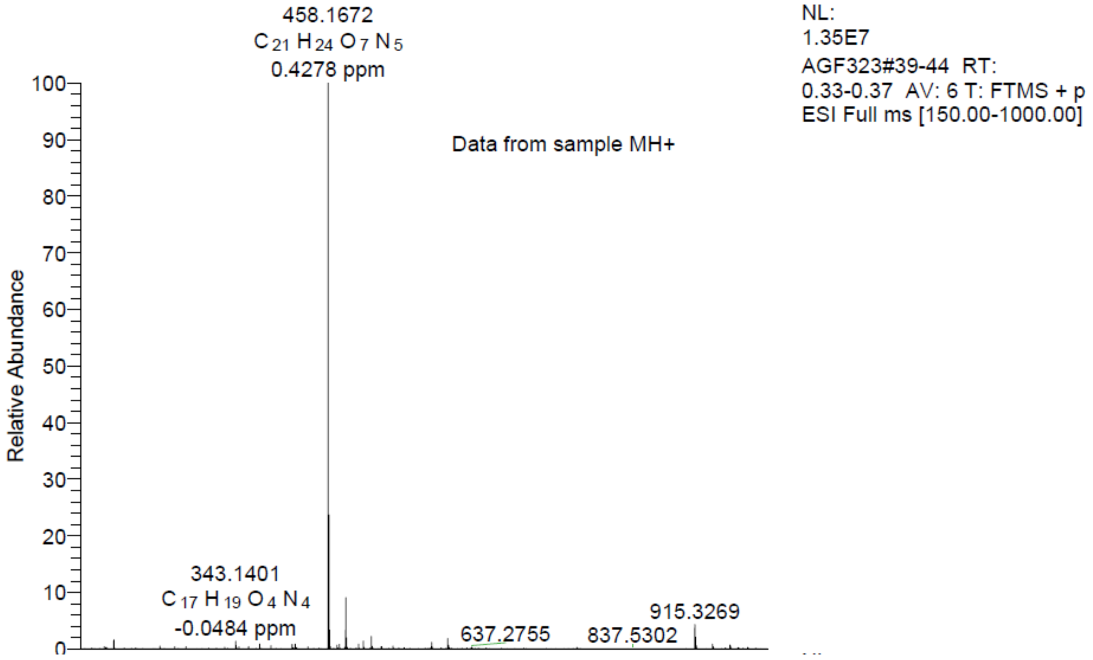

Compound 6: ^{1}H NMR

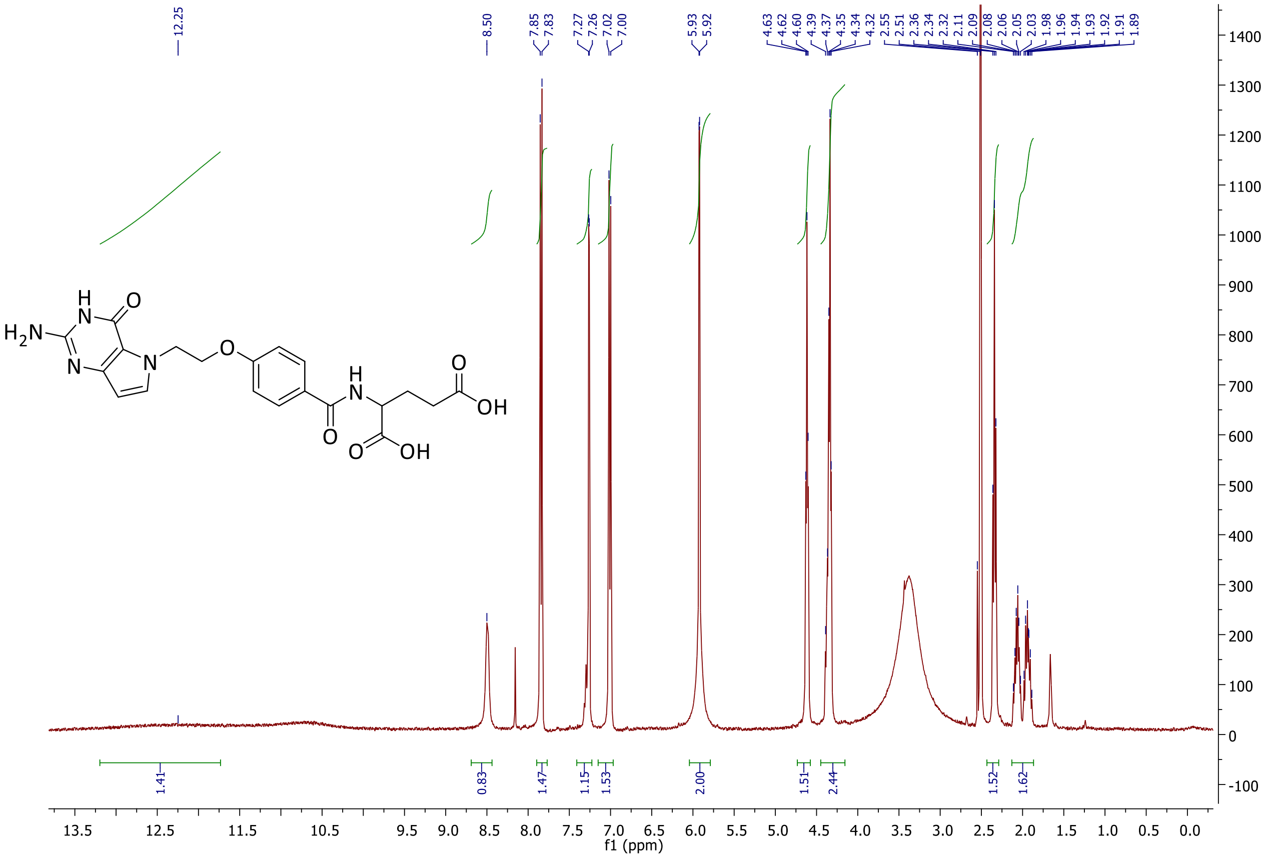

Compound 6: HPLC

Chromatogram

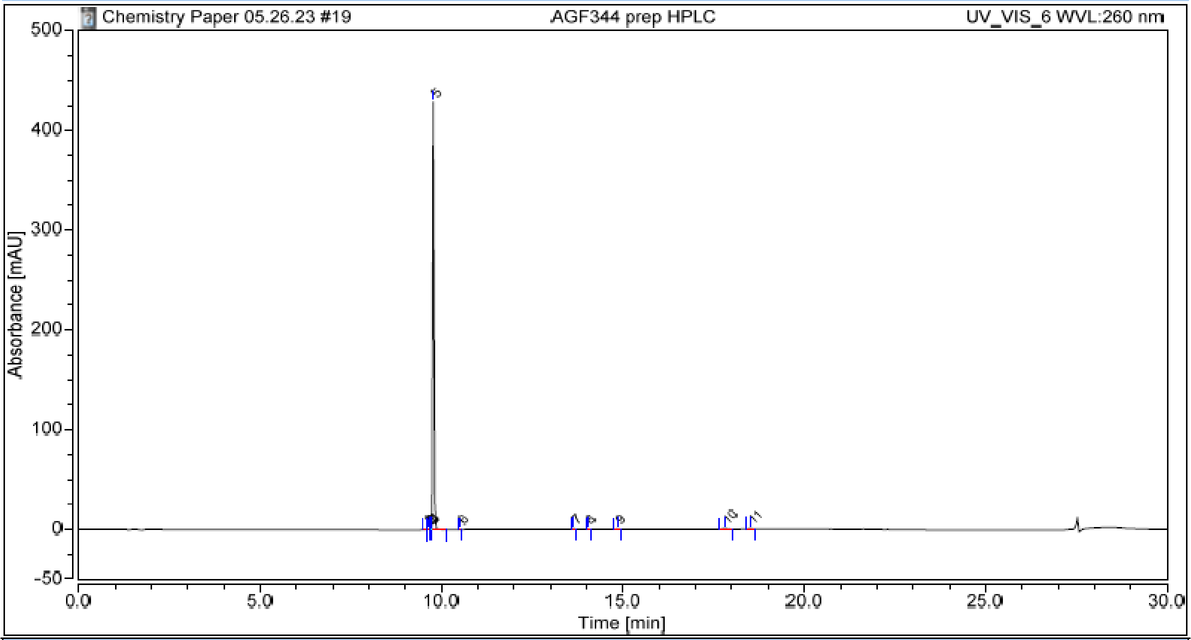

Integration Results

No.	Peak Name	Retention Time min	Area mAU*min	Height mAU	Relative Area %	Relative Height %	Amount n.a.
1		9.588	0.009	0.167	0.060	0.04	n.a.
2		9.650	0.027	0.893	0.177	0.21	n.a.
3		9.693	0.037	1.062	0.240	0.24	n.a.
4		9.729	0.025	1.365	0.163	0.31	n.a.
5		9.777	15.182	428.849	98.195	98.72	n.a.
6		10.505	0.004	0.099	0.024	0.02	n.a.
7		13.633	0.030	0.592	0.197	0.14	n.a.
8		14.036	0.013	0.216	0.082	0.05	n.a.
9		14.839	0.017	0.179	0.112	0.04	n.a.
10		17.810	0.078	0.578	0.505	0.13	n.a.
11		18.499	0.038	0.397	0.246	0.09	n.a.
**Total:**			**15.461**	**434.397**	**100.00**	**100.00**	

Compound 6: HRMS

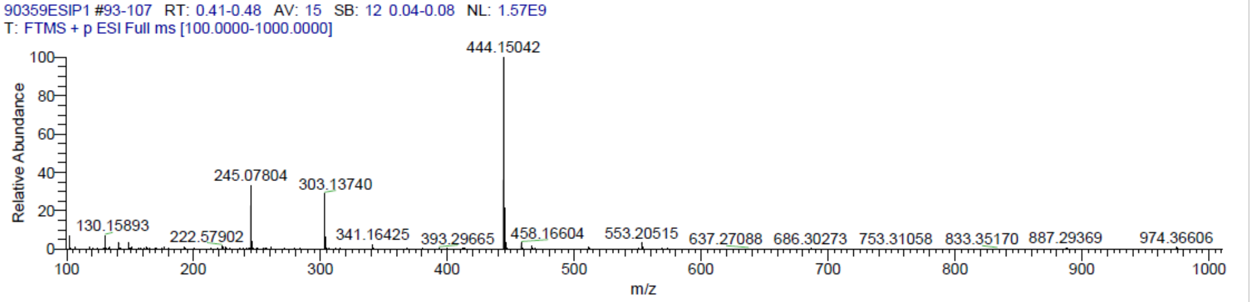

90359ESIP1#93-107 RT: 0.41-0.48 AV: 15
SB: 12 0.04-0.08
T: FTMS + p ESI Full ms [100.0000-1000.0000]
m/z= 420.82524-464.44957

m/z	Intensity	Relative	Theo. Mass	Delta (ppm)	Composition
444.15042	1595194880.0	100.00	444.15137	-2.15	$C_{20} H_{22} O_7 N_5$

Compound 7: ^{1}H NMR

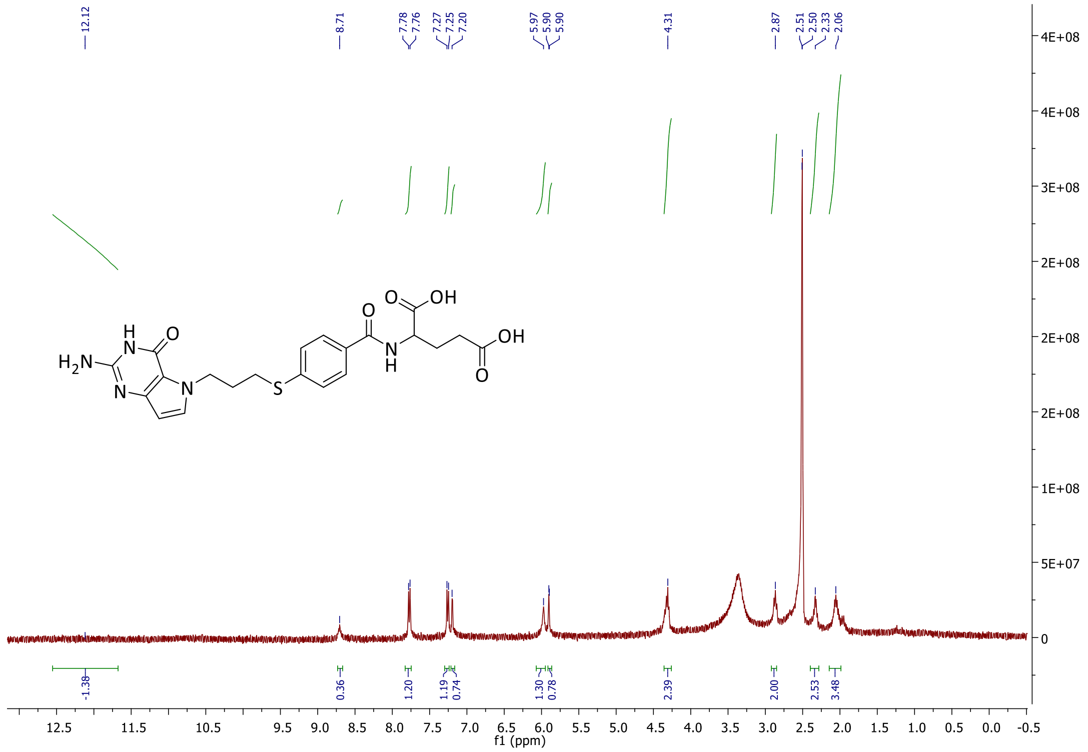

Compound 7: HPLC

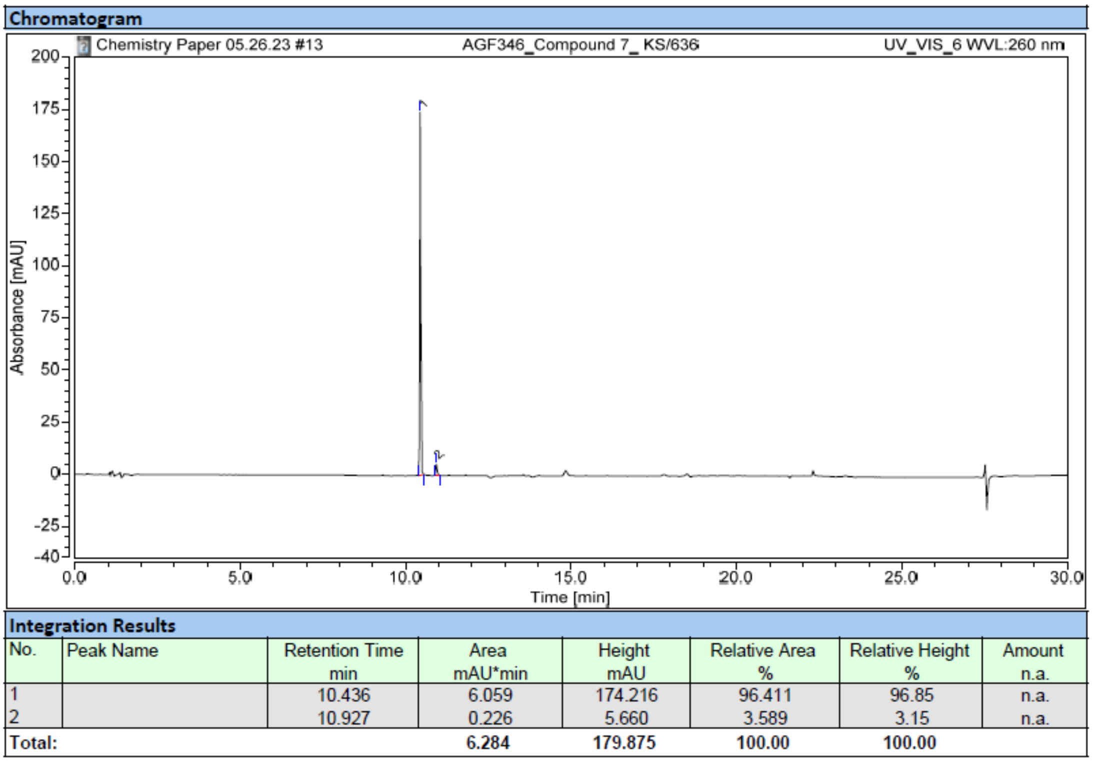

Integration Results

No.	Peak Name	Retention Time min	Area mAU*min	Height mAU	Relative Area %	Relative Height %	Amount n.a.
1		10.436	6.059	174.216	96.411	96.85	n.a.
2		10.927	0.226	5.660	3.589	3.15	n.a.
**Total:**			**6.284**	**179.875**	**100.00**	**100.00**	

Compound 7: HRMS

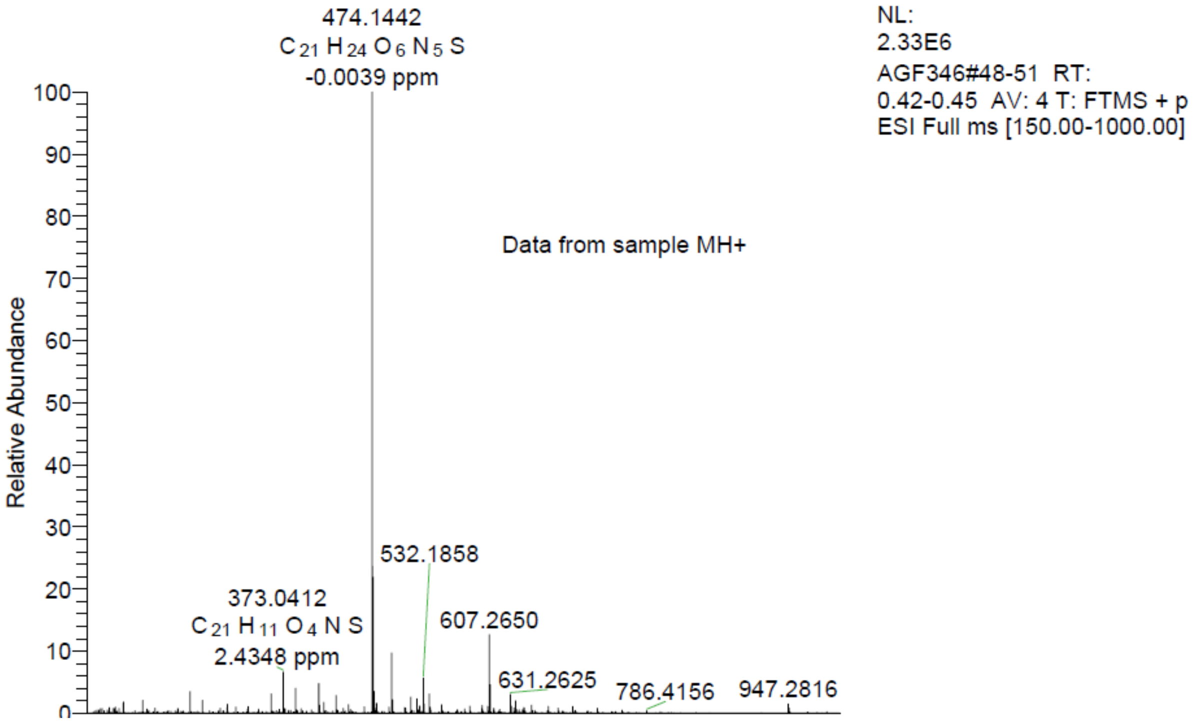

Compound 8: ^{1}H NMR

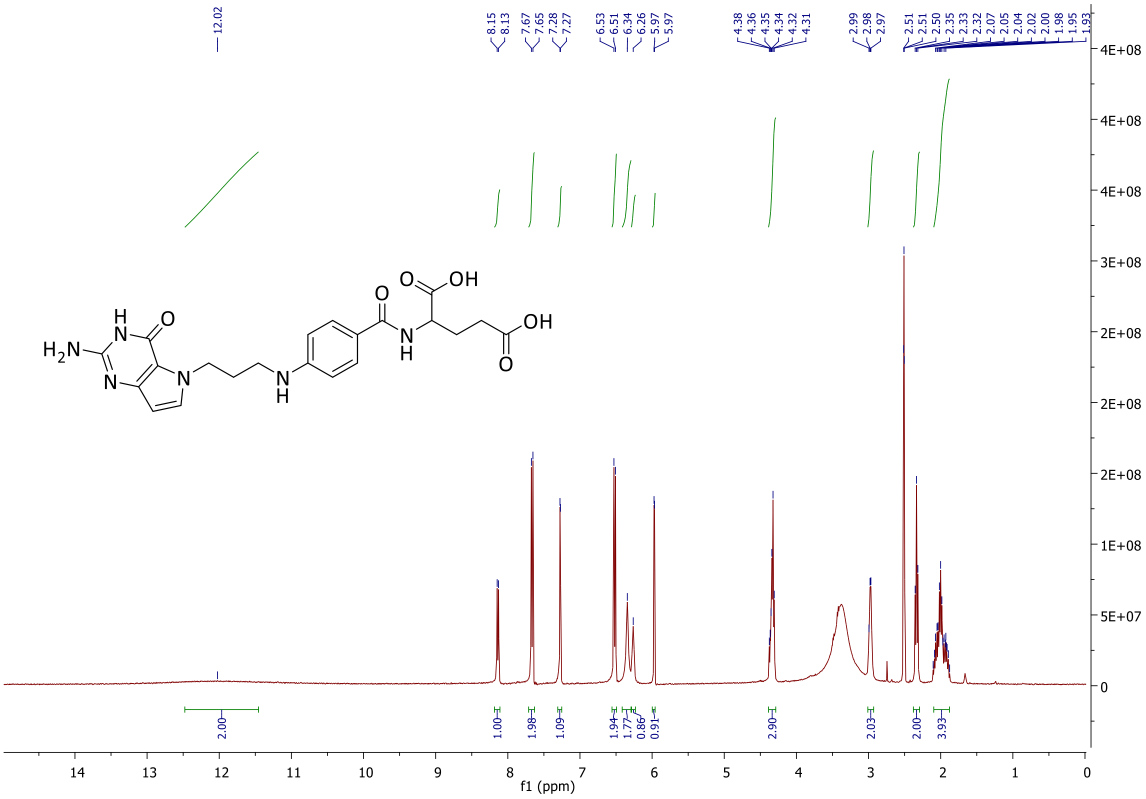

Compound 8: HPLC

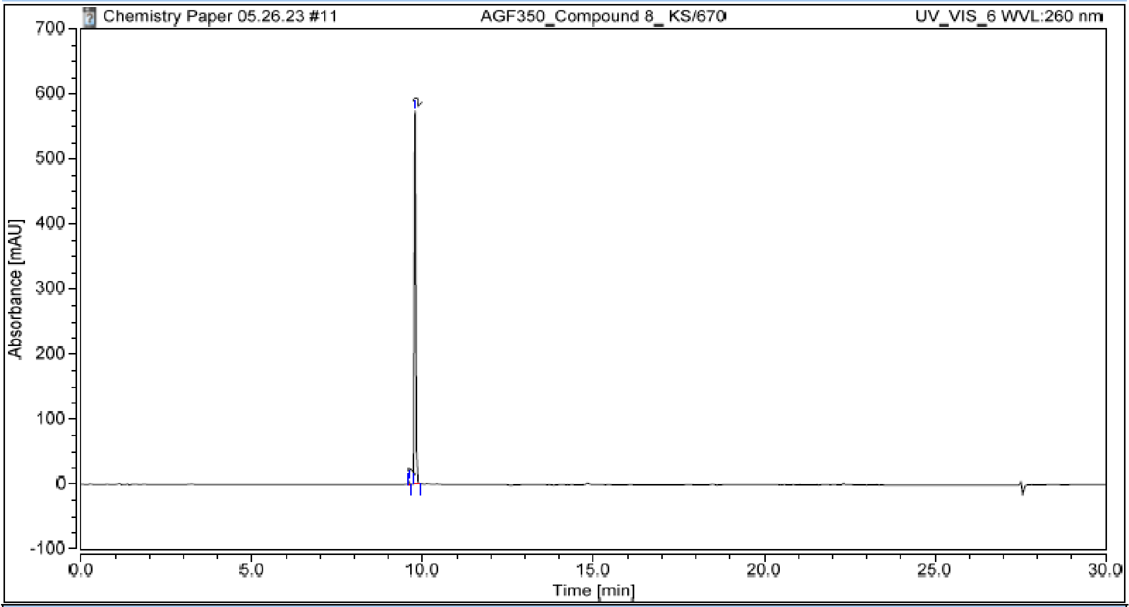

Integration Results

No.	Peak Name	Retention Time min	Area mAU*min	Height mAU	Relative Area %	Relative Height %	Amount n.a.
1		9.588	0.160	5.004	0.725	0.86	n.a.
2		9.778	21.867	573.924	99.275	99.14	n.a.
**Total:**			**22.027**	**578.928**	**100.00**	**100.00**	

Compound 8: HRMS

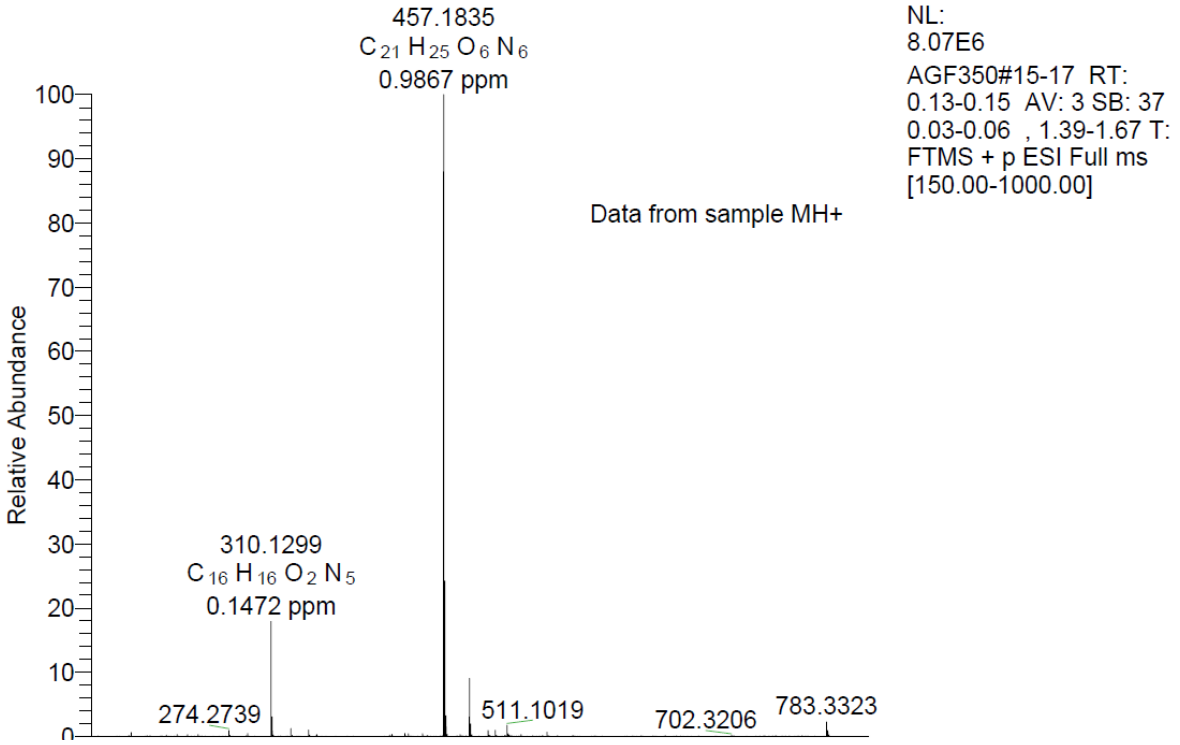

Compound 12: ¹H NMR

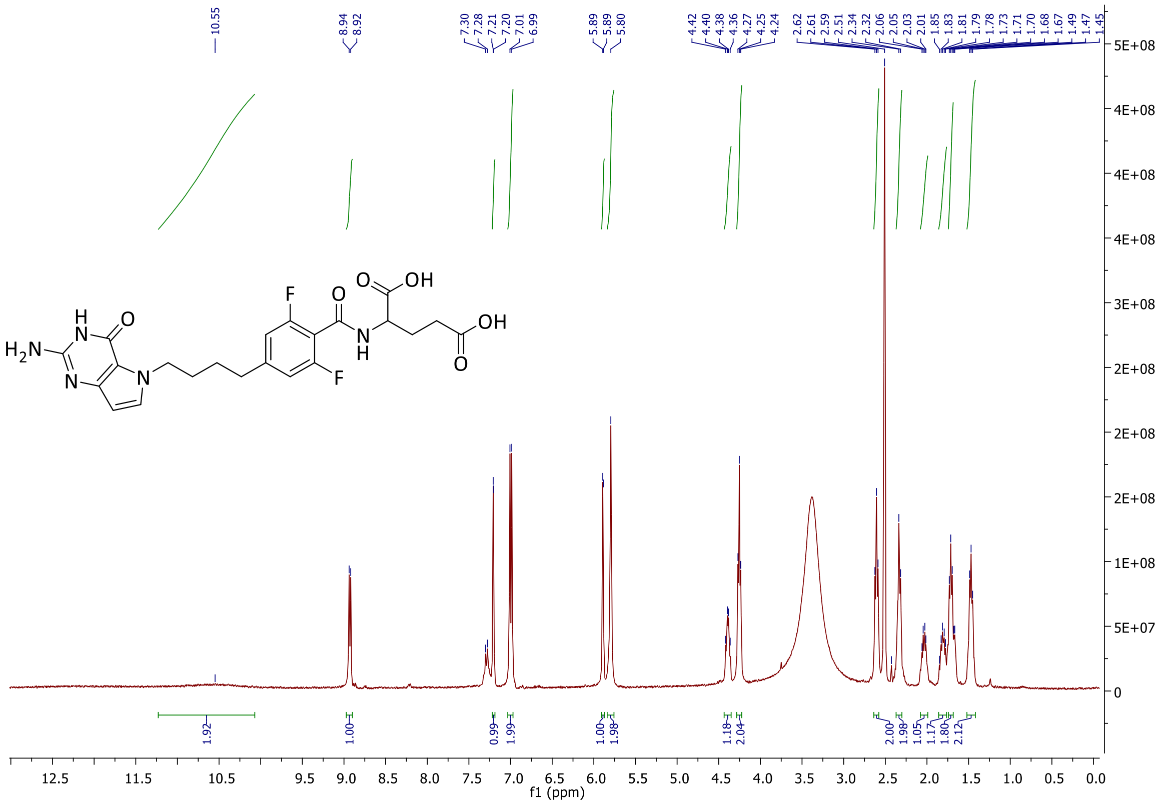

Compound 12: HPLC

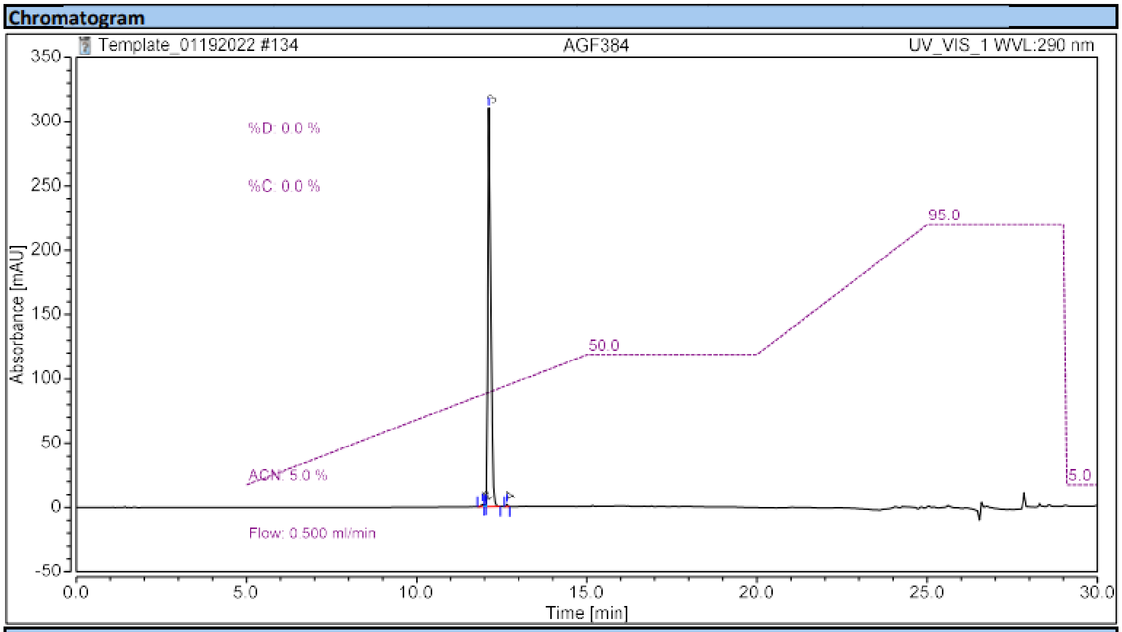

Integration Results

No.	Peak Name	Retention Time min	Area mAU*min	Height mAU	Relative Area %	Relative Height %	Amount n.a.
1		11.937	0.148	2.289	0.493	0.73	n.a.
2		12.017	0.083	1.743	0.275	0.55	n.a.
3		12.123	29.827	309.899	98.962	98.16	n.a.
4		12.660	0.081	1.768	0.270	0.56	n.a.
**Total:**			**30.140**	**315.699**	**100.00**	**100.00**	

Compound 12: HRMS

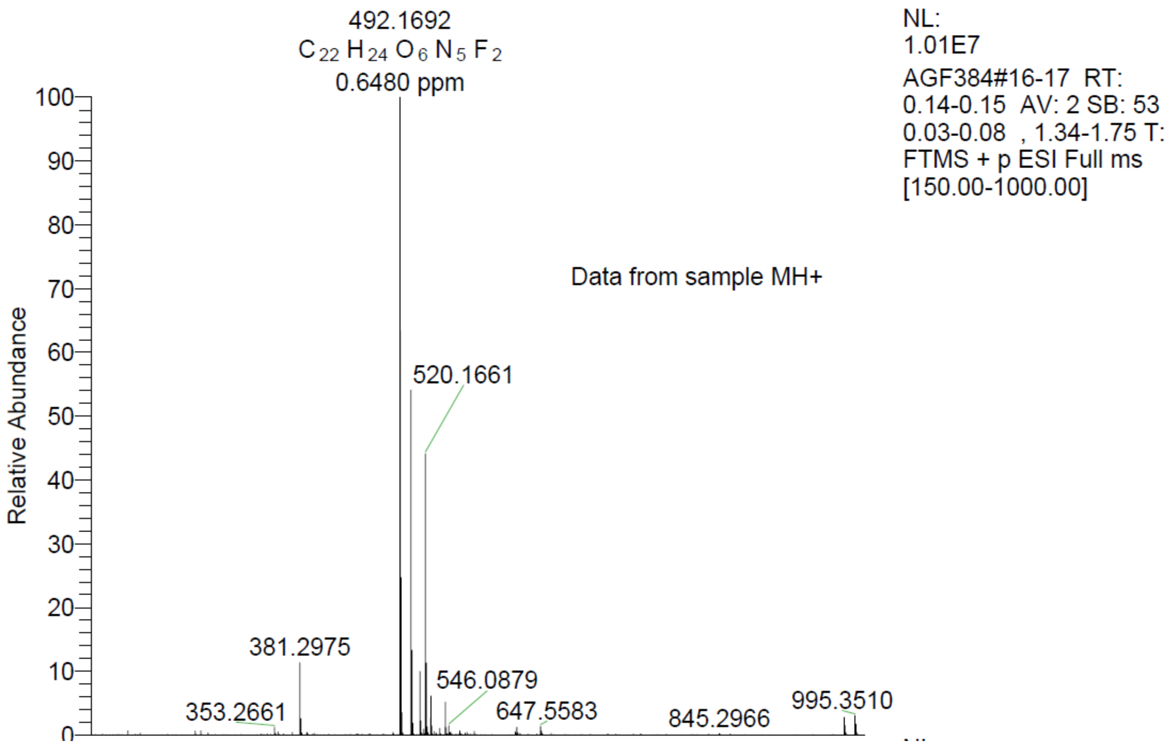

Compound 13: ^{1}H NMR

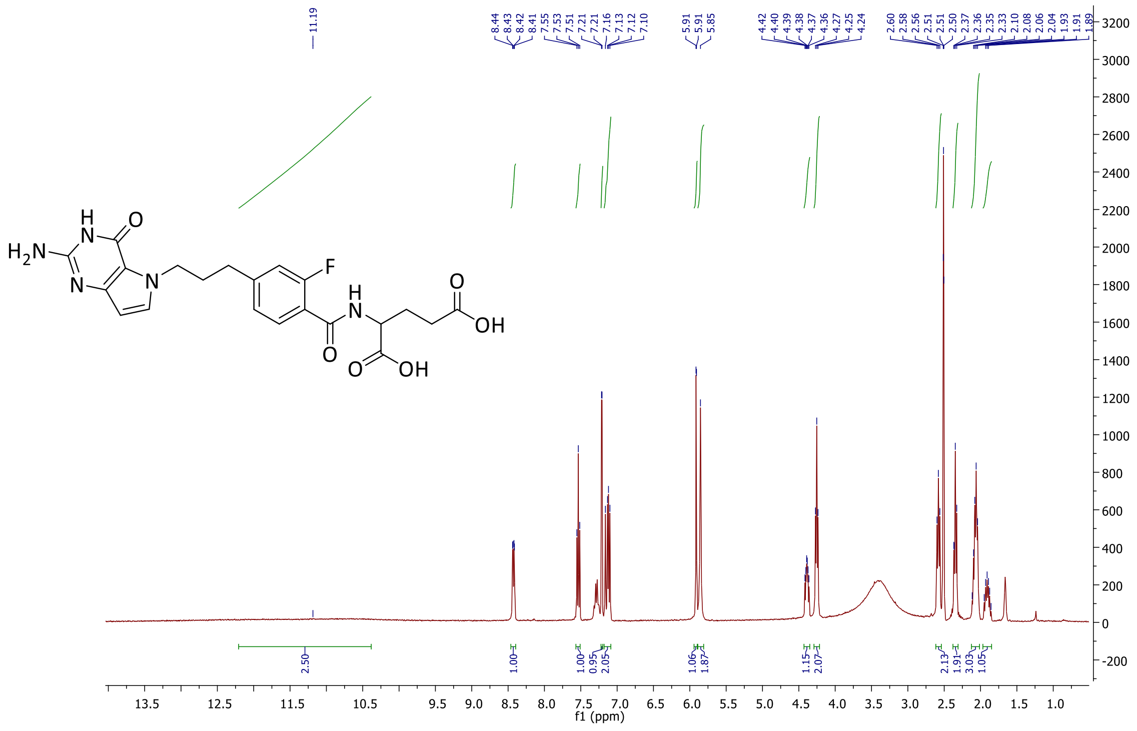

Compound 13: HPLC

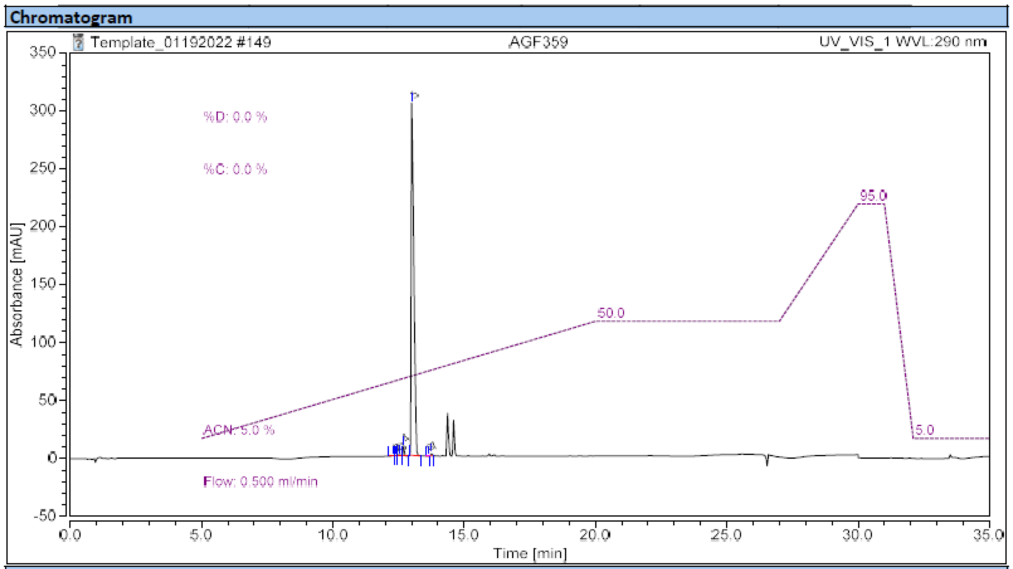

Integration Results

No.	Peak Name	Retention Time min	Area mAU*min	Height mAU	Relative Area %	Relative Height %	Amount n.a.
1		12.287	0.071	0.708	0.190	0.22	n.a.
2		12.390	0.059	0.686	0.157	0.22	n.a.
3		12.547	0.160	1.158	0.425	0.36	n.a.
4		12.690	0.425	8.422	1.127	2.65	n.a.
5		13.000	36.801	304.309	97.644	95.73	n.a.
6		13.620	0.015	0.312	0.041	0.10	n.a.
7		13.740	0.157	2.282	0.417	0.72	n.a.
**Total:**			**37.689**	**317.876**	**100.00**	**100.00**	

Compound 13: HRMS

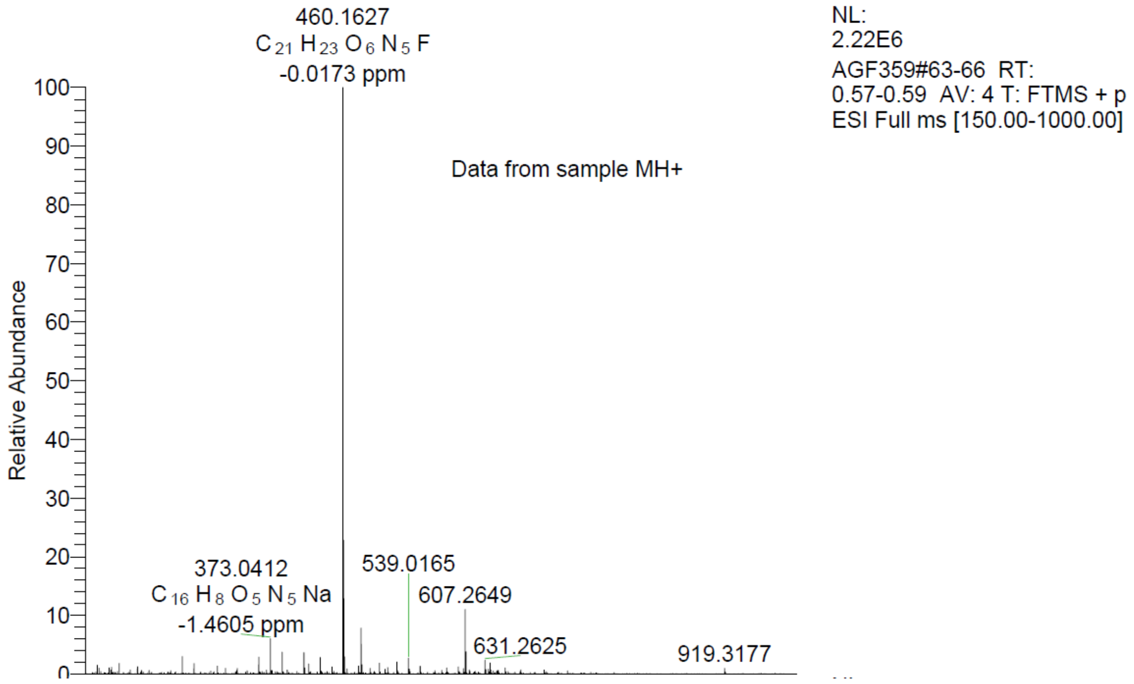

Compound 14: ^{1}H NMR

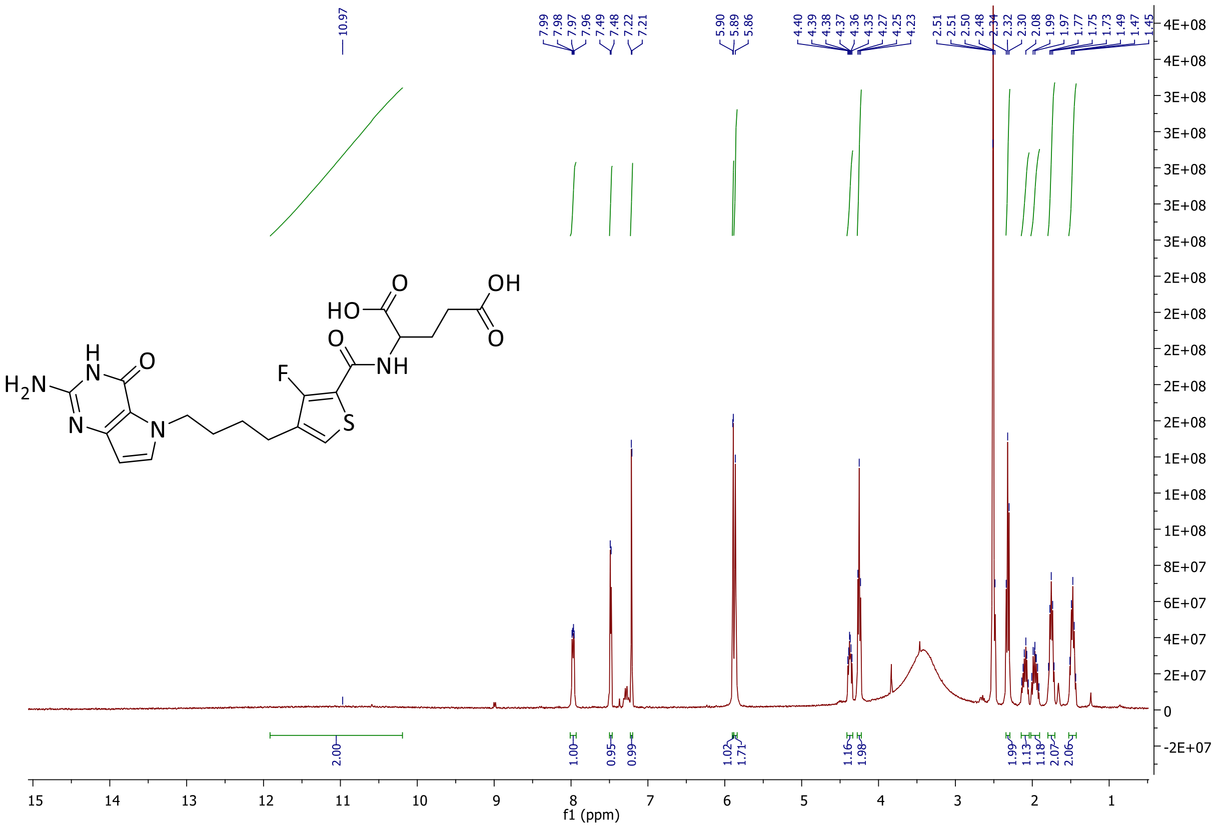

Compound 14: HPLC

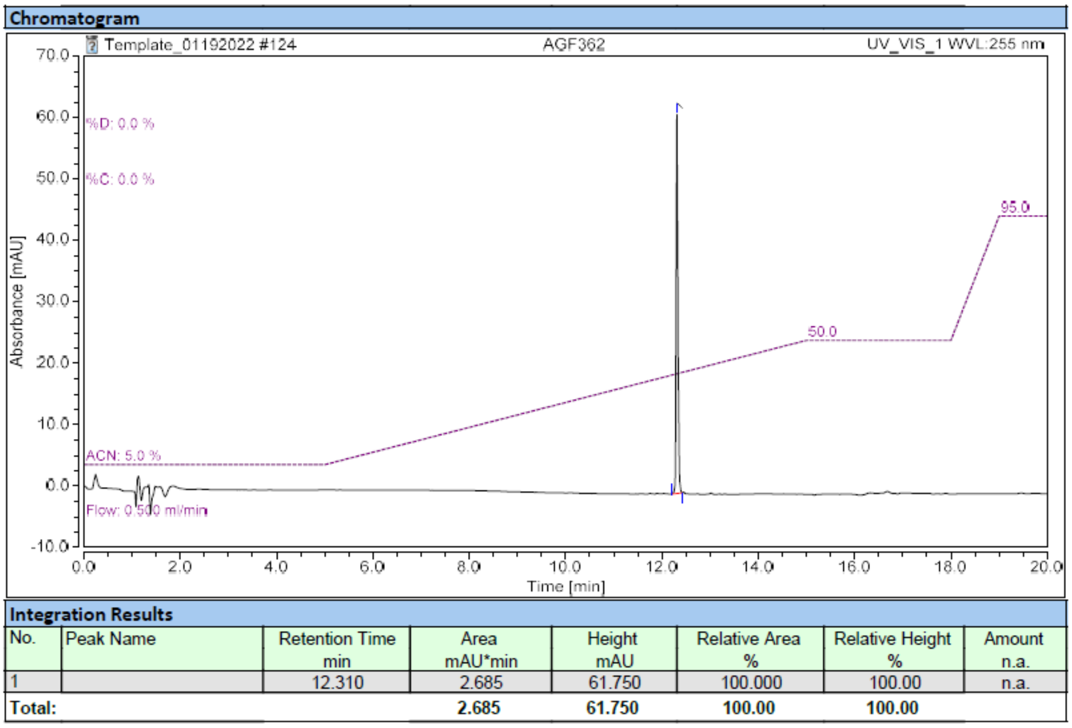

Integration Results

No.	Peak Name	Retention Time min	Area mAU*min	Height mAU	Relative Area %	Relative Height %	Amount n.a.
1		12.310	2.685	61.750	100.000	100.00	n.a.
**Total:**			**2.685**	**61.750**	**100.00**	**100.00**	

Compound 14: HRMS

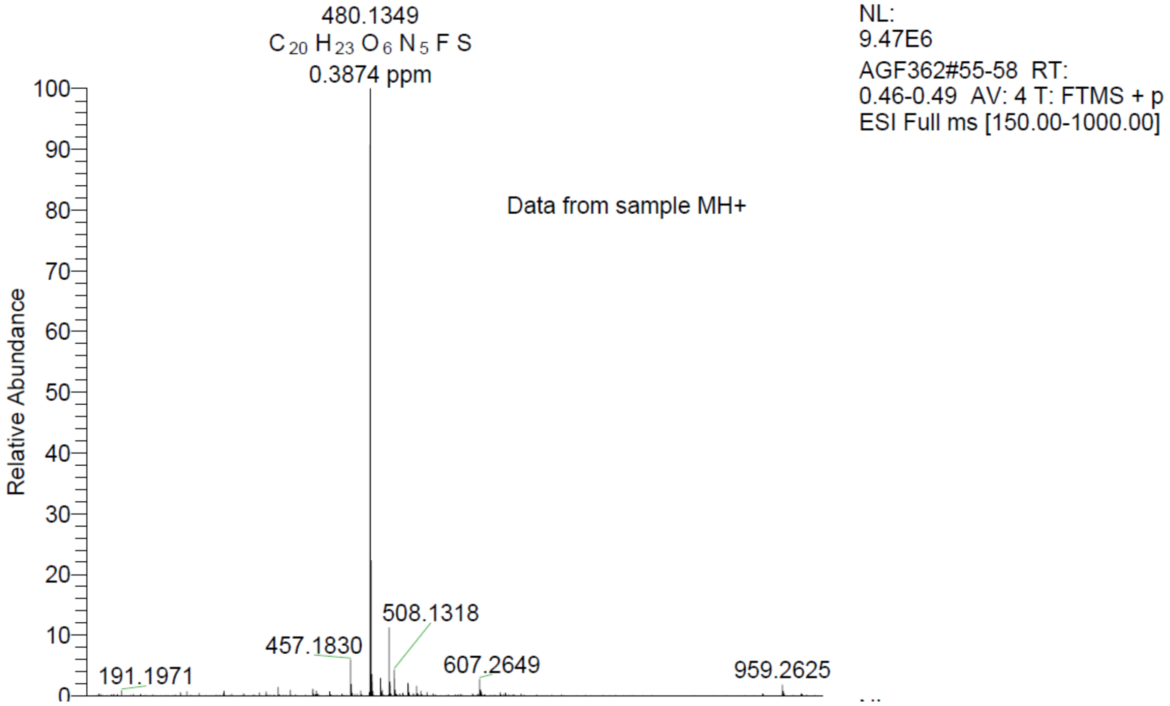

Supplement: Supplementary file 1 — jm3c00763_si_001.pdf [file jm3c00763_si_001.pdf]
